# Focal mechanism variations reveal mechanical controls on segmentation at oceanic transform faults

**DOI:** 10.1038/s41467-026-76407-5

**Published:** 2026-08-15

**Authors:** Fengzhou Tan, Wenyuan Fan, Peter M. Shearer, Mark D. Behn, Jeffrey J. McGuire

**Affiliations:** 1 Scripps Institution of Oceanography, University of California, San Diego, La Jolla, CA USA; 2 Department of Earth and Environmental Sciences, Boston College, Chestnut Hill, MA USA; 3 Department of Geology and Geophysics, Woods Hole Oceanographic Institution, Woods Hole, MA USA

**Keywords:** Seismology, Solid Earth sciences, Geophysics, Tectonics

## Abstract

Oceanic transform faults (OTFs) exhibit concurrent slip behaviors, with creep and characteristic earthquakes on localized patches. At the Gofar transform fault on the East Pacific Rise, magnitude 5.5+ earthquakes are bounded by creeping segments that act as persistent rupture barriers, yet the physical controls on this segmentation remain unclear. Here, from one year of ocean bottom seismometer data, we develop a focal mechanism catalog of 2323 earthquakes on the westernmost Gofar transform fault. This catalog enables robust statistical analyses of along-strike variations in faulting type and mechanical properties. Pure strike-slip earthquakes comprise  ~31% of events; oblique earthquakes are pervasive; pure reverse and normal events also exist. Faulting types correlate with seismicity-inferred fault segmentation, with reverse and oblique-reverse events clustering in rupture barriers. Coulomb failure modeling shows that the pattern is diagnostic of elevated pore-fluid pressure and mechanical weakness within the barriers. The 2008 M6 mainshock increased focal mechanism diversity across the fault, indicating widespread activation of secondary structures through the seismic cycle. Comparisons with the Blanco and Chain transform faults suggest diverse faulting and along-strike mechanism clustering may be common OTF features, implying that local mechanical properties govern segmentation.

## Abstract (web summary)

The authors use thousands of small earthquakes to show that variations in fault zone strength control segmentation of oceanic transform faults and their distinct slip behaviors.

## Introduction

Oceanic transform faults (OTFs) are one of the three fundamental types of plate boundaries that accommodate relative plate motion between offset spreading segments of mid-ocean ridges. Despite their apparent geometric simplicity, OTFs are highly segmented along strike, with adjacent seismic and creeping segments that operate in different slip modes and appear stationary over seismic cycles^1–3^. Moderate to large earthquakes (*M *≥ 5) occur quasi-periodically only on the seismic segments, while creeping segments are ubiquitous and account for the majority of cumulative fault length^2–4^. The along-strike faulting segmentation has been observed at many OTFs, including Gofar^5,6^, Blanco^7,8^, Oceanographer^9^, Charlie-Gibbs^10^, Chain^11^, Romanche^12^, and Discovery^13^ transform faults. Such variation in slip behaviors has been attributed to differences in compositional, structural, mechanical, and hydraulic properties^5,6,10,14–20^, which are inferred from seismicity^5,6,10,11,14^, earthquake stress drops^19^, seismic velocities^11,14,16,17,20^, numerical models^18^, and geological samples^15,21^. However, it has not been possible to test whether these inferred property differences produce systematic, detectable signatures in earthquake faulting types along individual transform faults.

Earthquake focal mechanisms are a direct probe of fault geometry, stress, and slip direction at seismogenic depths^22,23^, and can reveal whether faulting type varies systematically between seismic and creeping segments. Yet focal mechanism catalogs at OTFs have historically been too small to support such analysis, largely due to the lack of dense near-fault observations^24–26^. Early studies reported only a handful of mechanisms per transform^27–30^. More recent ocean bottom seismometer (OBS) deployments have expanded catalogs to tens or, in some cases, fewer than 200 mechanisms per OTF^9,11,31–34^. Previous focal mechanism studies have documented predominantly strike-slip faulting, accompanied by various other mechanisms, and revealed differences in faulting across transform segments, ridge-transform intersections, mid-ocean ridges, and pull-apart basins^9,11,26,32,33^. However, with catalogs of this size, analyses have necessarily focused on broad tectonic features. At smaller scales, the segmentation between seismic and aseismic sections within individual transform faults is still commonly inferred indirectly from teleseismic observations, microseismicity patterns, and seafloor morphology^7,10–12^. Existing focal mechanism catalogs remain insufficient to resolve systematic faulting patterns that might be linked quantitatively to fault zone mechanics. More direct in situ measurements of fault zone physical properties, such as electrical conductivity, are also very limited at OTFs^35,36^. Consequently, the mechanisms that sustain such stable segmentation remain debated.

**Fig. 1:**
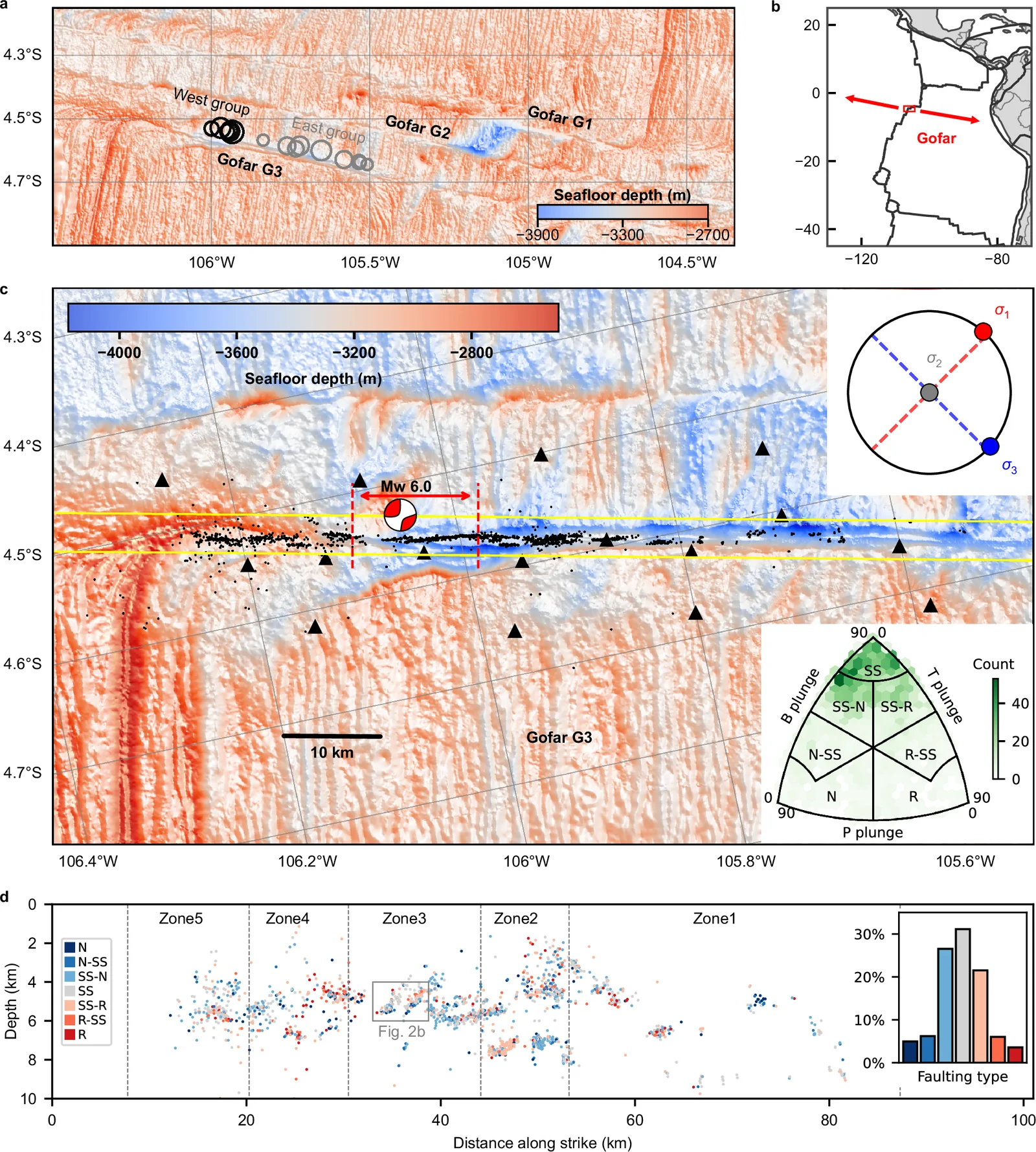
Study area, seismicity, and the focal mechanism catalog. **a** Bathymetry of the Gofar region with three segments (G1 to G3) between the mid-ocean ridges. The black and gray circles show M5.5+ earthquakes in G3 west and east from 1997–2019^2^, respectively. **b** Regional tectonic setting of the Gofar transform fault along the East Pacific Rise. Plate boundaries are from the PB2002 model^79^. **c** Map view of ocean bottom seismometer stations (black triangles) and focal mechanism catalog earthquakes (black dots). The map is rotated 12.5° counterclockwise to align with the fault strike. The focal mechanism shows the 2008 M6 mainshock moment tensor solution^45^. The yellow lines show the 4-km-wide fault zone used in all cross section plots and along-strike analysis. **Top inset**, Inverted maximum (*σ*_1_), intermediate (*σ*_2_), and minimum (*σ*_3_) principal stress directions, shown by the red, gray, and blue circles, respectively, and modeled *σ*_1_ and *σ*_3_ directions, shown by the red and blue dashed lines, respectively^60^. Bottom inset, Focal mechanism classification scheme based on the plunges of the compressional (P), tensional (T), and null (B) axes^42^. **d** Along-strike cross section of the Gofar G3 segment (vertical exaggeration × 2), with earthquakes color-coded by faulting type. The rectangle marks the area in Fig. 2b. The zones follow Gong and Fan (2022)^5^. Inset, Histogram of faulting types. Abbreviations: N normal; N-SS normal-strike-slip; SS-N strike-slip-normal; SS strike-slip; SS-R strike-slip-reverse; R-SS reverse-strike-slip; R reverse.

The Gofar transform fault at the East Pacific Rise slips at a rate of ~140 mm/yr^37^ and exhibits regular characteristic earthquakes and creeping segments^1,5,6,14^ (Fig. 1a, b). Its westernmost strand (G3) has two seismic segments that each rupture, on average, in M5.5+ earthquakes every five years^1,2^ (Fig. 1a). These two seismic segments are separated by a creeping segment that has halted the M5.5+ earthquakes for at least the last six observed seismic cycles^2^, acting as a persistent rupture barrier. This barrier also hosts prominent foreshock sequences, suggesting its involvement in nucleating the mainshocks^5,14^. In 2008, 16 OBS were deployed at the G3 Gofar fault with a spatial coverage of ~80 km along strike. The experiment captured an M6 mainshock westward of the barrier zone and an active foreshock sequence in the barrier zone lasting over nine months^14^. Spatiotemporal evolution of microearthquakes suggests that G3 is segmented into five zones: two separate rupture zones (Zones 1 and 3) capable of M6 earthquakes, a barrier zone separating the rupture zones (Zone 2), a transition zone (Zone 4), and a swarm zone (Zone 5) connected to the ridge^5,14^. While the controlling factors behind these distinct seismic behaviors in neighboring zones remain unclear, systematic differences in bathymetric complexity^38^, seismic velocity^14,16,17,20^, earthquake stress drop^19^, and electrical resistivity^35^ have been reported between the rupture and barrier zones. A central question is whether the stress regime and faulting type also exhibit along-strike variations that correlate with this segmentation, and, if so, what this reveals about the underlying mechanical properties of the fault.

Here, we analyze the 2008 OBS data (Fig. 1c) to develop a microearthquake (mostly M < 3) focal mechanism catalog for the G3 Gofar fault. We obtain solutions for 2323 earthquakes (Supplementary Data 1), an order of magnitude more than in individual published OTF focal mechanism catalogs (Table S1), and use them to investigate the interplay between faulting types, fault zone mechanics, and the earthquake cycle. This catalog allows us to show that faulting types vary systematically along strike in a pattern that mirrors the seismicity-inferred segmentation between rupture zones and creeping barriers. Through Coulomb failure modeling, we demonstrate that this spatial pattern can be explained by along-strike variations in pore-fluid pressure, linking faulting type statistics directly to the mechanical state of the fault. The catalog further reveals that this fault network is not static: the 2008 M6 mainshock activated a diverse set of secondary structures across the entire G3 strand, producing measurable shifts in the distribution of faulting types from the late interseismic period into the early postseismic period. Comparisons among different transform faults suggest that focal mechanism diversity may be related to slip rate and age offset, and along-strike clustering of mechanism types may be a common feature of OTFs, pointing to a general role for local mechanical properties in governing the segmentation that controls the occurrence of large earthquakes on these faults.

## Results

### Diverse focal mechanisms in a uniform stress regime

Earthquake focal mechanisms can inform fault geometry and slip direction^22^, and can be used to study the stress field at depth^23^. We start with all 31,251 cataloged microearthquakes from Gong and Fan (2022)^5^, and use two deep-learning models, PickBlue^39^ and RPNet^40^, to pick the P wave onset times and determine their polarities, respectively (Figs. S1 and S2). The P wave polarities that pass the quality control criteria (see “Methods” and Text S1) are then used to invert for focal mechanisms using SKHASH^41^. In total, we obtain focal mechanisms of 2323 events (Fig. 1c, d); see “Methods” for details.

**Fig. 2:**
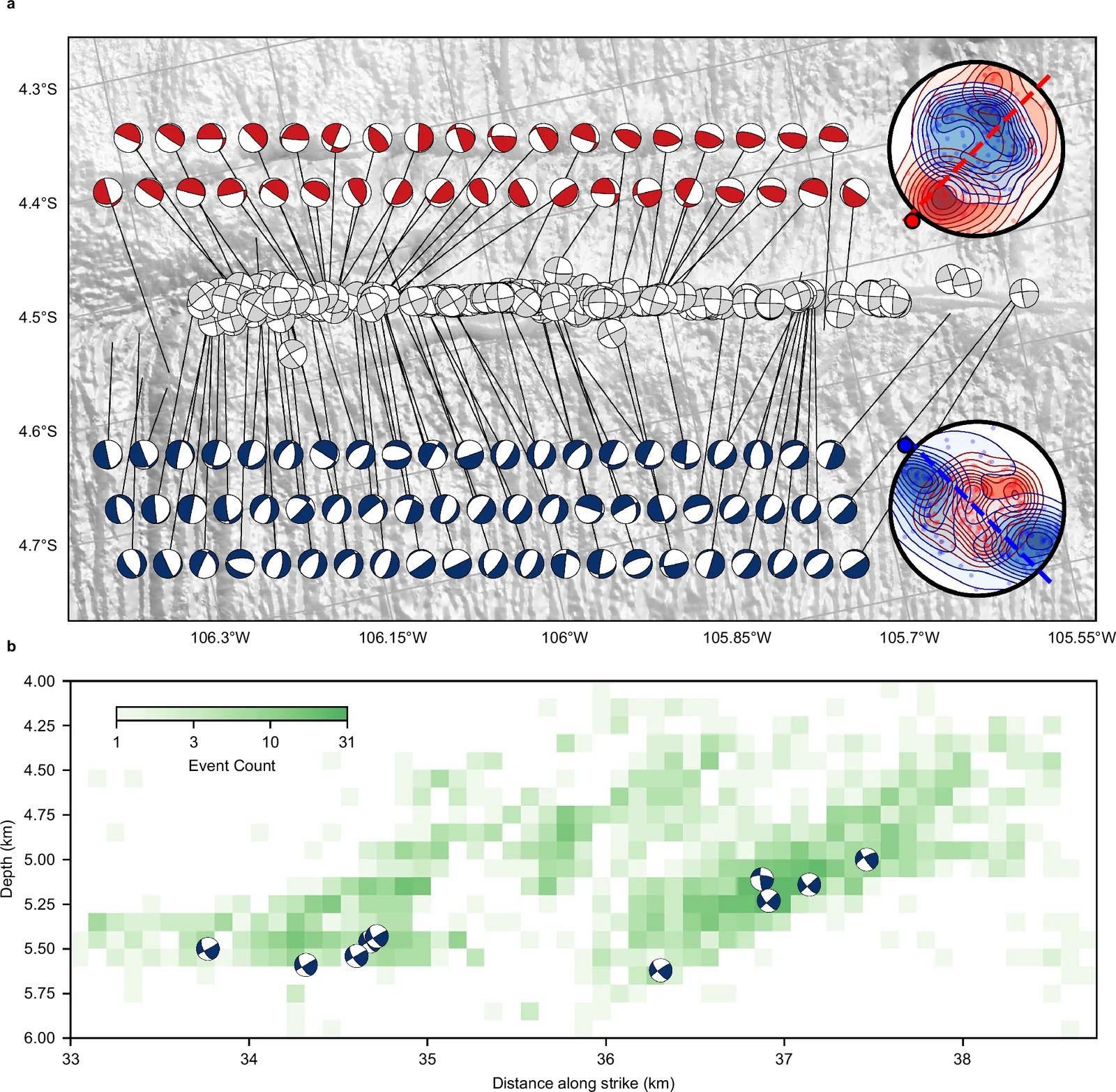
Diverse faulting types along the Gofar G3 segment. **a** Focal mechanisms for *M *≥ 1.3 events classified as normal, reverse, or strike-slip, colored by faulting type. The background shows seafloor bathymetry. Top inset, Compressional (P) and tensional (T) axis distributions for reverse events (red and blue dots) with kernel density contours. The red dashed line and large red dot show the modeled and inverted maximum principal stress (*σ*_1_) directions, respectively. Bottom inset, P- and T-axis distributions for normal events in the same format. The blue dashed line and large blue dot show the modeled and inverted minimum principal stress (*σ*_3_) directions, respectively. **b** Zoomed-in cross section (rectangle in Fig. 1d) showing normal-faulting focal mechanisms in side view, with seismicity density in the background.

Using the resulting focal mechanisms, we classify the earthquakes into seven faulting types, including normal (N), normal-strike-slip (N-SS), strike-slip-normal (SS-N), strike-slip (SS), strike-slip-reverse (SS-R), reverse-strike-slip (R-SS), and reverse (R), based on the plunge of their pressure (P), tension (T), and null (B) axes^42^ (Fig. 1d). Generally, 31% of all events are pure strike-slip earthquakes, whereas oblique-normal (N-SS and SS-N) and oblique-reverse (SS-R and R-SS) mechanisms make up 60% of the solutions. Additionally, 5% and 4% of events are composed of normal and reverse mechanisms, respectively, indicating that both extensional and compressional slip are present in the region. These diverse focal mechanisms occur broadly within a ~4-km-wide (strike-normal) zone along the fault (Figs. 1d and 2a).

We perform a stress inversion^43^ using our focal mechanism catalog and find that the maximum (*σ*_1_) and minimum (*σ*_3_) principal stress directions are horizontal, and are nearly uniform across the entire region (Figs. 1c, inset, and S3). The stress ratio ${R}_{{{\rm{ratio}}}}=\frac{{\sigma }_{2}-{\sigma }_{3}}{{\sigma }_{1}-{\sigma }_{3}}$ is about 0.54 for most fault patches (Fig. S3), with only slight variations (0.45–0.62). The ratio is close to 0.5, representing a typical strike-slip stress regime^44^. Stress inversions with subsets of data before and after the mainshock show consistent results (Figs. S4 and S5).

We find that the P-axes of reverse faulting (R) events closely align with the inverted *σ*_1_ direction, while the T-axes of normal faulting (N) events generally match the observed *σ*_3_ direction with a ~10° deviation (Fig. 2a). This observation suggests that the R and N events consistently have strikes ~40° and ~60° away from the strike of the G3 fault, respectively. Some of the N event mechanisms can be visually verified with seismicity alignments in along-strike cross sections. For example, a kilometer-scale seismicity cluster in Zone 3 at 5 km depth shows a westward-dipping feature that is consistent with the average dip of normal faulting events within the cluster (Fig. 2b). The agreement suggests a ~3-km-long normal fault with a strike of ~205° and a dip of ~30°, extending a few hundred meters along its strike across the G3 fault. By contrast, we do not observe distinct geometric features associated with R events, largely because other oblique-normal and oblique-reverse events coexist in the same areas. The diverse focal mechanisms indicate that smaller faults with a broad range of strike and slip directions are widespread and active at G3, despite the homogeneous stress regime in the region.

### Fault segmentation due to mechanical property variations

**Fig. 3:**
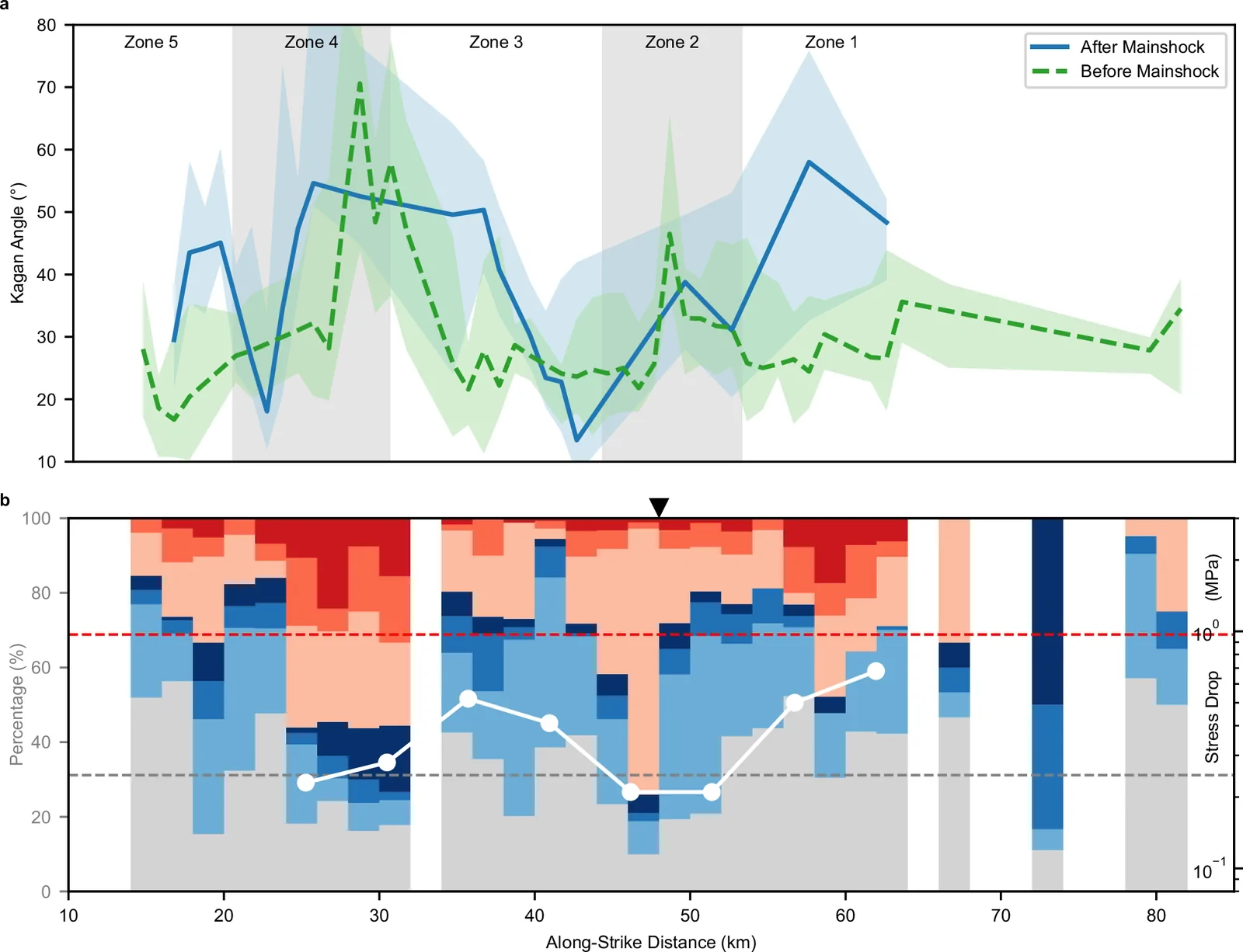
Faulting type varies systematically along strike. **a** Median Kagan angle relative to the 2008 M6 mainshock in each 1-km along-strike bin within the 4-km-wide fault zone, shown separately for pre- and post-mainshock periods. Shaded bands show the corresponding 25th–75th percentile ranges for each bin. **b** Faulting type percentage in 2-km along-strike bins. The colors represent faulting types as in Fig. 1. Only bins with at least 10 solutions are shown. The red and gray dashed lines mark the average percentages of events other than reverse and oblique-reverse events, and pure strike-slip events, respectively. The white curve and dots show the average stress drop^19^. The black triangle marks an active-source survey line^16^.

The faulting type distribution varies along strike, with different segments favoring different mechanisms (Fig. 3b). This focal mechanism segmentation correlates with the seismicity segmentation that divides the G3 fault into five zones. In Zones 1, 3 and 5, the SS events are most common, whereas in Zones 2 and 4, the reverse and oblique-reverse events predominate. We quantify this along-strike variation by comparing each microearthquake focal mechanism with the 2008 M6 mainshock^45^, computing the Kagan angle between each pair^46^ (Fig. 3a). The Kagan angle measures the three-dimensional rotation required to align two focal mechanisms, with smaller values indicating higher consistency (see “Methods”). The spatial pattern of the Kagan angles mirrors the segmentation identified from the spatiotemporal evolution of the G3 seismicity in 2008^5,14^. Before the mainshock, the median Kagan angle is 25° within the 2008 M6 mainshock rupture zone and 30° and 40° in the neighboring Zone 2 and Zone 4, respectively. This contrast shows that earthquakes within the mainshock zone share a more similar geometry and slip direction with the mainshock, while earthquakes in the adjacent barrier zones deviate from the mainshock focal mechanism. Similarly, the along-strike variation of the Kagan angle and faulting types are mutually consistent (Fig. 3a, b). In segments with more SS events (Zones 1, 3 and 5), the Kagan angle is generally smaller, while in zones where R, R-SS and SS-R events are more common (Zone 2 and Zone 4), the Kagan angle is larger. This correspondence is expected given the pure SS mainshock mechanism (strike = 100°, dip = 79°, rake = 3°^45^).

The along-strike variations in earthquake faulting type likely represent differences in fault mechanical properties between segments, which act to control along-strike fault segmentation and the associated dominant seismic and aseismic slip behavior. Under a regional stress regime with horizontal maximum and minimum principal stresses, strike-slip earthquakes are expected to be the most common^47^. Based on the Mohr-Coulomb failure criterion^48,49^, faults with other orientations can also slip at G3 under the same stress regime (see “Methods”). In a suite of synthetic earthquakes generated on the observed fault planes of the 2323 focal mechanisms, the relative proportions of faulting types are governed by mechanical parameters such as pore-fluid pressure and the internal frictional coefficient (see “Methods”). Specifically, when all other variables are held constant for this suite of synthetic events, the proportion of SS events decreases monotonically with rising pore-fluid pressure and/or decreasing frictional coefficient (Fig. S14). Therefore, a fault segment with a low proportion of SS events likely corresponds to a mechanically weak fault.

Assuming G3 has a uniform internal friction coefficient, the relative abundance of non-SS earthquakes implies elevated pore-fluid pressure in these segments. Thus, Zones 2 and 4 (Fig. 3b) with fewer SS events could be weak fault segments due to elevated pore-fluid pressure. A controlled-source electromagnetic survey at Zone 2 found a subvertical conductor extending into the lower crust^35^, indicating deep fluid penetration and supporting our explanation. These mechanically weak segments are more prone to stable aseismic slip and are less capable of accumulating significant stress^18,50^. By contrast, more SS focal mechanisms in Zones 3 and 1 suggest strong, locked fault patches capable of sustaining stress and generating large earthquakes, with lower pore-fluid pressure. This contrast in fault mechanical properties can explain a series of other observed variations at G3, including lower stress drops (Fig. 3b)^19^, lower seismic velocities^16,17,51^, and higher in situ Vp/Vs measurements^20^ in the barrier zone. These all suggest that the properties of the barrier zones are different from those of the mainshock zone and influenced by pore fluids^14^.

**Fig. 4:**
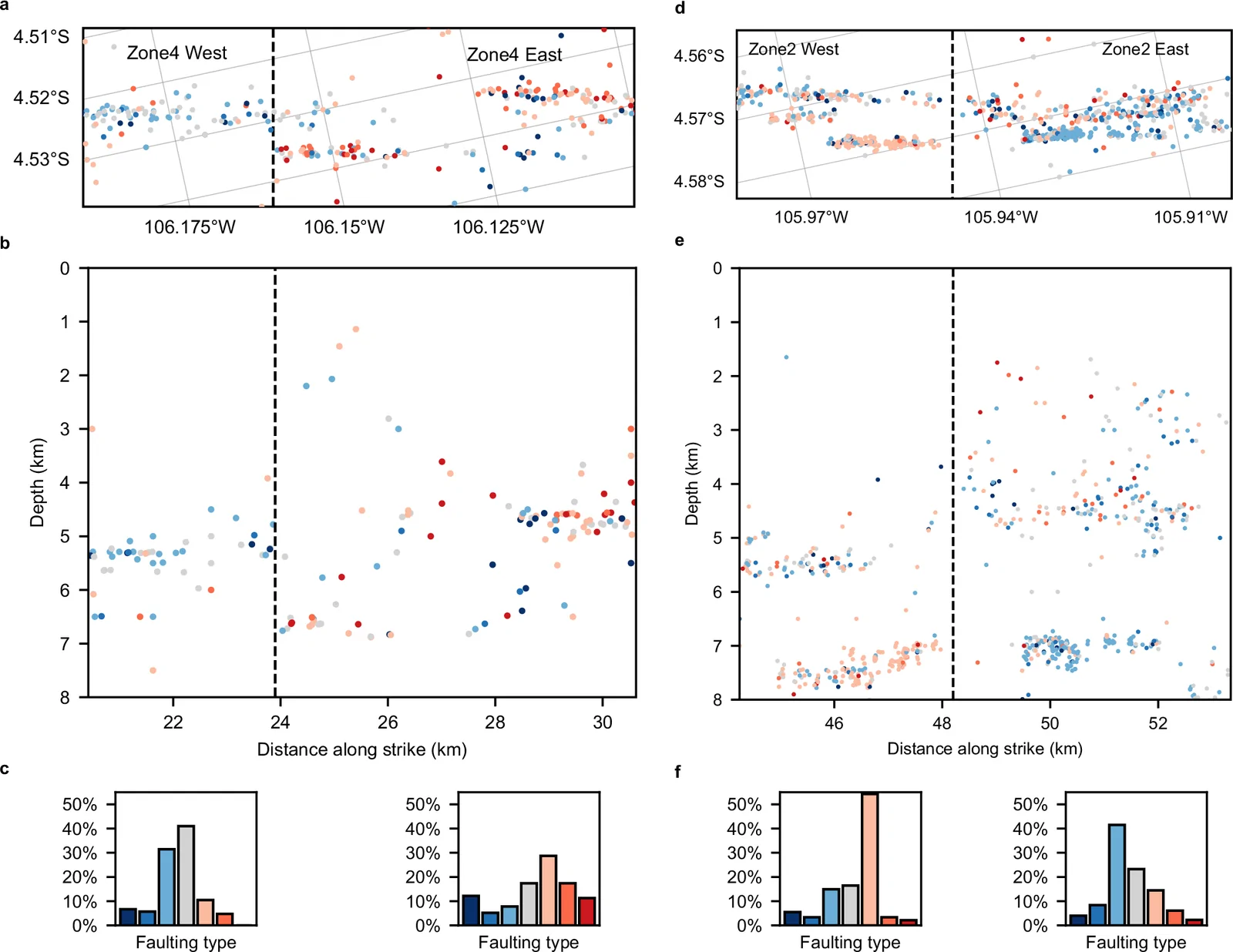
Faulting type distributions define subzones within the barrier zones. **a** Map view of subzones in Zone 4, with focal mechanisms colored by faulting type as in Fig. 1. **b** Cross section of the subzones in Zone 4. **c** Faulting type histograms for the subzones in Zone 4. **d–f** Subzones in Zone 2, shown in the same format as (**a–c**).

The barrier zones are dominated by different types of faulting (Fig. 4) with a heterogeneous distribution of oblique-slip earthquakes. This suggests that the barrier zones have more complex internal structures than the apparently simple segmentation pattern shown in the seismicity^5^. For example, faulting types in Zone 4 transition from being dominantly SS and SS-N in the west, to SS-R in the east, where Zone 4 connects with the M6 mainshock zone (Fig. 4a–c). Similarly, in Zone 2, the west and east fault patches exhibit distinct faulting types (Fig. 4d–f), with the west patch dominated by SS-R and the east patch dominated by SS-N. Our idealized simulation shows that assuming all other factors are constant, the proportion of R, R-SS, and SS-R events increases monotonically with rising pore-fluid pressure, whereas the proportion of N, N-SS and SS-N events peaks after reaching a hydrostatic state and slightly decreases afterwards (see “Methods” and Fig. S14). Therefore, the short 3–5 km segments within Zone 4 and Zone 2 that are dominated by reverse and oblique-reverse events (Figs. 3b and 4) likely represent the patches with highest pore-fluid pressures, which may be the weakest part of the entire G3 fault. This internal variation within the barrier zone is also observed by the conductivity contrast between the west and east patches of Zone 2^36^.

In addition, seismicity in Zone 2 shows two layers at depth with an aseismic zone at ~6–7 km depth (Fig. 4d–f). The focal mechanism type distribution in the shallow clusters only shows slight deviations from the overall pattern, whereas the deep clusters show more significant dominance (~60%) of SS-R and SS-N events for the west and east patches, respectively (Fig. S17). Seafloor fault traces show a compressional bend at the west patch of Zone 2 and an extensional bend at the east patch of Zone 2^38^, consistent with the fault architecture of the deep clusters. While the shallow clusters have the same trend of extension or compression as the deep clusters, respectively, they may be more fractured, as evidenced by the diverse faulting types (Fig. S17). Overall, our observations support a complex fault network in the rupture barrier zone as opposed to a simple planar configuration.

#### Temporal evolution of focal mechanisms indicates evolving fault zone conditions

The 2008 experiment captured the anticipated M6 earthquake at G3, which modulated seismicity of different fault segments in a variety of ways^5^. We compute the median Kagan angle between each microearthquake and the mainshock and find it increases after the mainshock in all zones (Fig. 3a). In Zones 1, 3, and 5, the pre- and post-mainshock Kagan angle distributions differ significantly (KS test; *P* < 0.05; Fig. S11)^52^. This increase is unlikely to be an artifact of uncertainty, as the focal mechanism uncertainty distributions show no significant change in Zones 3 and 5 and decreases in Zone 1 (Figs. S6–S10). This observation indicates that aftershock focal mechanisms in Zones 1, 3 and 5 differ more from the mainshock mechanism than those occurring before, suggesting an evolving fault zone condition.

**Fig. 5:**
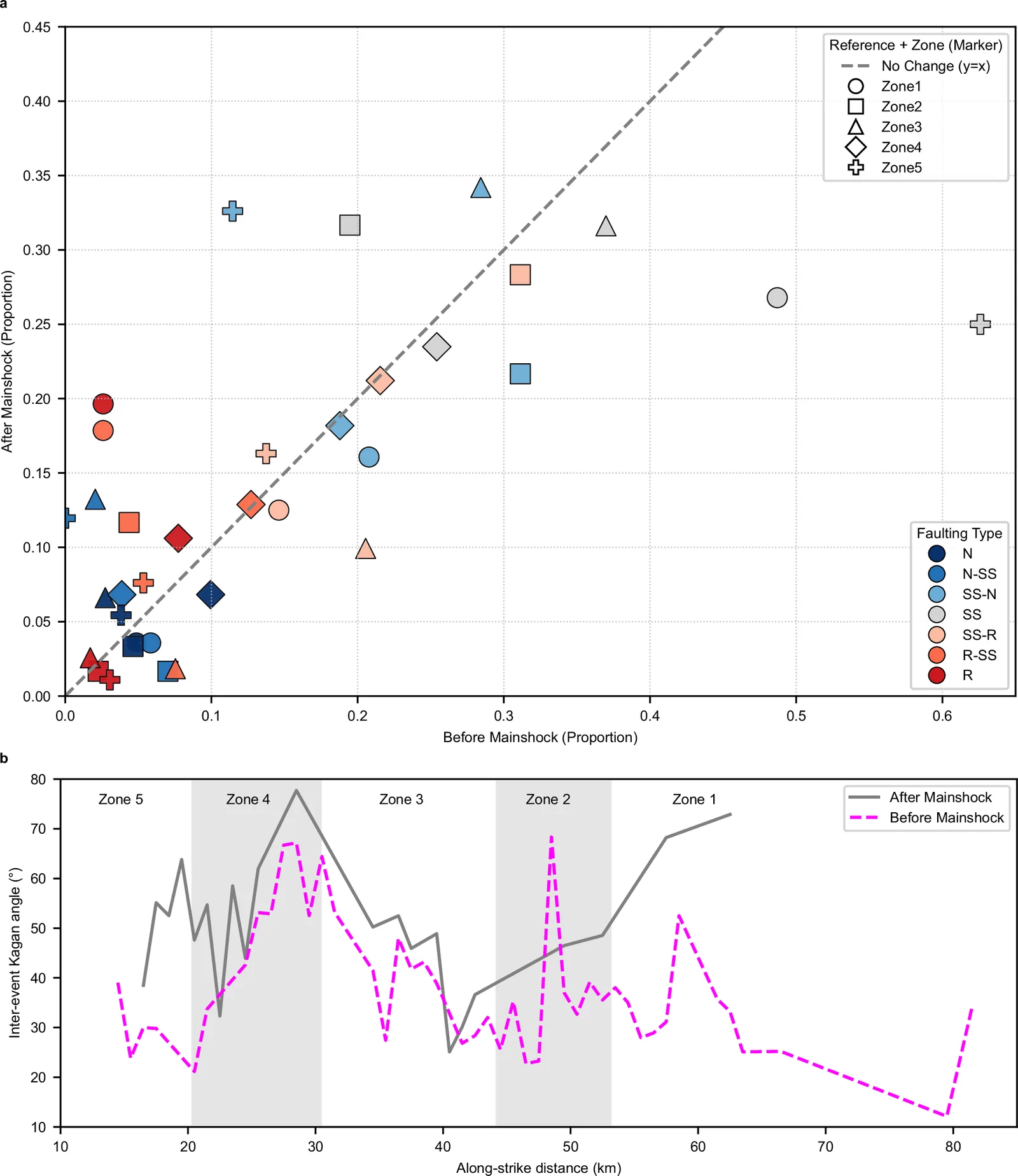
Faulting type and focal mechanism diversity evolve through the seismic cycle. **a** Faulting type proportion in each zone, evaluated for the pre- and post-mainshock periods. Each point represents one zone-faulting type pair; the marker shape identifies the zone, and the color identifies faulting type. The dashed 1:1 line indicates no change between periods. **b** Median inter-event Kagan angle in 1-km along-strike bins, shown separately for the pre- and post-mainshock periods. Abbreviations: N normal; N-SS normal-strike-slip; SS-N strike-slip-normal; SS strike-slip; SS-R strike-slip-reverse; R-SS reverse-strike-slip; R reverse.

We examine post-mainshock changes in faulting types across all zones and find that the percentage of SS events decreases significantly after the mainshock in Zones 1, 3, and 5 (Fig. 5a). The changes in the faulting type distributions are significant based on the *χ*^2^ test (see “Methods”). This reduction in SS events may partly contribute to the observed increase in Kagan angles. Zone 1 shows an increased proportion of reverse and oblique-reverse events, whereas Zones 3 and 5 are dominated by SS-N mechanisms after the mainshock.

Diverse aftershock focal mechanisms indicate that faults with various orientations are active, and have been linked to static stress perturbations^53^ and/or fault strength reduction due to coseismic rupture^54^. We quantify focal mechanism diversity using the median inter-event angular difference (see “Methods”), computed from all focal mechanism pairs^23^ in a region before and after the 2008 mainshock (Fig. 5b). We find a statistically significant (*P* < 0.05) increase in diversity in all zones (Figs. 5b and S11), indicating more faults with varying configurations are activated after the mainshock. This pattern is consistent with the increase in Kagan angles with respect to the mainshock in each zone.

The increased diversity could be due to stress perturbations from the mainshock, which promote failure on previously less optimally oriented subsidiary faults^53^. Alternatively, a fault strength reduction caused by the mainshock^54^ could also explain the increase in focal mechanism diversity. Coseismic rupture can form permeable pathways, leading to a pore-fluid pressure increase, and eventually weakening the fault^55^.

During the week preceding the 2008 M6 mainshock (days 254–261), the barrier zone (Zone 2) experienced an intense foreshock sequence of more than 20,000 events^14^. In the same period, a transient reduction of ~3% in S wave velocity was detected in Zone 2, implying a temporary change in the fault zone environment^14^. This velocity decrease is consistent with a fluid-mediated response to a stress perturbation, possibly induced by localized creep within the damage zone^14^. Our focal mechanism catalog shows that the proportion of pure SS events increased during this period in both the western and eastern parts of this zone, whereas the previously dominant oblique mechanisms (SS-R in the west and SS-N in the east) became less common (Fig. S13). The faulting type distribution change is statistically significant when considering the entire zone or the western part (*P* < 0.05, see “Methods”). This observation of elevated activity on the main SS fault during the period of the highest seismicity rate and largest velocity change supports models in which transient aseismic slip drives seismic swarms on OTFs^18,56^.

## Discussion

### Mechanical controls on fault segmentation and slip behavior

The faulting type distribution varies along strike despite a nearly uniform regional stress field. The stress inversion yields consistent principal stress orientations and a stress ratio close to 0.5 across the entire G3 strand (Fig. S3), yet the proportion of SS events drops in the barrier zones while reverse and oblique-reverse events increase (Fig. 3b). Because the homogeneous stress field cannot explain this variation, the along-strike differences in faulting type proportions instead reflect differences in the fault zones and the mechanical conditions. Coulomb failure modeling confirms that under the observed uniform stress regime, the proportion of non-strike-slip events that can be activated on the observed fault planes increases monotonically with rising pore-fluid pressure (Fig. S14). Segments with depleted SS populations therefore correspond to mechanically weaker fault zones with higher pore-fluid pressures. At G3, this identifies the barrier zones (Zones 2 and 4) as the weakest portions of the fault and the rupture zones (Zones 1 and 3) as the strongest, as suggested by other geophysical images^16,17,20,35,51^. We show that the along-strike heterogeneity in faulting type is an expression of heterogeneity in fault zone mechanical properties, primarily pore-fluid pressure^36^.

This mechanical segmentation offers a physical explanation for why barrier zones persistently terminate ruptures. At Gofar, large earthquakes have repeatedly nucleated near Zone 2 yet have never ruptured through it over the observed seismic cycles^2,6,14^. Our focal mechanism catalog shows that the 2008 M6 rupture zone (Zone 3) is bounded by kilometer-scale segments with dominant reverse and oblique-reverse faulting types on both sides, representing patches with the highest pore-fluid pressure (Figs. 3 and 4). This spatial pattern supports a model in which elevated pore-fluid pressure in the barrier zones reduces effective normal stress, promoting aseismic slip and inhibiting the stress accumulation required for large earthquake rupture during the interseismic period^57^. During coseismic rupture, dilatancy strengthening reduces pore-pressure in the barrier zone and increases effective normal stress to arrest large earthquake rupture^18^. The barrier zones thus function not merely as geometric discontinuities but as mechanically distinct domains whose properties are governed by fluid conditions and exert primary controls on the rupture of adjacent M6 earthquakes.

These mechanical distinctions have implications for the overall seismic behavior of OTFs. OTFs are commonly characterized as mechanically weak plate boundaries because they exhibit low seismic coupling^2–4,58^ and low rigidity^59^. At G3, however, the maximum horizontal stress is oriented ~45° to the transform fault, implying a comparatively strong fault^48,60^, in contrast with the San Andreas fault, where the maximum horizontal stress is oriented ~80° from the fault trace^61,62^. Our faulting type analysis resolves this apparent paradox: G3 is not uniformly weak but instead comprises strong, locked rupture patches bounded by weak barrier zones. Because the weak barriers occupy most of the cumulative fault length, the fault as a whole appears weakly coupled. If a similar patchwork of strong and weak segments characterizes other OTFs, this heterogeneity would explain both their globally observed weakness and low frictional coupling^4,58^ and their capacity to produce quasi-periodic characteristic earthquakes on the strong patches^1^.

The temporal evolution of faulting types through the seismic cycle further demonstrates the sensitivity of the fault network to perturbations. The post-mainshock increase in focal mechanism diversity across all zones indicates that many subsidiary faults with non-optimal orientations sit close to failure and are activated once the stress field is perturbed. This observation is consistent with the concept of a critically stressed crust^63,64^, in which faults of diverse orientations reside near their frictional failure threshold and can be reactivated by modest stress changes. In the OTF context, the pervasive near-critical state of the fault network, together with creep transients that may be more readily generated in the barrier zone due to fault zone fluids^65^, may explain an order of magnitude more foreshocks at OTFs than continental strike-slip faults^66^. These stress perturbations can cascade through the fault network^14^, generating the characteristic foreshock swarms observed before large OTF earthquakes^14,56^. The shift toward more SS events during the Zone 2 foreshock sequence (Fig. S13) may reflect that transient aseismic slip on the main fault preferentially loads the optimally oriented SS structures, temporarily reorganizing the faulting type distribution before the mainshock.

These insights are enabled by the catalog size of 2323 focal mechanisms. Prior OTF focal mechanism datasets, typically comprising a few to <200 mechanisms per transform^9,10,12,24–34,67–69^ (Table S1), have been instrumental in documenting diverse faulting types and revealing broad tectonic features such as differences across transform segments, ridge-transform intersections, mid-ocean ridges, and pull-apart basins. However, catalogs of <200 focal mechanisms generally provide limited resolution for resolving kilometer-scale statistical patterns in faulting type distributions along strike, linking those patterns to mechanical properties, or detecting temporal shifts through the seismic cycle. Therefore, the physical causes of ubiquitously observed segmentation along OTFs remain incompletely constrained^2^. The order-of-magnitude increase in catalog size (2323 events) allows statistical focal mechanism analysis to serve as a quantitative probe of fault zone mechanics.

### Comparison with other oceanic transform faults

**Fig. 6:**
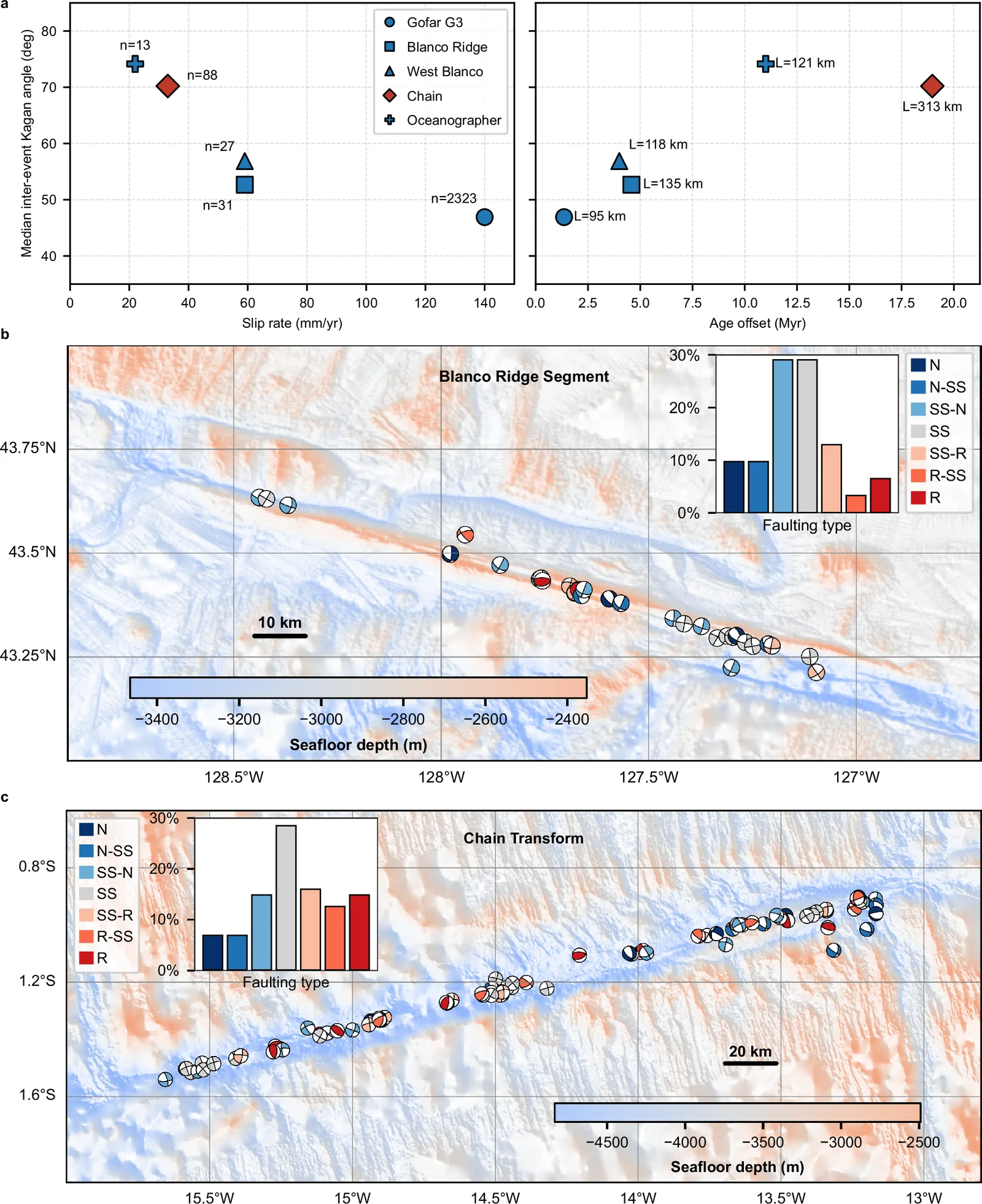
Comparison of faulting type segmentation and focal mechanism diversity with other oceanic transform faults (OTFs). **a** Focal mechanism diversity versus slip rate and age offset^2^ of five OTF segments^9,32,33^. Red and blue markers represent transpressional and transtensional OTFs, respectively^9,26,33,70^. Number of focal mechanism solutions (n) and fault length (L)^3^ are written beside each marker. **b** Focal mechanisms on the Blanco Ridge segment of the Blanco transform^32^, classified using the same method as in this study. The mechanisms on the mid-ocean ridge and within the pull-apart basin are excluded. **c** Focal mechanisms at the Chain transform fault^11,33^, classified using the same method. The mechanisms on the mid-ocean ridges are excluded. At both faults, pure strike-slip events account for ~30% of the catalog, with a range of other mechanism types also present. Abbreviations: N normal; N-SS normal-strike-slip; SS-N strike-slip-normal; SS strike-slip; SS-R strike-slip-reverse; R-SS reverse-strike-slip; R reverse.

Among OBS-based studies of OTFs, focal mechanism catalogs for small earthquakes (mostly *M* < 4) with more than 10 events are available for only a few systems, including Gofar from this study and previous catalogs at Blanco^32^, Chain^33^, and Oceanographer^9^ transform faults (Table S1). These OTFs sample different tectonic environments and slip rates from fast-slipping (Gofar, ~140 mm/yr), to intermediate-slipping (Blanco, ~59 mm/yr), to slow-slipping (Chain, ~33 mm/yr; Oceanographer, ~22 mm/yr)^2,37^. We therefore calculate the overall focal mechanism diversity, indicated by the median inter-event Kagan angle (see “Methods”) and compare this metric with the corresponding OTF slip rates. The median inter-event Kagan angle captures a first-order feature of each catalog, although it does not resolve along-strike detail. Overall, focal mechanism diversity appears inversely related to slip rate and positively related to age offset (Fig. 6a), regardless of fault length or tectonic stress regime, whether transtensional, as at Oceanographer^9^, or transpressional, as at Chain^33^. This dependence motivates the hypothesis that the controlling factors co-vary with slip rate and age offset, and the higher degree of focal mechanism diversity may reflect a higher degree of fault damage, greater fault zone heterogeneity, or enhanced fluid involvement. Such conditions may allow small earthquakes to occur on faults with varying geometries and faulting types, generating diverse focal mechanisms. However, given the limited number of OTFs studied, as well as differences in station coverage, catalog size, deployment duration, and magnitude range, the hypothesis remains to be tested with additional observations.

We further compare the faulting types among Gofar and two other OTFs that have the largest OBS-based focal mechanism catalogs, the Blanco Ridge segment of the Blanco transform^31,32^ and the Chain transform on the Mid-Atlantic Ridge^11,33^. For a consistent comparison, we classify mechanisms from Blanco and Chain using the same seven-type scheme^42^ (Fig. 6b, c). We note that this classification separates oblique mechanisms from pure SS, such that events reported as strike-slip under broader definitions are distributed among SS, SS-N, and SS-R categories in our scheme. Under this uniform classification, pure SS mechanisms account for ~30% of events on each OTF (Fig. 6b, c), while the proportions of other types differ among faults. Blanco and Gofar show SS-N mechanisms as the second most common type, consistent with transtension^26,70^. By contrast, Chain shows SS-R as the second most common type, and R-SS and R mechanisms are much more common than at Blanco and Gofar, consistent with a more transpressional regime^33^.

More importantly, both Blanco and Chain show along-strike variations in faulting type within their respective transforms. On the Blanco Ridge segment, compressional mechanisms cluster near longitude ~−127.7° (Fig. 6b), where the fault zone shows higher topographic and structural complexity and where prior work inferred a transition^26,71^ or a rupture barrier^7^ separating independent rupture groups of large earthquakes, observations that resemble the rupture barrier at Gofar. On the Chain transform, compressional mechanisms occur preferentially at restraining bends^33^. Though the smaller catalog sizes at the Blanco Ridge segment (31 mechanisms) and Chain (88 mechanisms) do not permit the same statistical analysis that we apply at Gofar, these along-strike patterns are qualitatively consistent with mechanical property variations being expressed in the spatial distribution of faulting types across a range of spreading rates. However, robust cross-scale comparisons require more catalogs that are comparable in both quantity and quality, highlighting the importance of additional data collection and systematic processing that resolve and compare both broad-scale and km-scale features across OTFs.

Along-strike segmentation of seismic and aseismic slip is not unique to OTFs; similar patterns have also been observed on continental strike-slip faults^72^, such as the San Andreas fault, where creeping zones are understood to be mechanically weak due to low shear strength of fault zone materials^73^, low surface roughness^74^, and/or elevated pore-fluid pressures^50^. These scenarios are consistent with our focal mechanism observations at G3. One difference is that on California strike-slip faults, geometrically simpler fault sections tend to creep aseismically^75^, whereas at Gofar the aseismic barrier zones have more diverse focal mechanisms and more complex subsidiary fracture networks in the bathymetry^38^ than the M6 mainshock zones (Fig. 5). This difference may reflect the distinct structural evolution of oceanic versus continental transform faults, and warrants further investigation^76^.

Using high-quality OBS data, we resolve 2323 microearthquake focal mechanisms at the westernmost Gofar transform fault, producing a catalog that is an order of magnitude larger than individual published OTF focal mechanism catalogs. We show that within a nearly homogeneous stress field, faulting types are segmented along strike, with the distribution of mechanism types coinciding with rupture zones and creeping barrier zones. Through Coulomb failure modeling, we establish that the barrier zones are mechanically weak, likely due to elevated pore-fluid pressure, providing a physical link between faulting types and the conditions that govern slip mode. The fault network is sensitive to perturbations through the seismic cycle. Comparisons among different transform faults suggest that focal mechanism diversity may be related to slip rate and age offset, and along-strike clustering of mechanism types may be common on OTFs. Given the systematic segmentation and regular earthquake behavior observed at OTFs worldwide, the mechanical controls on segmentation identified at Gofar may operate broadly, motivating future dense OBS deployments at other transform faults to test this hypothesis.

## Methods

### Data

We use data from 16 broadband ocean bottom seismometers from 2008 (network code ZD) and an earthquake catalog of 31,251 events from Gong and Fan (2022)^5^ to develop the focal mechanism catalog (Fig. 1). Because a large portion of that catalog does not have reported magnitudes, we recalculate the local magnitude for all events (see Text S4). Thirteen stations operated for most of the deployment with all three components available (Fig. S15). Three stations failed at different times during the deployment. Because the network comprised only 16 stations, we retained data from all stations while they were operational to maximize azimuthal coverage and the number of usable waveforms. When analyzing time-dependent variations, we explicitly account for potential changes in data quality and quantify associated uncertainties, and we use multiple statistical tests (see Statistical Tests) to assess the robustness of our interpretations.

### Focal mechanism inversion

For each event, we apply the local 1D velocity model used for developing the earthquake catalog^5,16^ to calculate a predicted P wave arrival time at each station. We window a waveform segment around the predicted arrival and re-pick a new P wave onset time within ±2 s of the predicted arrival. We use PickBlue-PhaseNet for re-picking, a deep learning model trained primarily on OBS data^39,77^. This step increases the number of valid P wave onsets available for subsequent processing (Text S1 and Fig. S19), and provides more accurate onset times^39^ which may improve first-motion polarity determination performance^40^. We then apply a deep-learning model^40^ to pick the polarity with input traces centered on the new PickBlue-PhaseNet onset times. We require a valid polarity to have a P phase probability ≥0.3 and a polarity probability ≥0.95. These joint thresholds result in a polarity consistency of 86% with manual picks (see Text S1 and Fig. S16). Any event with at least eight valid polarity picks and an azimuthal gap <180° is retained for focal mechanism inversion. Finally, the SKHASH program^41,78^ is used to obtain the focal mechanism solutions and associated fault plane uncertainties. The same 1D velocity model^16^ is used to compute the take-off angle and the full parameter settings are listed in Table S2. In total, we obtain focal mechanism solutions for 3055 events. We further apply a multi-parameter criterion to exclude potential outlier solutions based on the output metrics of the program^41,78^. For fault plane uncertainty (root-mean-square difference of all acceptable planes to the average fault plane), polarity misfit (weighted fraction of misfit polarities), and mechanism probability (percent of mechanisms close to the average mechanism), we analyze the distribution of each parameter and use the 95th-percentile value of each parameter as a cutoff. The resulting thresholds are: fault plane uncertainty ≤45°, polarity misfit ≤23%, and mechanism probability ≥0.38. Additionally, we require a station distribution ratio (how strongly the station geometry constrains the mechanism) ≥40%. For events with multiple focal mechanism solutions, we further require the probability difference between the preferred solution and the alternative solution(s) to be ≥0.1. With all these quality control criteria, the final catalog contains 2323 events (76% of the total 3055 solvable events) with magnitude of −0.3 to 4.4. Event examples are shown in Figs. S1 and S2. Excluding the mainshock, the original earthquake catalog contains three events with *M* > 4.4. These events are all located near the western boundary of the study area, west of longitude −106.2°, where poor azimuthal coverage leads to fault plane uncertainties that exceed our selection criterion. The average uncertainty for each of the five zones is in the range of 34.5°−37.6° before the mainshock and 35.2°−38.2° after the mainshock, with the greatest increase observed in Zone 2 (Figs. S6–S10). We conduct sensitivity tests regarding the quality control thresholds and find that the spatial patterns are largely consistent in a range of thresholds (see Text S3). However, given the 16-station network, stricter thresholds would not substantially improve individual solution quality (because the primary limitation is the network geometry) but would eliminate the sample size needed for along-strike analysis. We therefore rely on overall statistical patterns across hundreds of events rather than individual solutions to support our interpretation. In addition, we perform a test to confirm that the observed focal mechanism patterns cannot be explained by random polarity picks (see Text S2 and Fig. S18).

### Kagan angle calculation

The Kagan angle^46^ measures the three-dimensional rotation required to align two focal mechanisms. We use the Kagan angle between each microearthquake focal mechanism and the double-couple component of the M6 mainshock moment tensor^45^ to quantify the difference between each small earthquake and the mainshock. To obtain robust spatial patterns, we group earthquakes using 1-km along-strike bins, each spanning the full width of the fault zone in the strike-normal direction (4 km) and retain only bins containing at least 10 focal mechanism solutions in each time period (pre- and post-mainshock; Fig. 3a).

### Focal mechanism diversity

We quantify focal mechanism diversity by calculating the inter-event Kagan angle between all event pairs^23^ within a given spatiotemporal bin and summarizing each bin by the median value. We use 1-km along-strike bins, each spanning the full width of the fault zone in the strike-normal direction (4 km), and retain only bins containing at least 10 focal mechanism solutions in each time period (pre- and post-mainshock; Fig. 3a). We also perform the analysis for events within each of the five zones and compute inter-event Kagan angle for the pre- and post-mainshock periods, respectively (Fig. S12). This inter-event metric differs from the mainshock-referenced Kagan angle described above. High median Kagan angles relative to the mainshock indicate that the cluster mechanisms differ from the mainshock. If inter-event angles within the cluster remain low, this pattern may reflect a distinct, persistent faulting type. By contrast, high inter-event Kagan angles indicate that events within the cluster are mutually dissimilar, suggesting activation of multiple fault structures with different orientations and/or faulting types.

### Stress inversion

We invert the principal stress directions and their relative ratio within 2-km by 2-km subareas in the entire study region^43^. A grid node must have at least 10 focal mechanism solutions to qualify for the stress inversion. The inversion is performed for three time periods: the entire year of 2008, from January 1, 2008 until before the M6 mainshock (September 18, 2008), and from after the M6 mainshock to the end of the year. The results are shown in Figs. S3–S5.

### Statistical tests

We perform statistical tests to verify the spatiotemporal variation of focal mechanism uncertainties and faulting type distributions, and the temporal variation of the focal mechanism consistency with the mainshock and focal mechanism diversities in each zone before and after the 2008 M6 mainshock. We apply the Kolmogorov-Smirnov (KS) test^52^ to the fault plane uncertainty distributions in each zone before and after the mainshock. For Zones 3 and 5 (Figs. S8 and S10), the uncertainty distributions did not change significantly after the mainshock (*P* > 0.05). For Zones 2 and 4 (Figs. S7 and S9), the uncertainty increased significantly after the mainshock (*P* < 0.05). In Zone 1 (Fig. S6), the uncertainty decreased significantly after the mainshock (*P* < 0.05). We apply the KS test to the individual event–mainshock Kagan angle distribution in Zones 1, 3, and 5 before and after the mainshock to evaluate whether the focal mechanism consistency with that of the mainshock is significantly different. All three zones have *P* < 0.05, indicating the focal mechanisms became significantly more inconsistent with the mainshock (Fig. S11). We use a *χ*^2^ test to evaluate faulting type distributions in each zone before and after the mainshock. Only Zone 4 does not show a significant faulting type distribution change (*P* > 0.05). We also apply a *χ*^2^ test to evaluate the faulting type distribution within the west and east patches of Zone 2 during the foreshock period (days 254–261) with respect to the prior period (days 1–253). In the west patch, the faulting type distribution shows a significant difference (*P* < 0.05) between the two periods (Fig. S13). In the east patch, the difference is not significant (*P* > 0.05). Considering the entire Zone 2 together, the difference is still significant (*P* < 0.05). The same conclusion is obtained using a two-class classification (SS versus non-SS), indicating a significant increase in the fraction of SS events in Zone 2. We apply the KS test to the inter-event Kagan angle distribution in each zone before and after the mainshock to evaluate whether focal mechanism diversity changes significantly between the pre- and post-mainshock periods. The distributions show that focal mechanisms become significantly more diverse after the mainshock in all zones (*P* < 0.05; Fig. S12). This result is most robust in Zones 1, 3, and 5, where fault plane uncertainty does not increase significantly after the mainshock.

### Coulomb failure simulation

We perform a Coulomb failure simulation using the observed stress direction and ratio (*R*_ratio_ = 0.54) to explore the effect of different parameters to the focal mechanism type distributions. The failure criterion is described as *τ* = *c*_0_ + *μ*(*σ*_*n*_ − *p*_*f*_), where *τ* is the shear strength, *c*_0_ is cohesion, *μ* is the static friction coefficient, *σ*_*n*_ is normal stress, and *p*_*f*_ is pore-fluid pressure^48,49^. We set the maximum principal stress *σ*_1_ at 54° from north horizontally, the intermediate principal stress *σ*_2_ vertically, and the minimum principal stress *σ*_3_ perpendicular to the two, informed by the stress inversion results (Fig. S3). The absolute value of *σ*_2_ is assumed to be lithostatic, calculated at 5 km as 132 MPa, with an assumed density of 2700 kg/m^3^ and a gravitational acceleration of 9.8 m/s^2^. The assumed fault strikes and dips are taken from the focal mechanism solutions, and we randomly select one of the two fault planes. Other parameters include differential stress (*δσ *= 100–200 MPa), cohesion (*c*_0_ = 0–10 MPa), friction coefficient (*μ* = 0.35–0.85), and pore-fluid pressure (*p*_*f*_ = 30–120 MPa). For every simulation, we only evaluate one parameter, while keeping the remaining parameters the same as the reference parameter set, *δσ* = 130 MPa, *c*_0_ = 5 MPa, *μ* = 0.6, and *p*_*f*_ = 80 MPa. For each fault plane, we calculate the normal and shear stresses as follows:

1 $${\sigma }_{n}={{{\bf{n}}}}^{T}{{\boldsymbol{\sigma }}}{{\bf{n}}}$$

2 $${{\boldsymbol{\tau }}}={{\boldsymbol{\sigma }}}{{\bf{n}}}-{\sigma }_{n}{{\bf{n}}}$$

where ***σ*** is the input stress tensor and ${{\bf{n}}}$ is the unit normal vector of the fault plane. If the shear stress $|{{\boldsymbol{\tau }}}|$ exceeds the shear strength, we label the fault plane as permissible for failure, and save the maximum shear stress direction as the rake, yielding a synthetic focal mechanism. We classify all simulated focal mechanisms using the same method as in Section Diverse Focal Mechanisms in a Uniform Stress Regime. Fig. S14 shows how the percentage of each focal mechanism type changes with each variable.

## Supplementary information

Supplementary information

Dataset 1

Transparent Peer Review file

## Data Availability

The waveform data are available from the EarthScope Consortium under the network code ZD. The metadata of the network can be accessed at https://ds.iris.edu/mda/ZD/?starttime=2007-01-01T00:00:00&endtime=2009-12-31T23:59:59. The earthquake catalog is from Gong and Fan (2022)^5^ and is archived in Marine Geoscience Data System (https://doi.org/10.26022/IEDA/331024). The bathymetry data are obtained from https://www.ncei.noaa.gov/maps/autogrid/. The focal mechanism catalog is attached as Supplementary Data 1.
