# Supplementary material for: Focal mechanism variations reveal mechanical controls on segmentation at oceanic transform faults
Source: Nat Commun. 2026 Aug 15;17:9836. doi: 10.1038/s41467-026-76407-5 (PMC13578451; doi:10.1038/s41467-026-76407-5)
Supplement: Supplementary file 1 [file 41467_2026_76407_MOESM1_ESM.pdf]

Supplementary Materials for "Focal mechanism variations reveal mechanical controls on segmentation at oceanic transform faults"

Fengzhou Tan[1*], Wenyuan Fan[1], Peter M. Shearer[1], Mark D. Behn[2], Jeffrey J. McGuire[3]

[1*]Scripps Institution of Oceanography, University of California, San Diego, La Jolla, CA, USA.
[2]Department of Earth and Environmental Sciences, Boston College, Chestnut Hill, MA, USA.
[3]Department of Geology and Geophysics, Woods Hole Oceanographic Institution, Woods Hole, MA, USA.

*Corresponding author(s). E-mail(s): f4tan@ucsd.edu, fengzhou.tan@gmail.com;
Contributing authors: wenyuanfan@ucsd.edu; pshearer@ucsd.edu; behnm@bc.edu; jmcguire@whoi.edu;

This PDF file includes:

Supplementary Text S1 to S4
Figures S1 to S19
Tables S1 to S2

Supplementary Text

S1 Visual Inspection of Polarity Picks

We conduct a visual inspection to evaluate polarity picks from RPNet [1] and to justify our thresholds selection. The original earthquake catalog [2] used EQTransformer [3] for phase picking. EQTransformer was trained primarily on land-station data and produces less precise P-wave onset times in the ocean bottom seismometer (OBS) environment, where ambient noise characteristics and instrument responses differ from those of land stations. To improve the P-wave onset accuracy, which is critical for reliable first-motion polarity determination, we re-pick arrivals using PickBlue-PhaseNet [4], a deep-learning model trained on OBS data. This step increases the number of valid P-wave onsets available for subsequent processing. As exemplified by the event in Fig. S19, four of the stations with valid P-wave onset picks and polarities in our workflow have no corresponding P-wave onsets in the original picks of Gong and Fan (2022) [2]. These stations therefore would not enter the subsequent polarity-picking and focal-mechanism workflow without re-picking the P-wave onsets. Station G14 has a P-wave onset in the original picks of Gong and Fan (2022), but the polarity determined has a probability of < 0.95 and would therefore be excluded from the valid polarity set. Therefore, without re-picking the P-wave onsets, this event would have only seven valid polarity picks, and its focal mechanism would not be resolved. This example illustrates that re-picking the P-wave onsets can recover valid polarity constraints that would otherwise be missed and affect the subsequent analysis. However, we do not relocate the earthquakes using the new picks; the original hypocenter locations from Gong and Fan (2022) [2] are retained throughout this study.

For a random-sampling visual inspection, we select 100 events from the earthquake catalog [2] and use PickBlue [4] to pick P arrivals within ± 2 s of the predicted arrival time calculated from the same 1D velocity model used throughout this study [5]. Picks with phase probability ≥ 0.1 are retained at this step, yielding 598 traces with valid P picks. We then apply RPNet [1] to each trace to obtain the polarity pick and its associated probability. For the same 100 events and associated traces, we also manually classify the polarity of each trace into up, down, or unknown for comparison. Across a range of phase- and polarity-probability thresholds, we compute the fraction of automatic polarity picks that match manual labels. Because RPNet outputs only binary up/down labels, any trace manually labeled as unknown is counted as inconsistent whenever the automatic pick passes both thresholds. The result is summarized in Fig. S17.

Generally, the consistency increases with higher phase and polarity thresholds, indicating that increasing both probabilities help eliminate low-quality data and improve polarity accuracy. Similar levels of consistency can be achieved with different combinations of the two thresholds. Our preferred combination, phase probability of 0.3 and polarity probability of 0.95, yields about 86% consistency with manual picks. It is important to note that these two thresholds are applied jointly that a pick must satisfy both criteria simultaneously. The polarity probability threshold of 0.95 ensures that only high-confidence polarity determinations enter the inversion, so the effective

quality filter is considerably stricter than the phase-probability threshold of 0.3 alone would suggest.

We also evaluate the effect of adopting higher phase-probability thresholds. For example, raising the phase-probability threshold from 0.3 to 0.6 (while keeping the polarity-probability threshold at 0.95) increases the polarity consistency from 86% to 91%, but removes about 32% of the focal mechanism solutions from the catalog. By experimenting with a broad range of threshold combinations, we find that the along-strike segmentation of faulting types and the spatial patterns in Kagan angle remain qualitatively unchanged whenever the polarity consistency exceeds ∼80%. We therefore retain the 0.3/0.95 combination to balance the quantity and quality of the focal mechanism solutions, preserving the sampling size needed for statistical kilometer-scale along-strike analysis.

While this analysis provides an estimate of the performance of the automated polarity-picking workflow and supports our threshold choices, the exact number should not be interpreted as an absolute accuracy, because the sampling size is relatively small, and manual labels are subjective and may not represent the ground truth.

S2 Random Polarity Pick Test

We perform a test to confirm that the observed focal mechanism patterns cannot be explained by random polarity picks. This test is designed to assess whether the network geometry and station configuration alone could produce apparent spatial patterns in faulting types. Because the OBS network is not symmetric with respect to the fault and station availability changes through time (Fig. S15), it is important to verify that the observed along-strike segmentation is not an artifact of azimuthal coverage or station operation time. The random test preserves the exact network geometry and event locations but removes all true polarity information, providing a null hypothesis against which the real catalog can be compared.

We use the same set of events as in the real dataset and retain the same station configuration for each event, but replace each polarity pick with a random binomial up/down assignment. We then apply the same SKHASH inversion and quality control criteria, obtaining 1,720 events with focal mechanism solutions. We classify these events into seven faulting types with the same method [6] and calculate the Kagan angle [7] with respect to the mainshock. The results are shown in Fig. S18.

The random-polarity catalog differs from the real catalog in several diagnostic ways. Strike-slip events do not show consistent left-lateral motion along strike (Fig. S18a), in contrast to the local tectonic setting and the coherent left-lateral mechanisms seen in the real catalog. The P-axis distribution of R events and the T-axis distribution of N events are scattered and do not agree with the inverted or modeled directions (Fig. S18a), whereas the real catalog shows tight clustering aligned with σ_1 and σ_3. Further, Kagan angles are much larger than those in Fig. 3 (median $\sim 80^\circ$ versus $\sim 30^\circ$ in the real catalog) and show no coherent spatial pattern comparable to the observations (Fig. S18b). The faulting type distribution shows little along-strike variation and does not exhibit the segmentation observed in the real catalog (Fig. S18c). In particular, the proportion of N and R events is approximately 20% each in the random test, while SS events account for less than 10%, reflecting the geometry of the

classification scheme rather than any tectonic signal. These results confirm that the observed focal mechanism patterns, including the along-strike segmentation of faulting types, the consistency of P- and T-axes with the regional stress field, and the spatial variation of Kagan angles, are not artifacts of the automated polarity-picking procedure or the network geometry.

S3 Sensitivity of Spatial Patterns to Quality Control Thresholds

To evaluate the robustness of the observed along-strike patterns to the choice of quality control thresholds, we repeat the analysis using stricter cutoffs. Our preferred thresholds are based on the 95th-percentile values of the fault plane uncertainty, polarity misfit, and mechanism probability distributions, yielding 2,323 events. Using the 90th-percentile values instead yields 2,032 events (67% of the 3,055 solvable events), and using the 80th-percentile values yields 1,652 events (54%). Under both stricter threshold sets, the overall faulting-type distribution and the along-strike spatial patterns remain largely consistent with those obtained from the preferred catalog. In particular, the depletion of SS events in the barrier zones (Zones 2 and 4) and the enrichment of reverse and oblique-reverse mechanisms in these same zones persist across all threshold choices. Therefore, the spatial patterns identified in this study are robust to reasonable variations in quality control criteria and are not driven by marginal-quality solutions near the cutoff boundaries.

S4 Magnitude Calculation

The published 2008 earthquake catalog contains 31,251 events, of which only 6,395 have reported magnitude values, with a magnitude of completeness of 0.6 [2]. In this study, we follow Fan et al. (2026) [8] and use the same parameters to calculate magnitudes, obtaining magnitude estimates for 30,092 events with a magnitude of completeness of 0.4. The difference likely arises from the Obspy filter applied to short time windows, the filter band, the window length, the signal-to-noise ratio, and the required number of stations. For the 6,338 events with magnitude estimates in both Gong and Fan (2022) [2] and this study, the two sets of estimates are in close agreement, with a mean difference of 0.03 (updated minus original).

Supplementary Figures

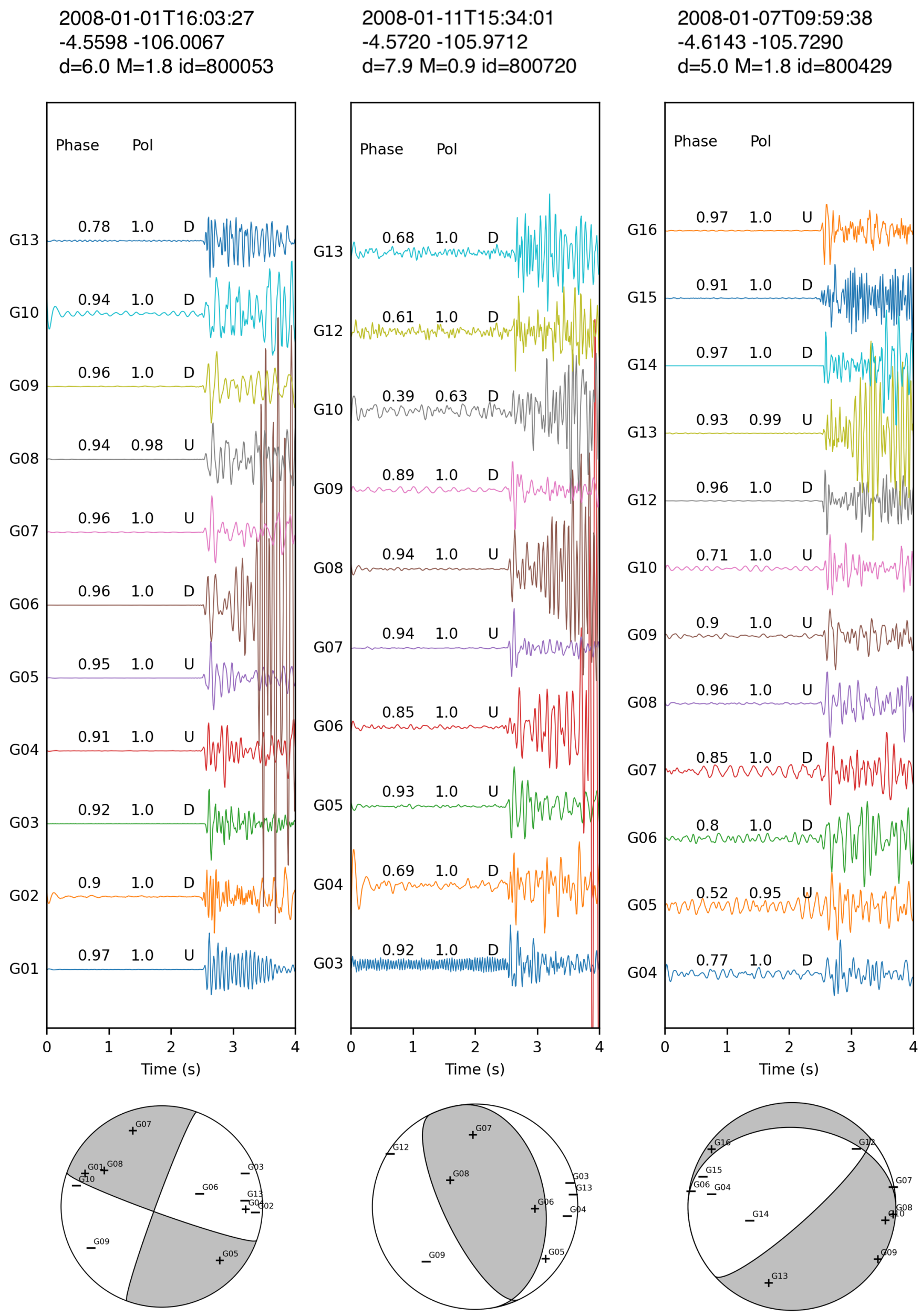

Fig. S1 Examples of P-wave polarity picks and associated focal mechanism solutions. High-pass filtered (5 Hz) waveforms from the vertical component are shown for each station. The "Phase" and "Pol" columns are probability of phase picking and polarity picking, respectively. The P-wave onset is picked by PickBlue-PhaseNet with a probability threshold of 0.3, aligned at 2.5 s. The polarity is picked by RPNet. "U" and "D" mark the picked polarity "up" and "down", respectively. Only polarities with probability $\geq$0.95 are used. The focal mechanisms and corresponding polarities from each station are shown in the bottom row of each panel. Three panels show examples of strike-slip, reverse, and normal earthquakes, respectively. Earthquake ID, origin time, magnitude and hypocenter location are provided in the title of each panel.

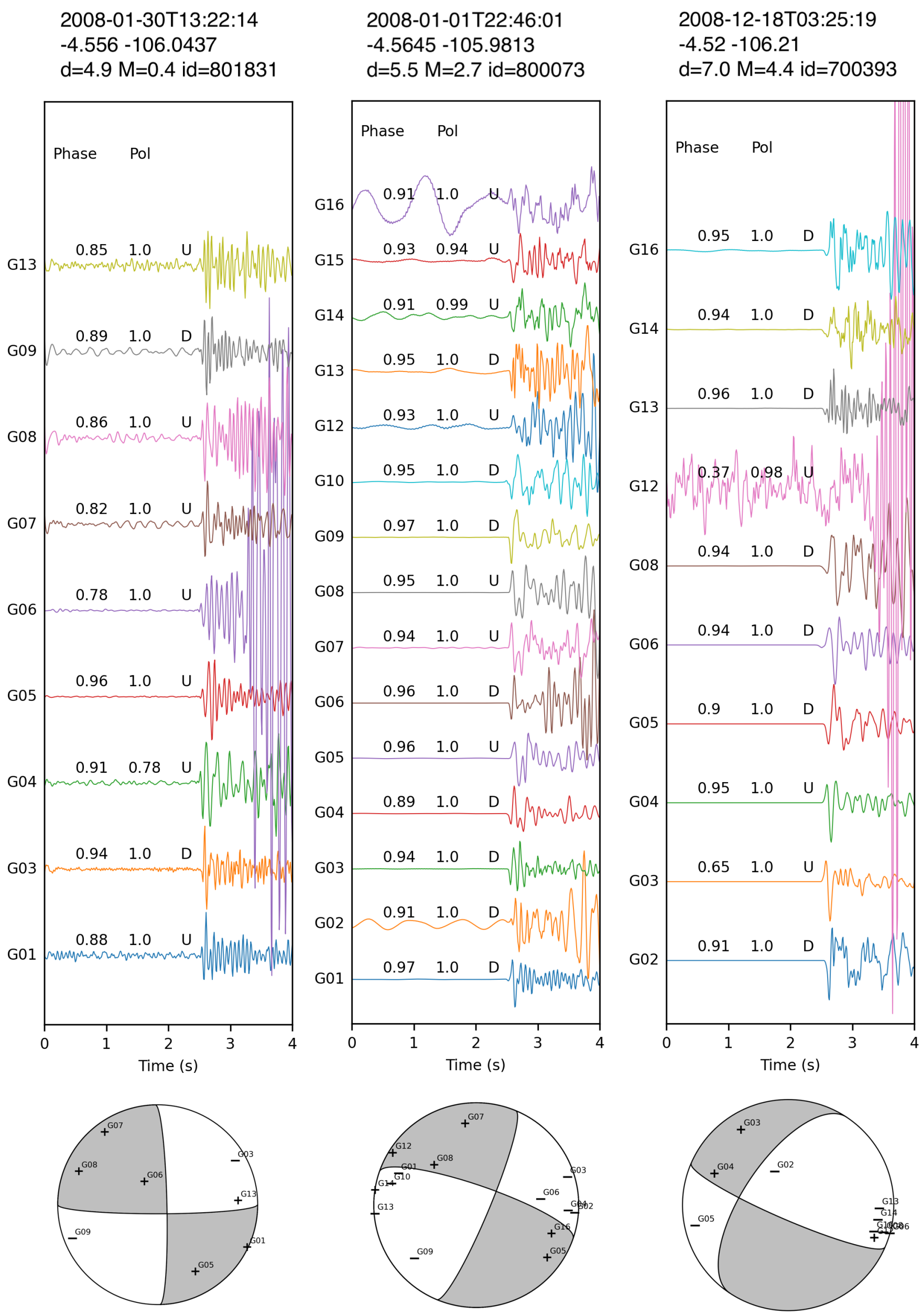

Fig. S2 Additional examples of P-wave polarity picks and associated focal mechanism solutions with a wider range of magnitudes. Formats are the same with Fig S1, except that the waveforms of the two larger events 800073 and 700393 are high-pass filtered at 1 Hz for better illustration of the polarities.

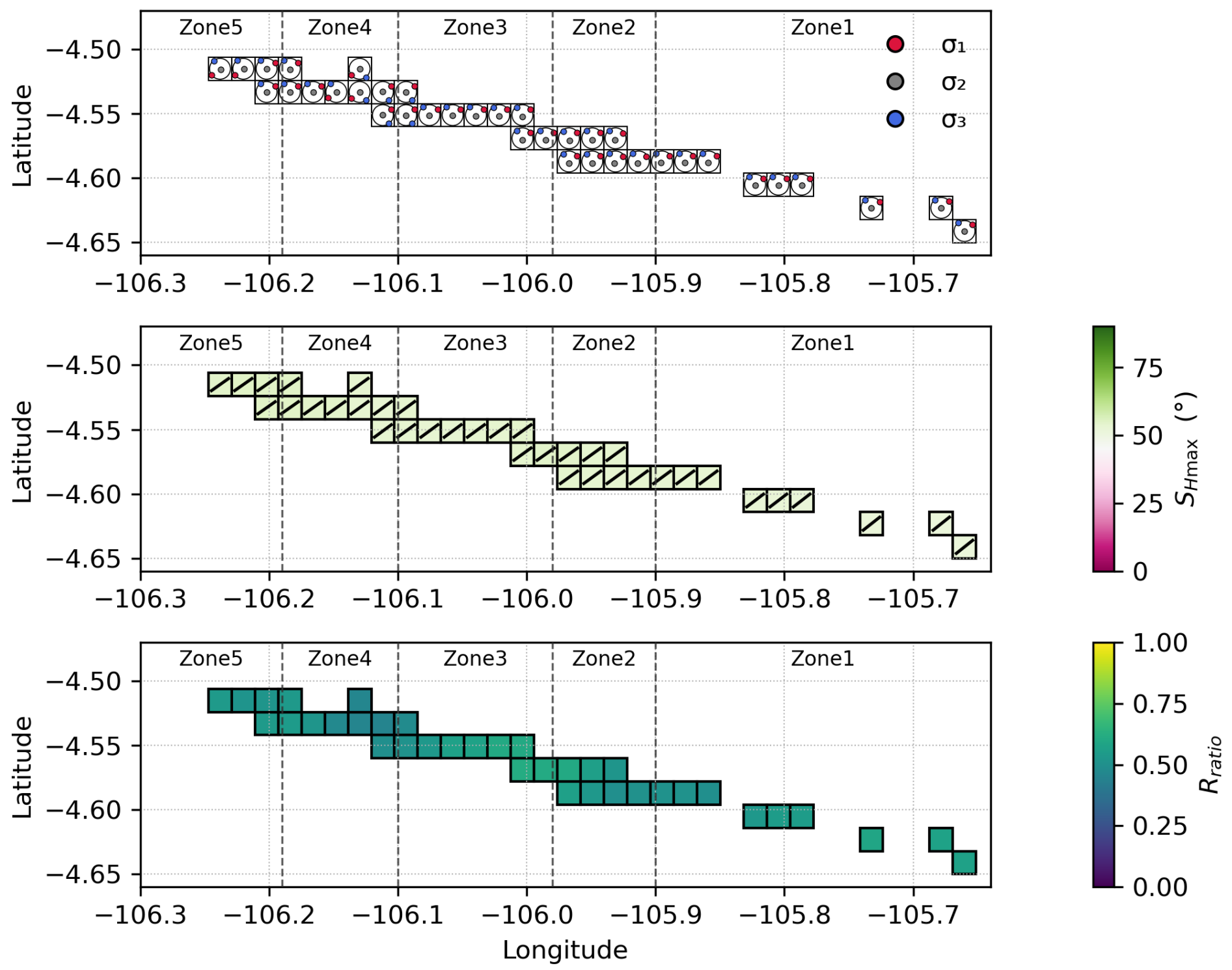

Fig. S3 Stress inversion results using all focal mechanism solutions from the entire 2008 period. Each square represents a 2-km by 2-km subarea; only subareas with at least 10 focal mechanism solutions are included. Top panel shows the three principal stress directions. Middle panel demonstrates the maximum horizontal stress direction shown as a line segment within each square. The background color indicating the maximum horizontal stress direction. The maximum horizontal stress is ranging from 51° to 55° clockwise from north. Bottom panel shows the stress ratio in each area, with values ranging from 0.45 to 0.62. Overall, the stress regime is uniform over the entire fault zone: σ_1 (maximum) and σ_3 (minimum) are horizontal, σ_2 (intermediate) is vertical, and the maximum horizontal stress makes an angle of ∼49° to the fault strike. The average stress ratio is 0.54, with only modest local deviations.

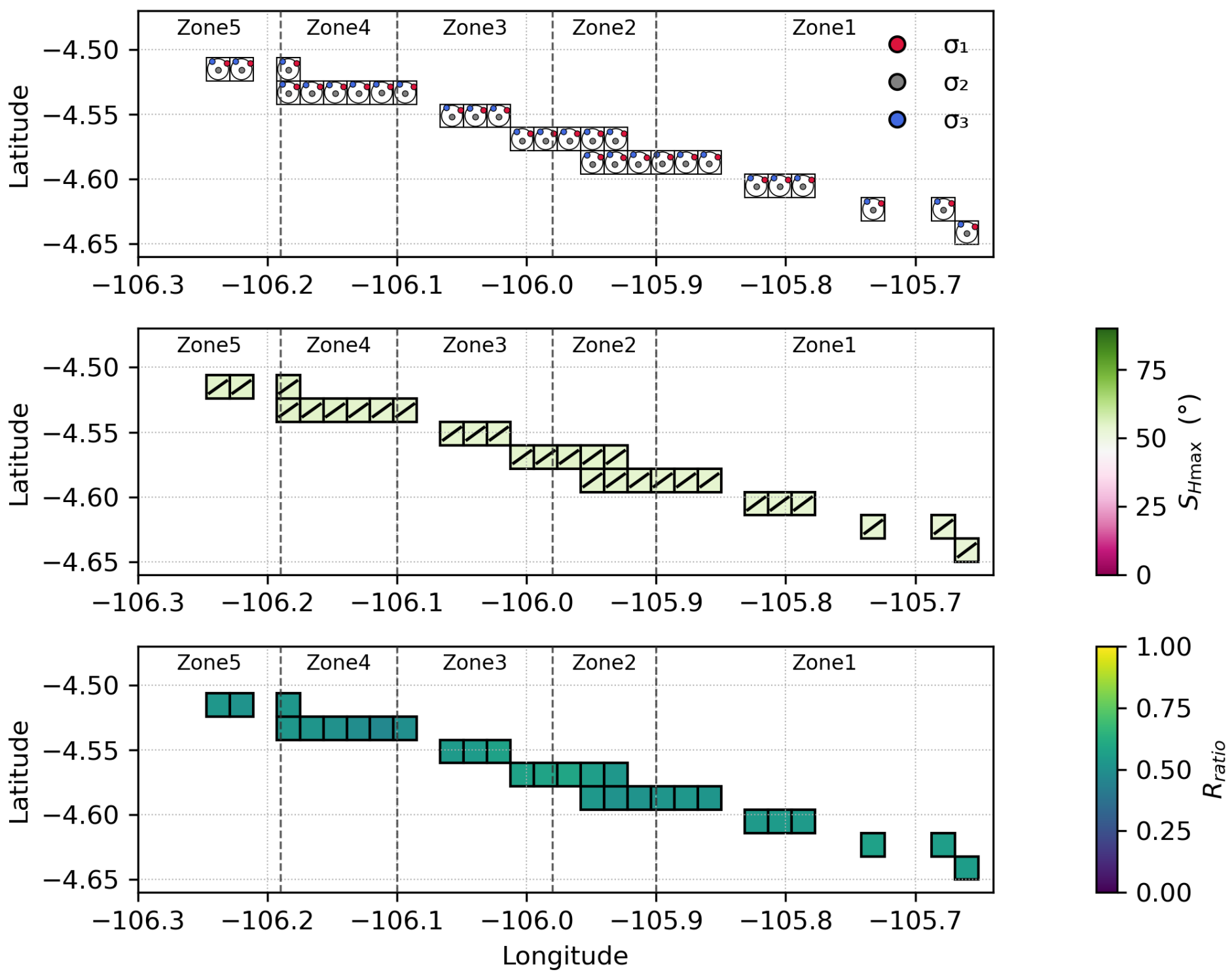

Fig. S4 Stress inversion results using focal mechanism solutions during the pre-mainshock period. Formats and legends are the same to those in Fig. S3. The stress regime is uniform and similar to Fig. S3. The maximum horizontal stress directions range from 53° to 55° clockwise from north. The average stress ratios range from 0.47 to 0.59 with an average of 0.53.

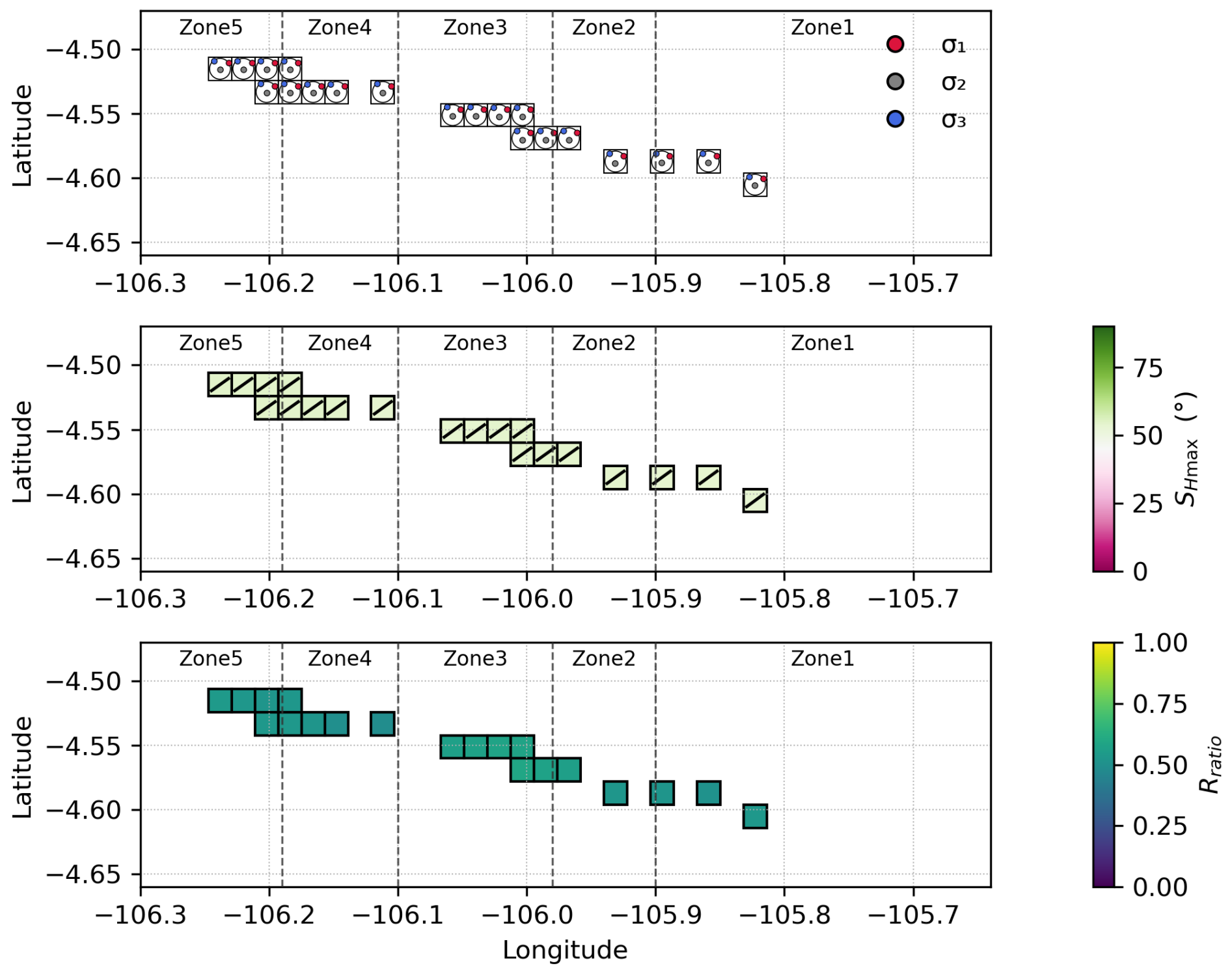

Fig. S5 Stress inversion results using focal mechanism solutions during the post-mainshock period. Formats and legends are the same to those in Fig. S3. The stress regime is uniform and similar to Fig. S3. The maximum horizontal stress directions range from 54° to 55° clockwise from north. The average stress ratios range from 0.49 to 0.59 with an average of 0.54.

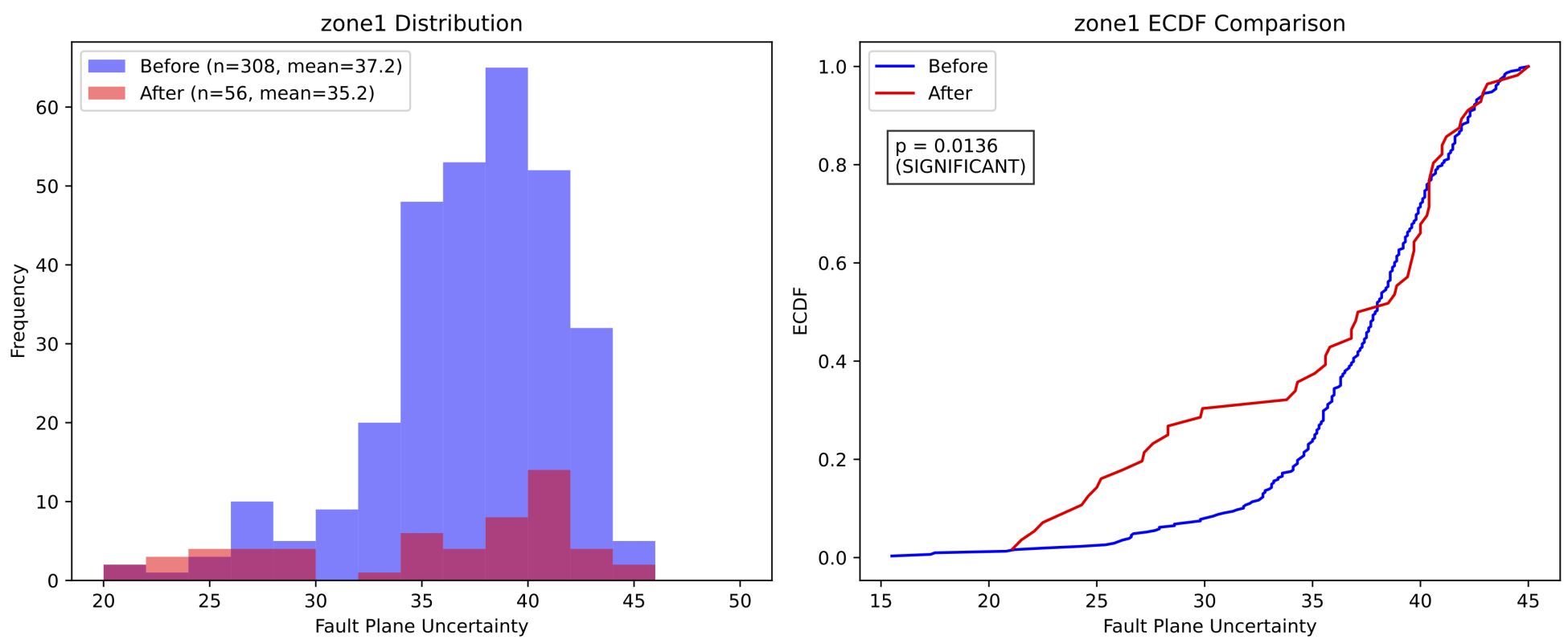

Fig. S6 Focal mechanism fault plane uncertainty for events in Zone 1. Left panel shows the histograms of the fault plane uncertainties before and after the mainshock. Average fault plane uncertainty is 37.2° and 35.2° for events occurring before and after the M6 mainshock, respectively. Right panel shows the empirical cumulative distribution functions (ECDF) of the event uncertainties before and after the mainshock. The comparison of the ECDFs helps visualize whether the fault plane uncertainty distribution changed after the mainshock. The p value is from the KS test; $p < 0.05$ indicates statistically significant difference between the fault plane uncertainty distributions before and after the mainshock (see Methods).

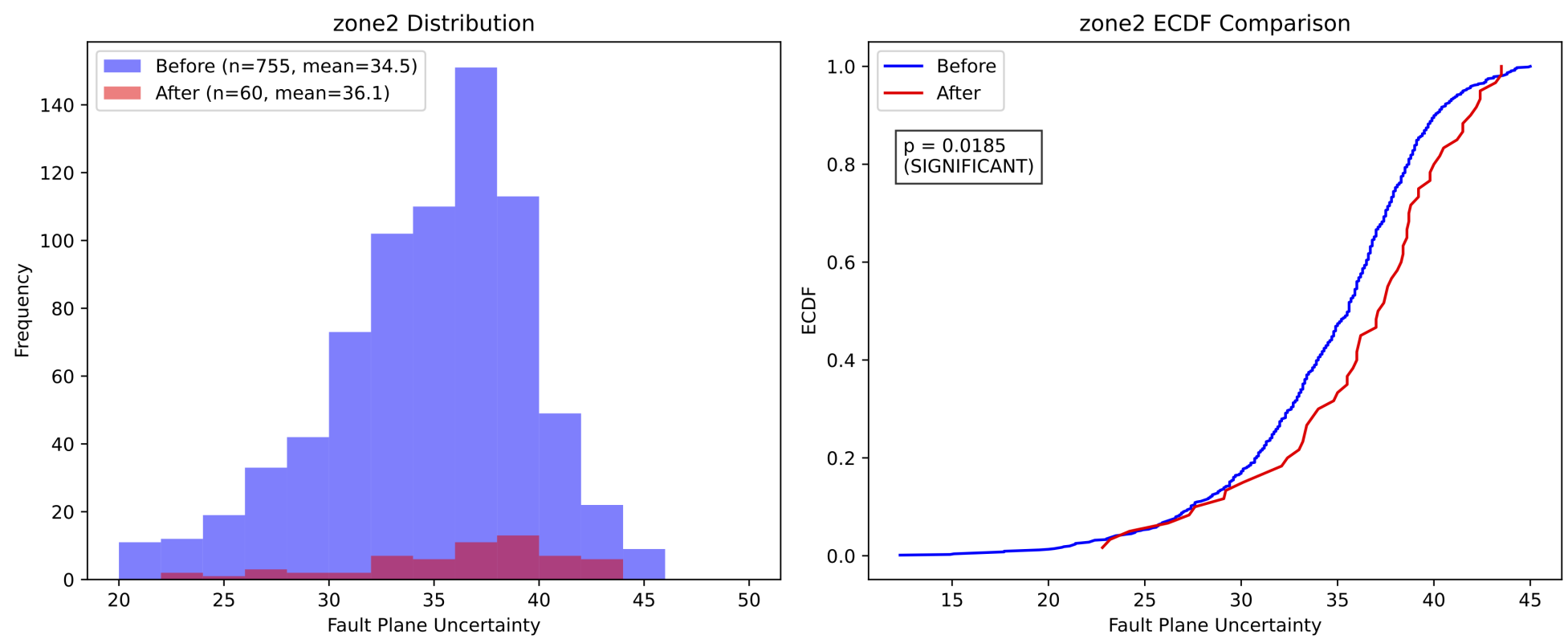

Fig. S7 Focal mechanism fault plane uncertainty for events in Zone 2. Formats and legends are the same as in Fig. S6. The average fault plane uncertainty is 34.5° and 36.1° for events occurring before and after the M6 mainshock, respectively. Here $p < 0.05$ indicates statistically significant difference between the fault plane uncertainty distributions before and after the mainshock.

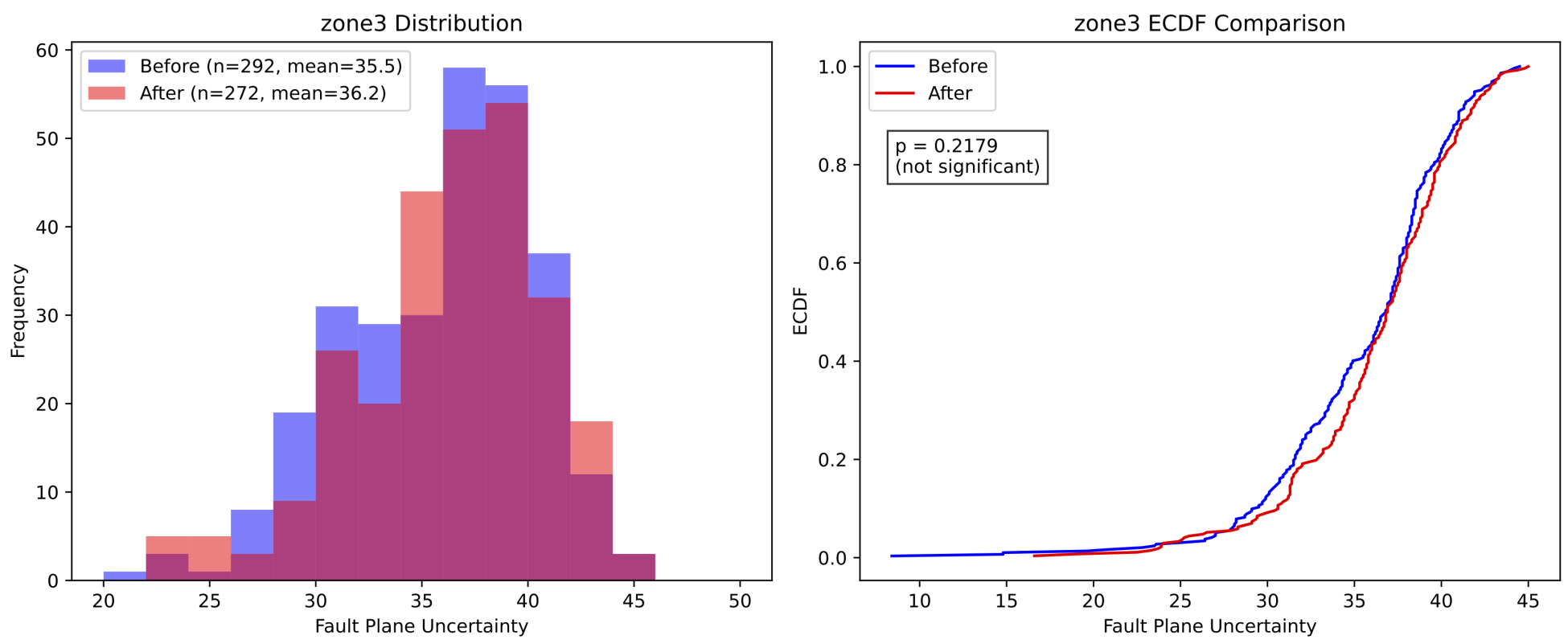

Fig. S8 Focal mechanism fault plane uncertainty in Zone 3. Formats and legends are the same as in Fig. S6. The average fault plane uncertainty is 35.5° and 36.2° for events occurring before and after the M6 mainshock, respectively. Here $p > 0.05$ indicates no statistically significant difference between the fault plane uncertainty distributions before and after the mainshock.

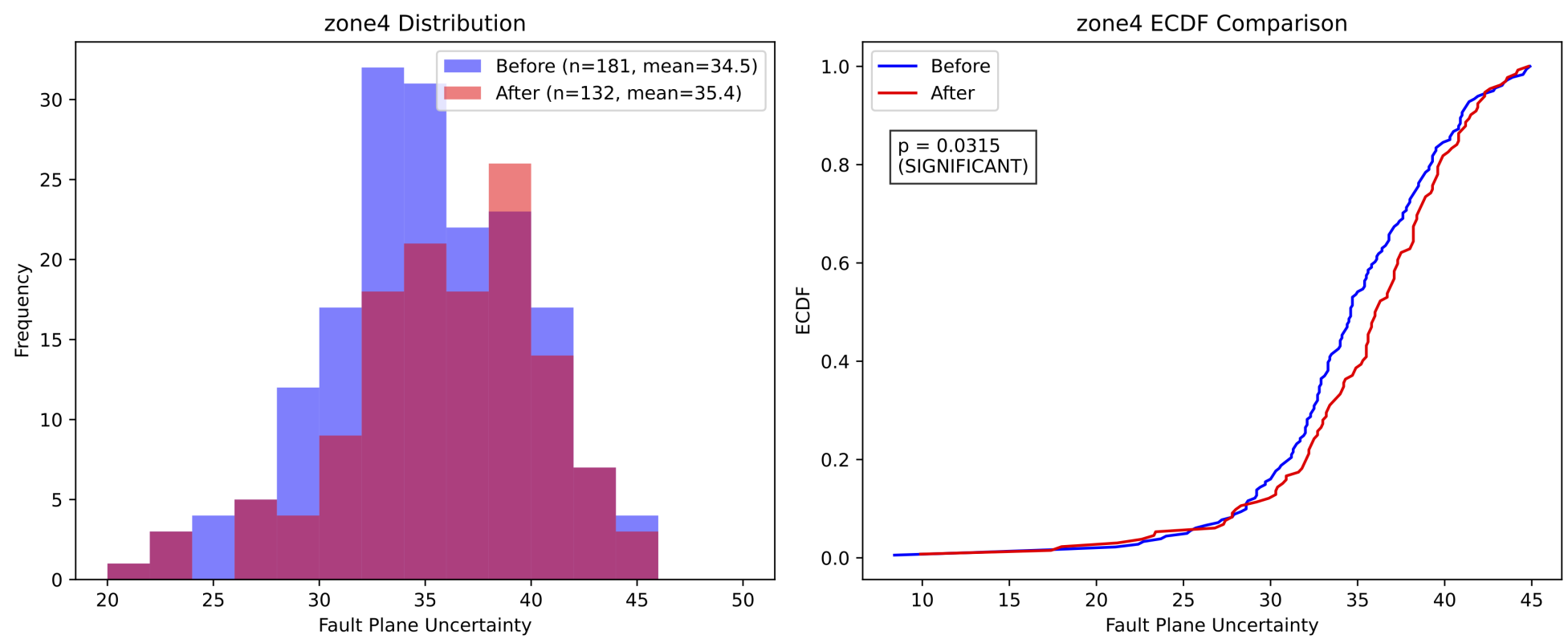

Fig. S9 Focal mechanism fault plane uncertainty in Zone 4. Formats and legends are the same as in Fig. S6. The average fault plane uncertainty is 34.5° and 35.4° for events occurring before and after the M6 mainshock, respectively. Here $p < 0.05$ indicates a statistically significant difference between the fault plane uncertainty distributions before and after the mainshock.

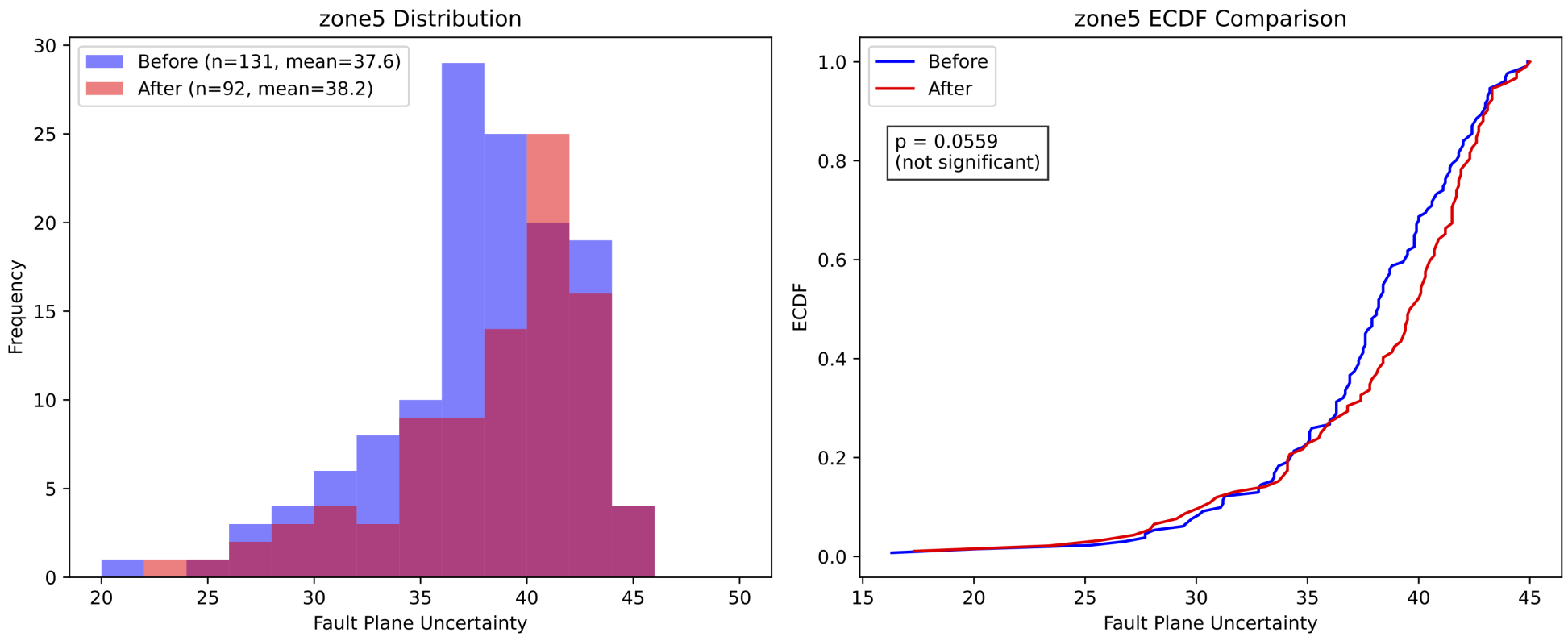

Fig. S10 Focal mechanism fault plane uncertainty in Zone 5. Formats and legends are the same as in Fig. S6. The average fault plane uncertainty is 37.6° and 38.2° for events occurring before and after the M6 mainshock, respectively. Here $p > 0.05$ indicates no statistically significant difference between the fault plane uncertainty distributions before and after the mainshock.

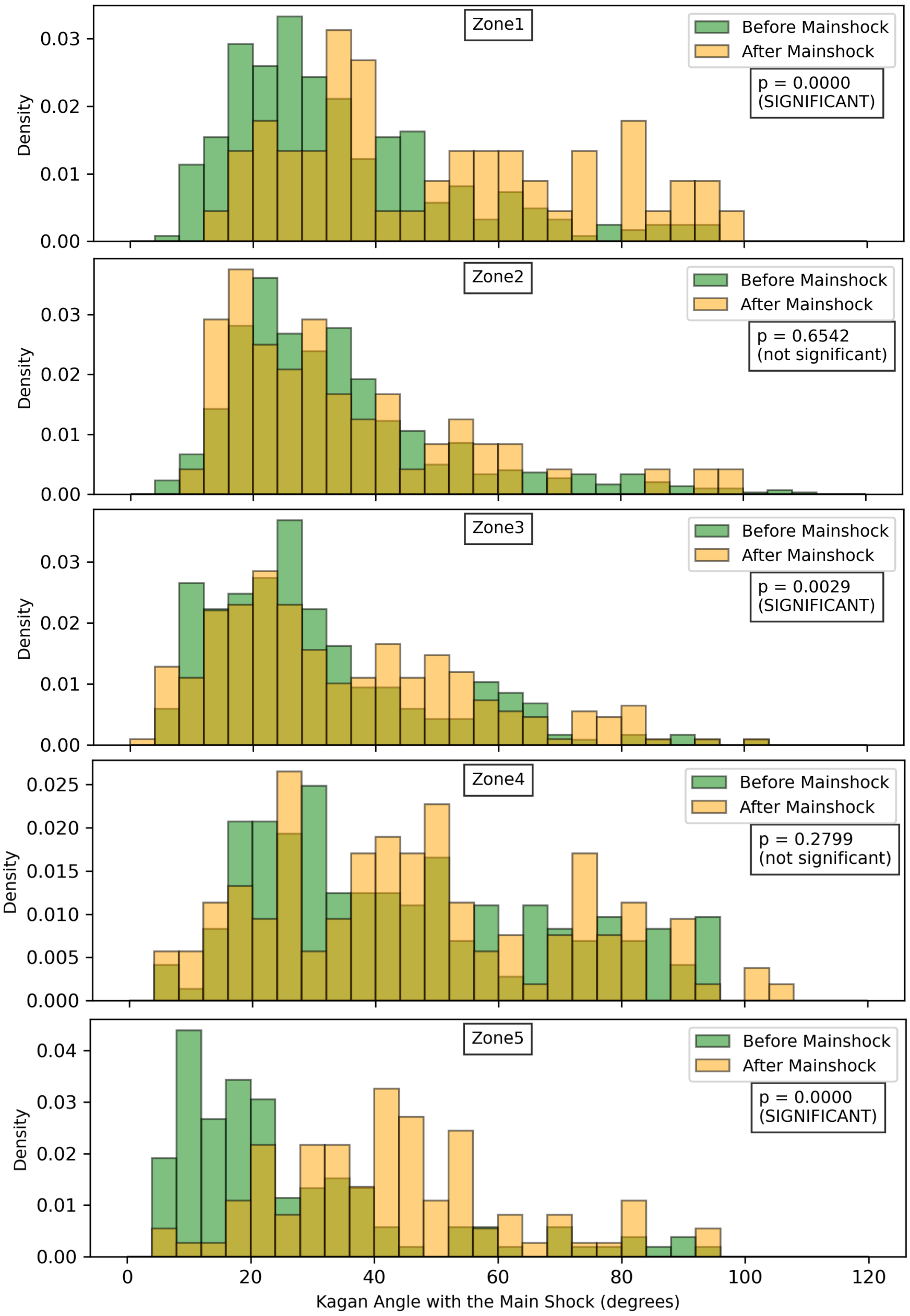

Fig. S11 Histogram (area-normalized to probability density) of the Kagan angle between each micro-earthquake focal mechanism and the M6 mainshock, shown separately for for all five zones. The pre- and post-mainshock distributions are compared with a KS test (see Methods), and the resulting p values are annotated on each panel. In Zones 1, 3 and 5, $p < 0.05$, indicating that the the post-mainshock mechanisms deviate significantly more from the mainshock than pre-mainshock events do. In Zones 2 and 4, the difference is not significant ($p > 0.05$).

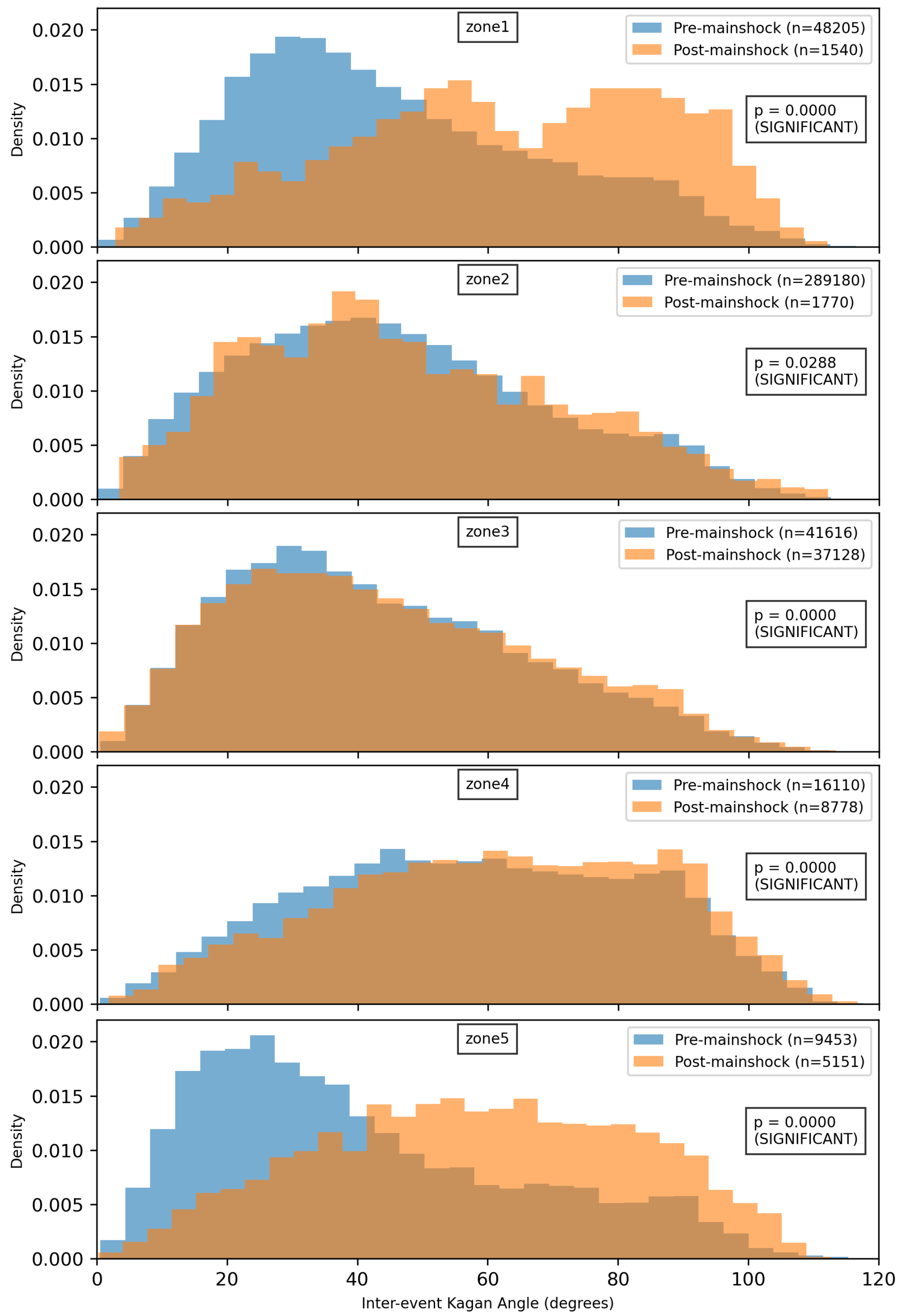

Fig. S12 Histograms (area-normalized to probability density) of the inter-event Kagan angle, shown separately for all five zones. The pre- and post-mainshock distributions are compared with a KS test (see Methods). The event pairs in each time period and the resulting p values are annotated on each panel. All p values are below 0.05 threshold, indicating that mechanism diversity increases significantly after the mainshock in every zone.

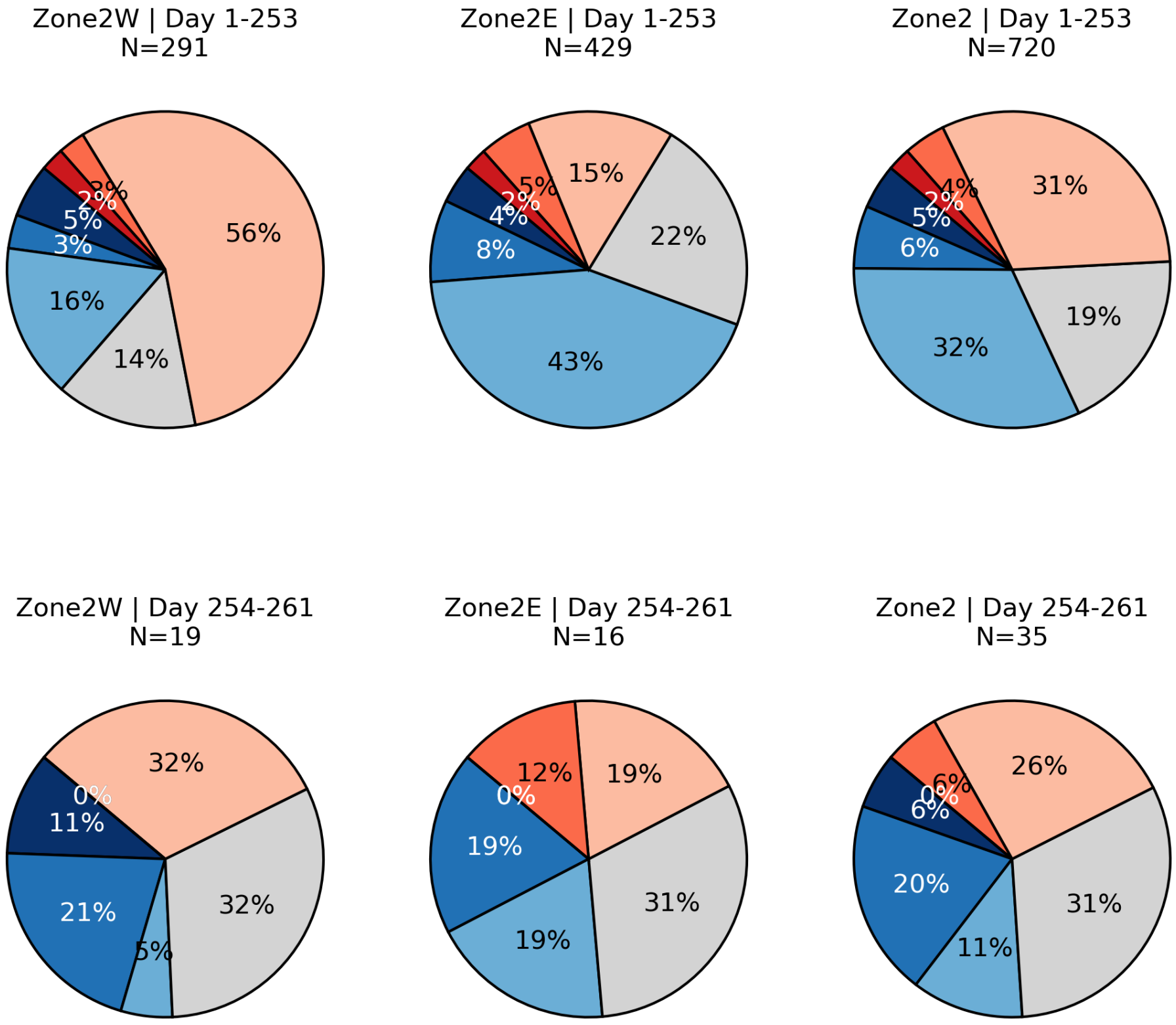

Fig. S13 Faulting type distribution in Zone 2 before and during the foreshock period in 2008. The left, middle and right panels show the west patch, the east patch, and the entire zone (see Fig. 4), respectively. Days 1–253 represent the period before the foreshock sequence; days 254–261 represent the foreshock period. The number of focal mechanism solutions is labeled in each panel. Colors represent faulting type, consistent with Fig. 1. The percentage of each faulting type is labeled on each wedge of the pie chart.

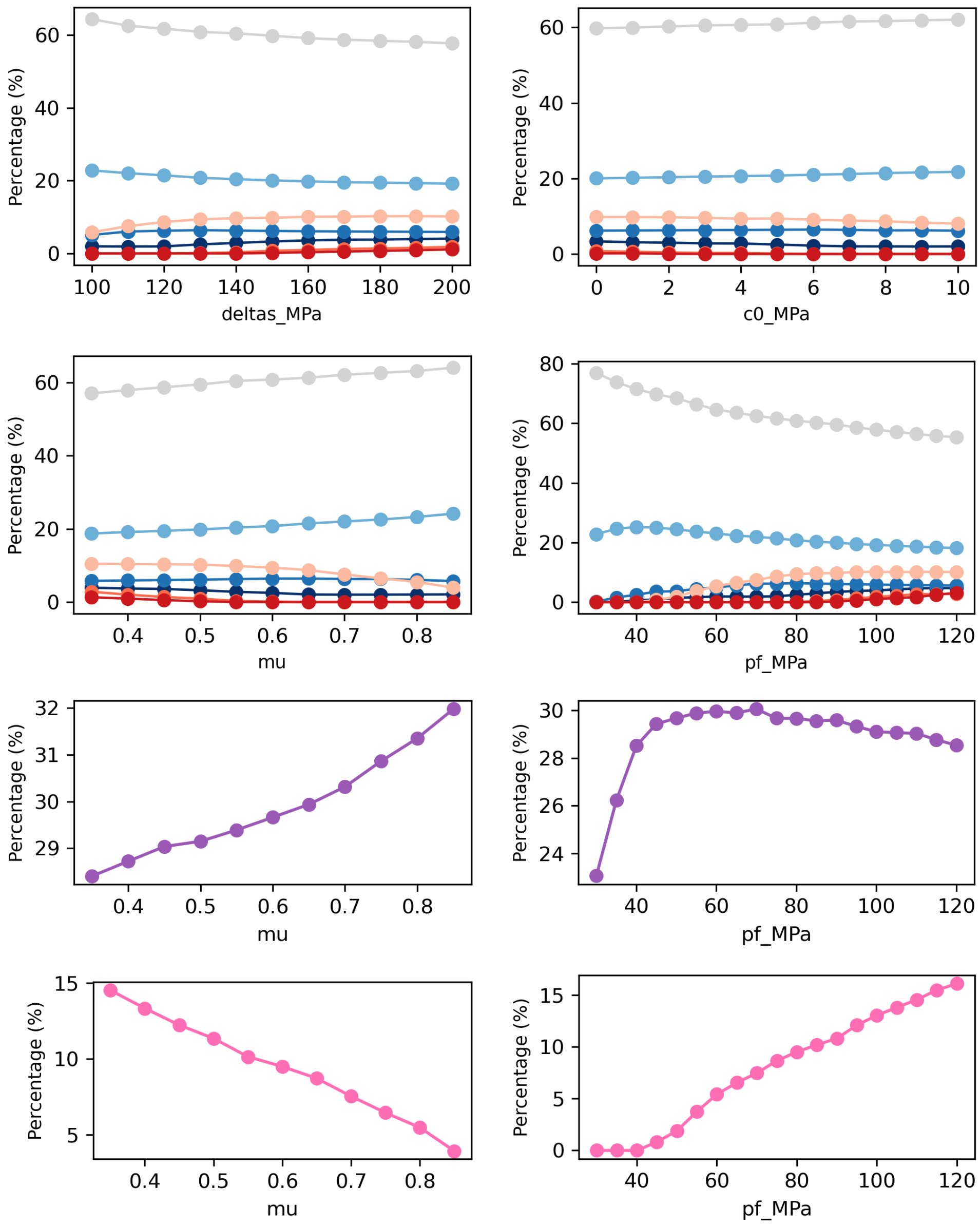

Fig. S14 Coulomb failure simulation results. The top four panels show the percentage of each faulting type as a function of four parameters varied individually: differential stress (deltas), cohesion (c0), frictional coefficient (mu), and pore fluid pressure (pf). In each panel, only the labeled parameter varies while the others are held at their reference values (see Methods). The bottom four panels show how the summed N, N-SS and SS-N percentage (Purple lines) and summed R, R-SS and SS-R percentage (Pink lines) change with respect to frictional coefficient and pore fluid pressure. Note that the proportion of SS events decreases monotonically, and the sum of R, R-SS and SS-R events increases monotonically, with rising pore fluid pressure and/or decreasing frictional coefficient.

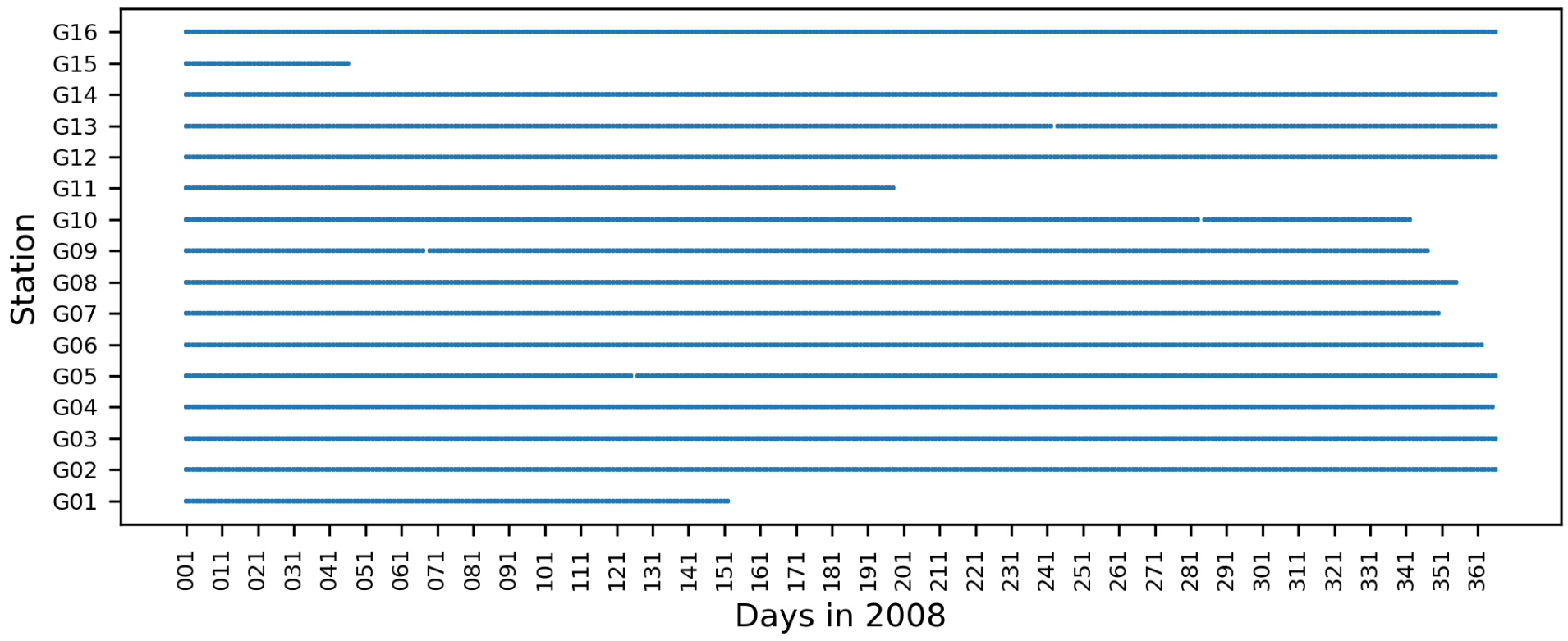

Fig. S15 Station availability in 2008 around the Gofar G3 branch. Blue lines show when each station has all three channels of data available. Stations G1, G11, and G15 failed at different times during the deployment; all other stations operated for most of the year.

	PhaseProb ≥										
PolarityProb ≥	0.00	0.10	0.20	0.30	0.40	0.50	0.60	0.70	0.80	0.90	0.95
0.00	67.6% (598)	67.6% (598)	71.4% (548)	74.9% (513)	77.8% (468)	80.8% (427)	81.8% (396)	83.9% (353)	87.8% (294)	90.1% (182)	98.5% (66)
0.10	67.6% (598)	67.6% (598)	71.4% (548)	74.9% (513)	77.8% (468)	80.8% (427)	81.8% (396)	83.9% (353)	87.8% (294)	90.1% (182)	98.5% (66)
0.20	67.6% (598)	67.6% (598)	71.4% (548)	74.9% (513)	77.8% (468)	80.8% (427)	81.8% (396)	83.9% (353)	87.8% (294)	90.1% (182)	98.5% (66)
0.30	67.6% (598)	67.6% (598)	71.4% (548)	74.9% (513)	77.8% (468)	80.8% (427)	81.8% (396)	83.9% (353)	87.8% (294)	90.1% (182)	98.5% (66)
0.40	67.6% (598)	67.6% (598)	71.4% (548)	74.9% (513)	77.8% (468)	80.8% (427)	81.8% (396)	83.9% (353)	87.8% (294)	90.1% (182)	98.5% (66)
0.50	67.6% (598)	67.6% (598)	71.4% (548)	74.9% (513)	77.8% (468)	80.8% (427)	81.8% (396)	83.9% (353)	87.8% (294)	90.1% (182)	98.5% (66)
0.60	69.1% (576)	69.1% (576)	72.6% (530)	76.2% (496)	79.2% (452)	82.1% (414)	83.4% (385)	85.0% (346)	88.6% (289)	91.5% (177)	98.5% (66)
0.70	70.2% (551)	70.2% (551)	73.7% (509)	77.5% (475)	80.5% (436)	83.3% (400)	84.7% (373)	86.3% (336)	90.4% (280)	92.5% (174)	98.5% (66)
0.80	71.6% (524)	71.6% (524)	74.7% (486)	78.6% (453)	81.2% (421)	84.0% (388)	85.6% (362)	87.5% (328)	91.6% (273)	94.1% (169)	98.5% (66)
0.90	75.8% (475)	75.8% (475)	78.6% (444)	82.7% (415)	85.0% (387)	87.2% (359)	88.5% (338)	89.9% (308)	93.1% (261)	94.6% (166)	98.5% (66)
0.95	80.0% (430)	80.0% (430)	82.9% (403)	85.7% (384)	88.0% (358)	89.9% (335)	90.9% (317)	91.8% (291)	94.0% (250)	95.7% (163)	100.0% (65)
0.99	82.4% (374)	82.4% (374)	84.7% (354)	87.6% (338)	89.8% (315)	90.9% (297)	91.9% (284)	93.1% (260)	94.8% (230)	97.4% (151)	100.0% (64)

Fig. S16 Summary of visual inspection of polarity picks. Each cell shows the percentage of automatic polarity picks (from RPNet) that are consistent with manual picks, as a function of the phase-picking probability threshold (horizontal axis) and the polarity-picking probability threshold (vertical axis). The number in the bracket represents the number of polarities retained with the combined thresholds. Darker colors indicate higher consistency. For traces whose polarity cannot be decided manually, the automatic pick is counted as inconsistent. The box highlights the preferred threshold combination, phase picking probability > 0.3 and polarity picking probability > 0.95, yielding a consistency of 85.7%.

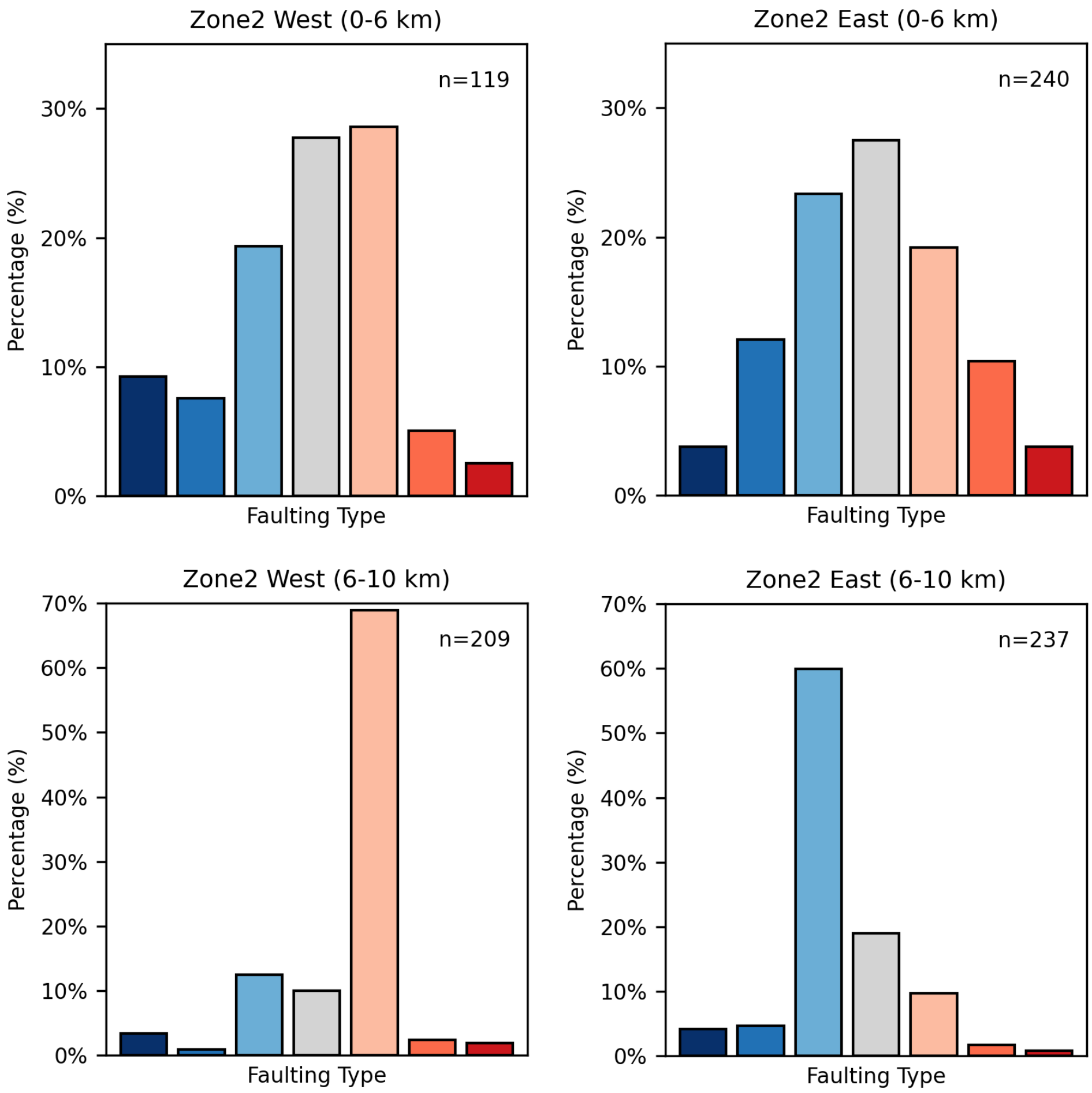

Fig. S17 Histograms of faulting types in Zone 2, separated by subzone (west and east patches) and depth cluster (shallow and deep). The colors are consistent with Fig. 4 in the main text.

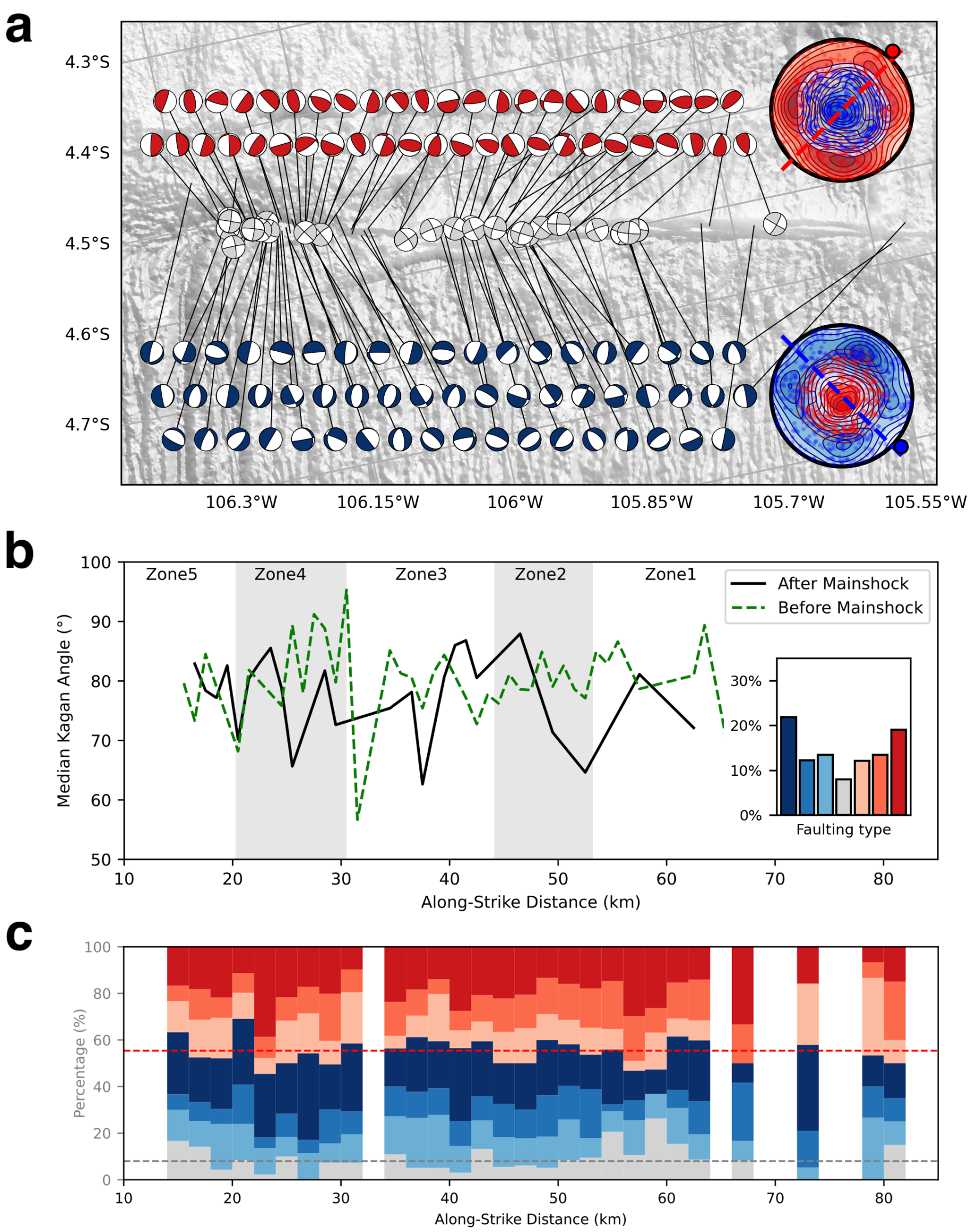

Fig. S18 Random polarity test results. **a**, Synthetic focal mechanisms for all $M \geq 2.2$ events with faulting type as N, R, and SS, shown by blue, red, and grey focal mechanisms, respectively. **Top inset**, P- and T-axis distributions for R events, shown by red and blue dots, respectively. The dashed line is the same as in Fig. 2. **Bottom inset**, P- and T-axis distributions for N events, shown in the same format. **b**, Median Kagan angle in each 1-km along strike bin, shown separately for pre- and post-mainshock periods. **Inset**, Histogram of faulting types. c, Along-strike variation of faulting types. Unlike the real catalog (Fig. 3b), the random test shows no systematic along-strike segmentation of faulting types.

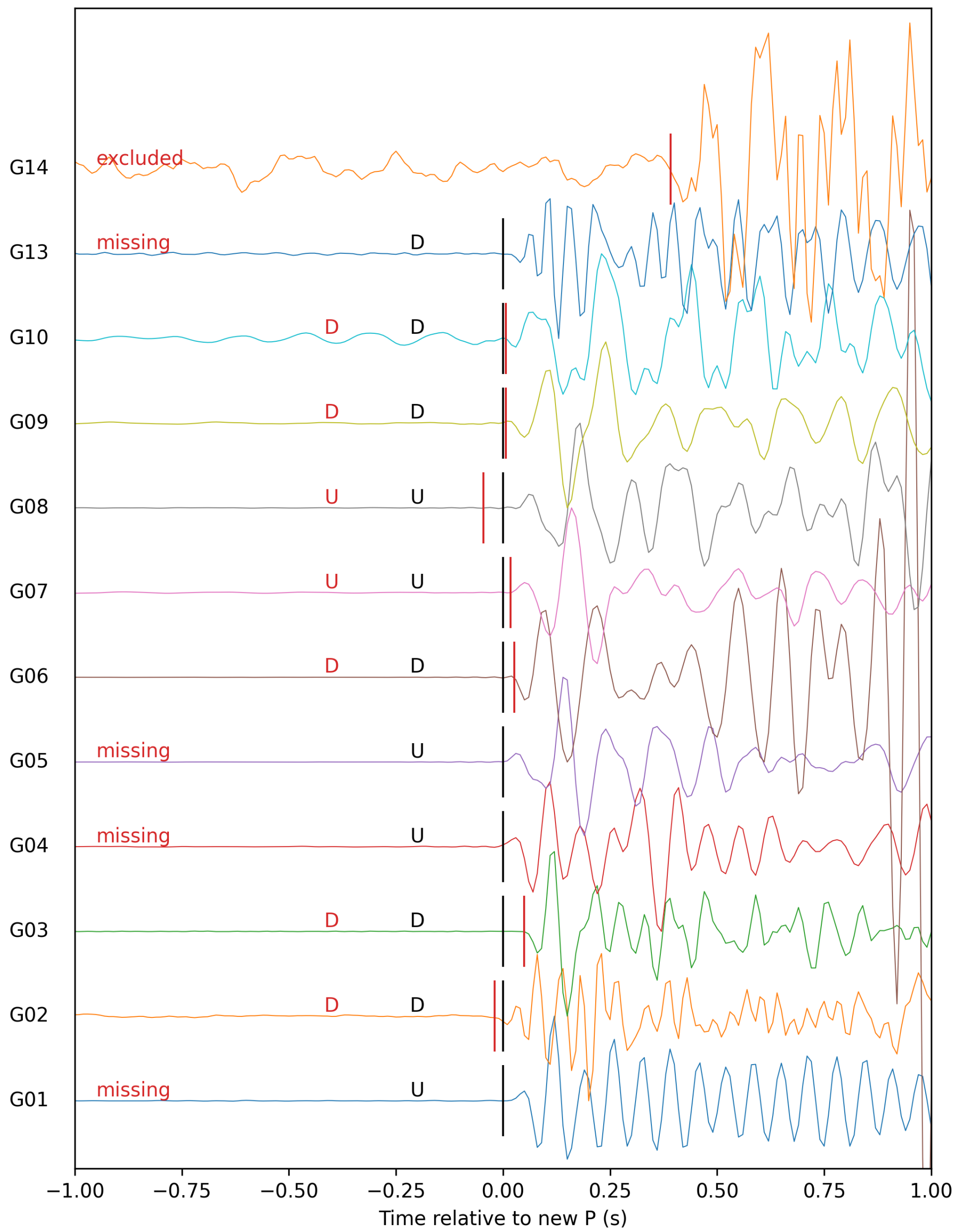

Fig. S19 Comparison of P-wave onset picks between Gong and Fan (2022) [2] and this study. Event 800053 is used as an example for demonstration (first example event in Fig. S1). High-pass filtered (5 Hz) waveforms from the vertical component are shown for each station. Red and black lines mark the P onset times in Gong and Fan (2022) and this study, respectively. Polarities in red and black are obtained by RPNet [1] with trace input assuming onset time of Gong and Fan (2022) and this study, respectively. P onsets are not available at four station (marked as "missing") in Gong and Fan (2022). The polarity at G14 is excluded due to its probability is <0.95. By using picks from Gong and Fan (2022), only seven polarities will be retained and the focal mechanism will not be resolved.

Table S1 Size of published focal mechanism/moment tensor datasets for earthquakes on oceanic transform faults.

Name	Number	Data	References
15°20'	2	Teleseismic	Engeln et al., 1986 [9]
Chain	4	Teleseismic	Engeln et al., 1986 [9]
Doldrums	3	Teleseismic	Engeln et al., 1986 [9]
Gibbs	6	Teleseismic	Engeln et al., 1986 [9]
Hays	4	Teleseismic	Engeln et al., 1986 [9]
Kane	1	Teleseismic	Engeln et al., 1986 [9]
Romanche	7	Teleseismic	Engeln et al., 1986 [9]
St. Paul	5	Teleseismic	Engeln et al., 1986 [9]
Vema	4	Teleseismic	Engeln et al., 1986 [9]
15°20'	3	Teleseismic	Bergman & Solomon, 1988 [10]
Gibbs	2	Teleseismic	Bergman & Solomon, 1988 [10]
Hays	1	Teleseismic	Bergman & Solomon, 1988 [10]
Kane	2	Teleseismic	Bergman & Solomon, 1988 [10]
Oceanographer	1	Teleseismic	Bergman & Solomon, 1988 [10]
Vema	3	Teleseismic	Bergman & Solomon, 1988 [10]
Kane	6	OBS	Wilcock et al., 1990 [11]
Blanco	2	Teleseismic	Wolfe et al., 1993 [12]
Discovery II	1	Teleseismic	Wolfe et al., 1993 [12]
Fracture Zone VI	1	Teleseismic	Wolfe et al., 1993 [12]
Gofar	1	Teleseismic	Wolfe et al., 1993 [12]
Heezen	2	Teleseismic	Wolfe et al., 1993 [12]
Marathon	1	Teleseismic	Wolfe et al., 1993 [12]
Menard	1	Teleseismic	Wolfe et al., 1993 [12]
Owen	3	Teleseismic	Wolfe et al., 1993 [12]
Rivera	1	Teleseismic	Wolfe et al., 1993 [12]
St. Paul	7	Teleseismic	Wolfe et al., 1993 [12]
Tharp	2	Teleseismic	Wolfe et al., 1993 [12]
Udintsev	1	Teleseismic	Wolfe et al., 1993 [12]
Unnamed Transforms	3	Teleseismic	Wolfe et al., 1993 [12]
Blanco	43	Teleseismic	Dziak et al., 2000 [13]
Chain	6	Teleseismic	Abercrombie & Ekström, 2001 [14]
Romanche	8	Teleseismic	Abercrombie & Ekström, 2001 [14]
Blanco	157	Teleseismic	Braunmiller & Nábělek, 2008 [15]
Jan Mayen	11	Teleseismic	Rodríguez-Pérez & Ottemöller, 2014 [16]
Charlie-Gibbs	2	Teleseismic	Aderhold & Abercrombie, 2016 [17]
Blanco	185	OBS	Kuna, 2020 [18]
Romanche	6	OBS & Teleseismic	Yu et al., 2021 [19]
Blanco	84	OBS	Ren et al., 2023 [20]
Chain	88	OBS	Schlaphorst et al., 2023 [21]
Vernadsky	7	Teleseismic	de Melo et al., 2024 [22]
Oceanographer	13	OBS	Grevemeyer et al., 2025 [23]
St. Paul A	22	Teleseismic	de Melo et al., 2025 [24]
St. Paul B	5	Teleseismic	de Melo et al., 2025 [24]
St. Paul C	8	Teleseismic	de Melo et al., 2025 [24]
164 transform faults globally	2–140/transform fault	Teleseismic	Janin et al., 2026 [25]
Gofar G3	2,323	OBS	This study

Table S2 SKHASH parameter settings used for focal mechanism inversion.

Parameter	Description	Value
`$npolmin`	minimum number of polarity data	8
`$max_agap`	maximum azimuthal gap (°)	180
`$max_pgap`	maximum "plungal" gap (°)	90
`$dang`	minimum grid spacing (°)	5
`$nmc`	number of trials	30
`$maxout`	max number of acceptable focal mechanism outputs	10000
`$badfrac`	fraction of polarities assumed bad	0.1
`$badmin`	minimum number of bad polarities	1
`$delmax`	maximum allowed source–receiver distance (km)	120
`$cangle`	angle for computing mechanisms probability (°)	45
`$prob_max`	probability threshold for multiples	0.25
`$iterative_avg`	iterative averaging	True

References

[1] Han, J., Kim, S. & Sheen, D.-H. RPNet: Robust P -Wave First-Motion Polarity Determination Using Deep Learning. *Seismological Research Letters* (2025).

[2] Gong, J. & Fan, W. Seismicity, fault architecture, and slip mode of the westernmost gofar transform fault. *Journal of Geophysical Research: Solid Earth* **127**, e2022JB024918 (2022).

[3] Mousavi, S. M., Ellsworth, W. L., Zhu, W., Chuang, L. Y. & Beroza, G. C. Earthquake transformer—an attentive deep-learning model for simultaneous earthquake detection and phase picking. *Nature Communications* 3952 (2020).

[4] Bornstein, T. *et al.* PickBlue: Seismic Phase Picking for Ocean Bottom Seismometers With Deep Learning. *Earth and Space Science* **11**, e2023EA003332 (2024).

[5] Roland, E., Lizarralde, D., McGuire, J. J. & Collins, J. A. Seismic velocity constraints on the material properties that control earthquake behavior at the quebrada-discovery-gofar transform faults, east pacific rise. *Journal of Geophysical Research: Solid Earth* **117** (2012).

[6] Álvarez Gómez, J. A. FMC—Earthquake focal mechanisms data management, cluster and classification. *SoftwareX* **9**, 299–307 (2019).

[7] Kagan, Y. Y. Simplified algorithms for calculating double-couple rotation. *Geophysical Journal International* **171**, 411–418 (2007).

[8] Fan, W. *et al.* Dynamics of rupture barriers on oceanic transform faults: Insights from the westernmost gofar transform fault. *Journal of Geophysical Research: Solid Earth* **131**, e2025JB033439 (2026).

[9] Engeln, J. F., Wiens, D. A. & Stein, S. Mechanisms and depths of Atlantic transform earthquakes. *Journal of Geophysical Research: Solid Earth* **91**, 548–577 (1986).

[10] Bergman, E. A. & Solomon, S. C. Transform fault earthquakes in the North Atlantic: Source mechanisms and depth of faulting. *Journal of Geophysical Research: Solid Earth* **93**, 9027–9057 (1988).

[11] Wilcock, W. S., Purdy, G. & Solomon, S. C. Microearthquake evidence for extension across the kane transform fault. *Journal of Geophysical Research: Solid Earth* **95**, 15439–15462 (1990).

[12] Wolfe, C. J., Bergman, E. A. & Solomon, S. C. Oceanic transform earthquakes with unusual mechanisms or locations: Relation to fault geometry and state of stress in the adjacent lithosphere. *Journal of Geophysical Research: Solid Earth*

98, 16187–16211 (1993).

[13] Dziak, R. P. *et al.* Recent tectonics of the Blanco Ridge, eastern blanco transform fault zone. *Marine Geophysical Researches* **21**, 423–450 (2000).

[14] Abercrombie, R. E. & Ekström, G. Earthquake slip on oceanic transform faults. *Nature* **410**, 74–77 (2001).

[15] Braunmiller, J. & Nábělek, J. Segmentation of the Blanco Transform Fault Zone from earthquake analysis: Complex tectonics of an oceanic transform fault. *Journal of Geophysical Research: Solid Earth* **113** (2008).

[16] Rodríguez-Pérez, Q. & Ottemöller, L. Source study of the Jan Mayen transform fault strike-slip earthquakes. *Tectonophysics* **628**, 71–84 (2014).

[17] Aderhold, K. & Abercrombie, R. E. The 2015 Mw 7.1 earthquake on the Charlie-Gibbs transform fault: Repeating earthquakes and multimodal slip on a slow oceanic transform. *Geophysical Research Letters* **43**, 6119–6128 (2016).

[18] Kuna, V. M. *Investigation of Slip and Tectonics of the Blanco Transform Fault using High-Resolution Ocean Bottom Seismic Data.* Ph.D. thesis, Oregon State University (2020).

[19] Yu, Z. *et al.* Semibrittle seismic deformation in high-temperature mantle mylonite shear zone along the Romanche transform fault. *Science Advances* **7**, eabf3388 (2021).

[20] Ren, Y., Lange, D. & Grevemeyer, I. Seismotectonics of the blanco transform fault system, northeast pacific: Evidence for an immature plate boundary. *Journal of Geophysical Research: Solid Earth* **128**, e2022JB026045 (2023).

[21] Schlaphorst, D. *et al.* Local seismicity around the Chain Transform Fault at the Mid-Atlantic Ridge from OBS observations. *Geophysical Journal International* **234**, 1111–1124 (2023).

[22] De Melo, G. W. S., Mitchell, N. C. & Sokolov, S. Y. The 2020 Mw 6.6 Vernadsky transform earthquake sequence: rupture and Coulomb stress changes surrounding an oceanic core complex. *Marine Geophysical Research* **45**, 28 (2024).

[23] Grevemeyer, I. *et al.* Strike-Slip Versus Extensional Tectonics at the Oceanographer Transform Fault, Mid-Atlantic Ridge at 35°N. *Journal of Geophysical Research: Solid Earth* **130**, e2024JB030880 (2025).

[24] de Melo, G. W. S., Grevemeyer, I., Liu, S., Maia, M. & Rüpke, L. Seismicity and thermal structure of the St. Paul Transform System, equatorial Atlantic: insights from focal depth analysis. *Solid Earth* **16**, 1059–1072 (2025).

[25] Janin, A., Behn, M. D. & Tian, X. Geometry, structure and tectonic regime of oceanic transform faults revealed by teleseismic earthquake focal mechanisms. *Geophysical Journal International* **245**, ggag078 (2026).
