# Supplementary material for: Focal mechanism variations reveal mechanical controls on segmentation at oceanic transform faults
Source: Nat Commun. 2026 Aug 15;17:9836. doi: 10.1038/s41467-026-76407-5 (PMC13578451; doi:10.1038/s41467-026-76407-5)
Supplement: Supplementary file 2 [file 41467_2026_76407_MOESM3_ESM.pdf]

nature portfolio

Peer Review File

Focal mechanism variations reveal mechanical controls on segmentation at oceanic transform faults

Corresponding Author: Dr Fengzhou Tan

This file contains all reviewer reports in order by version, followed by all author rebuttals in order by version.

Attachments originally included by the reviewers as part of their assessment can be found at the end of this file.

Parts of this Peer Review File have been redacted as indicated to remove third-party material.

Version 0:

Reviewer comments:

Reviewer #1

(Remarks to the Author)
Review for manuscript 'Variations in mechanical properties control segmentation of oceanic transform faults' by F. Tan, W. Fan, P. M. Shearer, M. D. Behn, and J. J. McGuire for publication in Nature Communications.

The manuscript analyzes 1-year of OBS data from the western Gofar oceanic transform fault in the EPR region. The dataset is unique and captured a (forecasted) M6 earthquake that ruptured one segment (zone 3 in the manuscript) of the fault. The authors use an existing earthquake catalog to obtain first motion polarities creating a focal mechanism database of over 3000 fault plane solutions, which is unique for an OTF. The authors find a wide variety of faulting styles rather than the expected strike-slip deformation. Differences are related to fault segmentation and the mechanisms in some areas change following the main shock indicating activation of secondary faulting features. The prevalence of non-strike slip mechanisms imply that the OTF plate boundary is complex and perhaps is non-conservative (extension and compression) in nature.

The manuscript is very easy to follow and very well written. The results are new for OTF environments and represent significant new knowledge about OTF faulting behavior and space-time variability. The analysis is described well and the authors make it sound straight forward. The methods used are sound and sufficient detail is provided.

This is a significant contribution with noteworthy results. I have only a few comments and questions.

With best regards,
Jochen Braunmiller

——

General questions:

- For the focal mechanism classification (Kaverina diagram and classification scheme by Alvarez Gomez): Given all possible double couple solutions, would each group contain 1/7 of all events? I am not sure but Figure 1 bottom inset suggests different types may occur with different likelihood for a random draw. This does not affect the focal mechanism variability that you observe but could affect interpretation of histograms and percentages (slighly). I am just wondering whether the classification makes it 'more difficult' for an event to be declared SS than let's say N or SS-R. For an evenly-spaced distribution of double couple focal mechanisms, would 1/7 be classified as SS?

- Are there any noticeable (or significant) changes in mechanisms with event depth? The manuscript includes several cross-sections (Figs. 1b, 4b,e) but the text does not cover this aspect. Why?

- What, in detail, is the angular difference attributed to Hardebeck and Hauksson [2001]? Their paper does not (sufficiently) define it. The Kagan angle is also a measure of (deviatoric moment tensor) difference and is used throughout the manuscript. What does the 'inter-event angular difference' add (or why is it preferred for some instances in the manuscript)?

Main Text:

Line L94 and L122. The spacing is not clear. In line L94 it says '2x2 km along strike' but line L91 mentions a ~ 5 km wide strike-normal spread. Were there several bins at a given along-strike distance to cover the width? Or did you restrict the analysis to events within +-1 km of the central fault trace? — Similarly for L122. What width have the 1-km spatial bins? — This becomes clearer when looking at the figures in the main text and supplement and perhaps that is already sufficient.

L275-287. This sounds interesting but is not well-developed. What does the co-existence of valleys and compressional bends imply for the suggested 'magma overshoot'? The valley is a reflection of lack of magma overshoot with the bends being some (other) structural feature unrelated to magma supply? It seems there's magma 'overshoot' and 'undershoot' at the same time but different locations? — That part is not clear.

Figure 3. Perhaps change to 'Average Kagan angle relative to the 2008 M6 mainshock in each …' for people who only look at figures. — 'The white curve and dots represent the average stress drop,' change 'black' to 'white'. — Comment: The largest Kagan angles seem to be in the strongly couples M6 zones Z3 and Z1; interesting.

Methods section:

L312. Why only events with a reported magnitude? I assume because others in the catalog are smaller and less likely to have sufficient signal. You may want to say something.

L322. You may want to add that the data were recorded with 100 sps. — Using 4 samples seems arbitrary and potentially unstable [I guess your results show otherwise], what was the reason for using such a short window and did you conduct tests to establish that 4 samples is a good number; also to start at the third sample after the pick. Perhaps add a sentence explaining the choice would be helpful. Perhaps point to the supplement as Fig. S1 shows how short the pulses are.

Supplement:

Figure S1. Did you use the same 1D velocity model for calculating the piercing points as you used for the P onset picking? This is not clear, but you do not mention any other velocity model in the manuscript. — These are relatively strong, and deep aftershocks?

Figure S2. The 'maximum horizontal stress direction' is very difficult to see in the top panel. As this is shown in the middle panel, perhaps eliminate from top panel? — Perhaps you could somewhere say explicitly what a stress ratio of 0.65 means in terms of the relative eiganvalue sizes?

Figure S3. correct to 'same to those in Fig. S2' and again 'S2' in next sentence. (same correction in caption of Fig. S4)

Figure S6. 'The p>0.05 …' (same correction in caption of Fig. S7)

Figure S10. 'mainshock' correct spelling.

Figure S13. Last sentence in caption is garbled, do you mean '… is labelled …'?

Reviewer #2

(Remarks to the Author)

Reviewer #3

(Remarks to the Author)
The manuscript presents results from one of the relatively few studies reporting focal mechanisms at an oceanic transform fault (OTF) using a dense network of ocean-bottom seismometers (OBSs). I appreciate the considerable effort made by the authors to investigate faulting styles and stress variations across multiple predefined zones along the western segment (G3) of the Gofar transform fault. This topic is undoubtedly of interest to the broader geoscience community. However, after a careful reading of the manuscript, I have several substantial concerns that need to be addressed before the study can be considered for publication. The detailed comments are provided below.

Comment 1: My primary concern is on the data and methods used in this study.
(1) I was surprised to find that the OBS dataset is described in only 1-2 sentences. The authors state that data from 16 OBS stations were used. However, it is known that not all stations in this experiment recorded high-quality or complete data throughout the deployment. Some stations reportedly failed shortly after deployment, and missing components are relatively common. Given these known complexities, the manuscript needs a much more detailed description of the OBS dataset, including station availability through time and data quality control. This information is critical because the study emphasizes spatiotemporal variations in faulting and stress. Changes in network geometry, station coverage, or component availability could bias the results if not carefully accounted for. The authors should explicitly demonstrate that the reported patterns are not artifacts of evolving network geometry or data completeness.
(2) The focal mechanism catalog is constructed based on the microearthquake catalog of Gong and Fan (2022), which

contains more than 30,000 events. In contrast, the present study analyzes only approximately 6,000 events. The manuscript does not explain why this relatively small subset was selected or what criteria led to the exclusion of the remaining events. In addition, the magnitude range of the analyzed events, from –0.55 to 4.3, indicates that the largest earthquakes, including the M6 events known to occur on Gofar and several events larger than M4.5, were excluded. This is surprising, as larger earthquakes typically have clearer onsets and more reliable polarity information and can serve as valuable benchmark cases for validating focal mechanism (FM) solutions. The authors should clearly justify the exclusion of large events and discuss whether their omission could affect the robustness or representativeness of the results.
(3) The authors re-picked the P arrivals using PickBlue, which seems to have better performance on OBS data. While this is reasonable, the manuscript does not explain why re-picking was necessary given that Gong and Fan (2022) already picked arrivals using EQTransformer. A comparison between EQTransformer and PickBlue picks would be very helpful to justify this step and to demonstrate how the new picks improve the catalog. Related questions that should be clarified include the following. Does the re-picking imply that the previous catalog has significant timing uncertainties? Were these earthquake re-located using the new picks? Were magnitudes re-estimated accordingly? Furthermore, I find it puzzling that a PickBlue probability threshold of 0.3 was adopted to define "valid" P picks, although Bornstein et al. (2024) recommended substantially higher thresholds to effectively remove outliers. The authors should explain why 0.3 is considered optimal in this case and provide evidence that this choice does not introduce excessive noise or bias. Although these points may appear technical, they directly affect the foundation of the FM catalog. These issues do not necessarily need to be fully detailed in the main text, but they should be carefully addressed, at least in the Methods or Supplementary Information, to validate the reliability of the dataset.
(4) The resulting FM catalog shows diverse faulting styles along the G3 segment, which is potentially an interesting finding. However, before making strong statements about spatial variations in faulting style, the quality of the FM solutions needs to be more rigorously constrained. The cited work by Parnell-Turner et al. (2017) only accepted FMs with RMS fault plane uncertainty ≤35°, weighted fraction of misfit polarities <20%, station distribution radio >0.4, mechanism probability ≥0.6, and azimuthal gap ≤90°. These criteria are much more stricter than the single criterion (i.e., azimuthal gap <180°) used in this study. Applying stricter, multi-parameter quality criteria would likely reduce the number of accepted mechanisms but would significantly increase confidence in the remaining solutions. In this context, quality should be prioritized over quantity. Finally, I note that a more recent OBS deployment in the G3 segment, network code 8A, is now available. Combining data from both the ZD and 8A networks could substantially strengthen the conclusions regarding mechanical properties along G3 and would likely improve station coverage and azimuthal constraints.

Comment 2: Based on the title, I initially expected the manuscript to include results from multiple oceanic transform faults. However, the study focuses exclusively on the western segment of the Gofar transform fault, G3. I am also not fully convinced by the statement that G3 is "representative" of global OTFs, which is supported only by brief citations to Wolfson-Schwehr et al. (2019) and Shi et al. (2021). These studies do not, in my view, provide strong justification for such a broad claim. Given the limited number of OBS experiments conducted at OTFs, G3 is one of the best-studied examples, which allows for more detailed analysis, but this does not necessarily make it representative. The authors should either provide a much more detailed justification for this claim or avoid it altogether. Because the manuscript is tightly focused on a localized region, I strongly suggest revising the title to replace "oceanic transform faults" with "the western Gofar transform fault," and modifying related statements throughout the manuscript accordingly.

Comment 3: The manuscript often assumes that readers are already familiar with previous works conducted in the area, or the study area itself. For example, the Introduction refers to the western strand of Gofar as "G3" without explaining how the overall fault system is segmented. This makes the manuscript difficult to follow for readers who are not specialists in this region. Including a clear map showing the location of the Gofar transform fault, its segmentation, and the position of G3 would greatly improve accessibility. Additionally, the manuscript states that G3 contains two seismic segments that rupture approximately every five years in M6 earthquakes (lines 48-49), but neither the segmentation nor the locations of historical M6 events are clearly illustrated. Showing these features on a map would provide essential context.
Finally, while the authors note that G3 can be divided into five zones (lines 56-60), the manuscript would benefit from a brief discussion of the outstanding questions motivating this subdivision. This would create a clearer narrative bridge between prior studies and the specific objectives of the present work.

Comment 4: Several relevant studies are unfortunately overlooked. In the Introduction, the authors state that observations of faulting variations and stress regimes at OTFs are limited, without citing prior work. In fact, a number of studies, spanning several decades, have targeted at these topics, at least in part. Recent examples include but not limited to:
Kuna, V. (2020). Investigation of Slip and Tectonics of the Blanco Transform Fault using High-resolution Ocean Bottom Seismic Data. : Oregon State University.
Leptokaropoulos, K., Rychert, C.A., Harmon, N. et al. Broad fault zones enable deep fluid transport and limit earthquake magnitudes. Nat Commun 14, 5748 (2023). https://doi.org/10.1038/s41467-023-41403-6
Ren, Y., Lange, D., & Grevemeyer, I. (2023). Seismotectonics of the Blanco transform fault system, northeast Pacific: Evidence for an immature plate boundary. Journal of Geophysical Research: Solid Earth, 128, e2022JB026045. https://doi.org/10.1029/2022JB026045
Grevemeyer, I., Lange, D., Klaucke, I., Ruepke, L. H., Beniest, A., Gómez de la Peña, L., et al. (2025). Strike-slip versus extensional tectonics at the Oceanographer transform fault, Mid-Atlantic Ridge at 35°N. Journal of Geophysical Research: Solid Earth, 130, e2024JB030880. https://doi.org/10.1029/2024JB030880

These and other studies should be acknowledged to more clearly define the existing knowledge base and, crucially, the remaining gaps that this study aims to fill. Similar issues arise in the Results and Discussion, where comparisons are limited largely to a land-based Blanco FM catalog. Please approach either Václav Kuna or Yu Ren as their FM catalogs are better constrained by a decent OBS network. Besides, OBS-based focal mechanism catalogs from other OTFs would provide more

appropriate and informative comparisons.
In addition, several studies focused specifically on the G3 region itself are not discussed. For example, the manuscript discussed the role of pore fluids in barrier zones, but this interpretation has already been supported by multidisciplinary work, including a recent study by Chesley et al. (2025). Engaging more directly with these studies would significantly strengthen the Discussion.

Comment 5: Finally, I have concerns about the way the ultimate conclusions are framed. The Abstract, Introduction, and Discussion emphasize that the observed diversity of focal mechanisms demonstrates that OTFs are non-conservative plate boundaries. While this is true, it is no longer a particularly novel conclusion. Since Grevemeyer et al. (2021), numerous multidisciplinary studies have repeatedly supported this view, including several cited above - especially these seismological observations also revealed diverse faulting styles of OTFs. I would find it unfortunate if the manuscript ultimately centers on reaffirming this general concept. Instead, I encourage the authors to focus more explicitly on what new insights are gained from this study, particularly those that emerge from high-resolution OBS-based focal mechanisms, and to place these findings in a comparative context across different OTFs.

Version 1:

Reviewer comments:

Reviewer #1

(Remarks to the Author)
Review for revised manuscript ‘Focal mechanism variations reveal mechanical controls on segmentation at oceanic transform faults’ by F. Tan, W. Fan, P. M. Shearer, M. D. Behn, and J. J. McGuire for publication in Nature Communications - initial title ‘Variations in mechanical properties control segmentation of oceanic transform faults.’

This is a review for a revised version of the manuscript. I will thus not repeat summarizing the content of the manuscript. My overall, very positive evaluation of the manuscript has not changed.

I appreciate that the authors considered my comments and implemented them. The authors responded to all of my comments thoroughly.

Focusing on focal mechanism variability (spatial and temporal) and its relation to the physical mechanisms of faulting along the Gofar has strengthened the text.

I appreciate that the authors added examples of waveform polarity picks in the supplement and the substantial re-analysis of the P-wave polarity estimation using a Deep Learning picker.

As said before, this is a significant contribution with noteworthy results. I have a few questions.

Best regards,
Jochen Braunmiller

——

L112-121 and in general. Changing the P-wave polarity estimation processing changed the number of focal mechanisms from about 3,100 to about 2,300 solutions. The authors argue this is mainly due to implementing stricter criteria for accepting a focal mechanism. I think this is fine. What is slightly more concerning is that the relative distribution of the mechanism types changed considerably relative to the initial manuscript (SS now 30%, was 21%, N and R are now together ~10%, but were ~20%). I assume this has to do with the criteria for using or not using a particular mechanism or polarity. In the rebuttal, it may have been useful to indicate how many events that are in both focal mechanism catalogs changed category. Ideally, that number is small, showing that at the core, the catalog is robust. The claim of mechanism variability and its link to changes in mechanical properties are central to the manuscript. Changing the automatic polarity estimation workflow has changed the focal mechanism dataset somewhat. I think the overall story is not affected, but it would be reassuring for the review process to confirm that the changes are insignificant.

I also have a general comment regarding the mechanism classification. In Figure 2, red and blue show reverse and normal events. What is the difference between a reverse and a normal event if both, P and T, have a plunge near 45 degrees? Those vertical dip-slip mechanisms are actually very similar: simply changing the dip of the shallow plane by a few degrees, which is likely within the uncertainties, and they could be the same. The Zoback, ML (1980, 1992) definitions for faulting style eliminate these types of mechanisms for further consideration (for SHmax). — Perhaps explain why you keep the vertical dip-slip events, and perhaps tell how many events (%-wise) are of that type. They do represent variability, okay, but whether one calls them ‘normal’ or ‘reverse’ is a bit arbitrary (given uncertainties)

In the supplement, could you briefly explain why you needed to repick the P onsets? In the initial manuscript, this was clear as the polarity was estimated from a few samples after the onset. But how does RPNet use the onsets? Please explain.

——

A few small edits (line numbers refer to the new manuscript):

L35. Last line of abstract, perhaps ‘properties are governing segmentation.’?

L61. Not sure about the ‘,’ between ‘systematic’ and ‘faulting.’

L67-68. Perhaps change slightly to: ‘… that each rupture, on average, in M5.5 earthquakes every five years [1, 2 …’

L125. Thanks for fixing the formula for R. I had missed that (but probably all formulas involving the three principal stresses and some ratio of them could be considered a shape function).

L129-132. Comment. We used the consistency of T-axes between strike-slip and normal faulting to separate pull-apart extension from intra-transform spreading for the Blanco (Braunmiller & Nabelek, 2008); the tensional component of the stress regime in the pull-aparts has the same direction as for the transform part (and is rotated 45 degrees for the central intra-spreading ridge). This is somewhat similar to your observation that the orientation of the principal horizontal stress axis is the same for the R and N events relative to SS (P axis for R, T axis for N events)

L318-326. This reads as if critically stressed crust is a phenomenon associated with OTFs, but not so much with continental faults. If the crust in continental crust is equally close to ‘critical,’ then why would one expect fewer foreshocks there than in the oceanic environment? Please clarify.

Methods.
L428-430. You may want to briefly explain ‘fault plane uncertainty, ‘mechanism probability,’ and ’station distribution ratio,’ otherwise all readers interested in this detail will have to go through references [32, 66].

L474. ‘before the M6 mainshock …’ The text does not mention that the mainshock happened on 18 September 2008; please add the mainshock date somewhere.

Figure 3. In a), perhaps you could add some percentiles relative to the median curves to show tightness/variability?

Supplement.

L25. Missing reference to EQTransformer

L33-35. The sentence reads as if you used PickBlue only for 100 events. But that is not true, correct? You used PickBlue to repick all P-phases from the Gong and Fan (2022) catalog for the polarity estimation with RPNet, correct?

Figure S1. Thanks for including the examples. Most have high phase probability, and overall, the events are likely larger than the average event. — Please add to the caption that the traces are aligned on the PickBlue P pick (at 2.5 s). — For these cases, are all polarities consistent with the focal mechanism shown? If so, please say that in the caption. If not, you could indicate inconsistent polarities in the figure - like adding a symbol - and explain in the caption. — What I am wondering is whether the inconsistent picks are associated with the traces that I would not pick polarities manually (that is okay) or whether they include some/many clear picks (not so great). — I wonder, based on the overall high Phase probability, how well some of the smaller events are resolved, but perhaps Figure S15 is the best attempt at an answer to that.

Figure S2-4. Perhaps you could add dashed lines that separate zones 1-5 of G3?

Figure S11. The small p-values for zones 3 (in particular) and 4 are intriguing. What drives these values? By eye - admittedly not a great way to evaluate this - zones 2 and 3 differences look quite similar, yet the p-values are different. For zones 1 and 5, and perhaps 4, I see changes. For 2 and 3, I am not so convinced.

Figure S12. Caption, second line ‘patche’ - remove ‘e’.

Figure S15. Since you have this handy, you could add the same table listing the number of picks for each cell? You started with 598. I wonder how many picks are left in the lower right. This is not really central to anything, and the authors may ignore this comment.

Figure S16. Perhaps add “N = X” in each subplot to indicate how many focal mechanisms fall in each group.

Reviewer #2

(Remarks to the Author)

I received a second round of reviews for the manuscript, with the new title: “Focal mechanism variations reveal mechanical controls on segmentation at oceanic transform faults”. In the new version, the authors have made a significant number of changes to the text and analyses compared to the original manuscript. The authors have addressed all or most of my

previous comments. The new version presents a very improved and more robust analysis of the Gofar transform, using the final 2323 events after applying rigorous quality control. Also, the new discussion compares the local seismicity of the Gofar with other local seismicity databases of Blanco and Chain transforms on text, and more oceanic transform faults on Table S1, which are in different oceans. I assume that I am really suppressed by the significant improvement. I am very satisfied with the new version and results. I will recommend that the manuscript be accepted, after applying the minor changes/improvements described below.

Minor changes

Maintext
I recommend changing the "Relevance across oceanic transform systems" in the Discussion to "Comparison with other oceanic transform faults". There is a clear difference in the literature between using the term "transform system" and "transform fault". For example, Gofar is a transform multi-segmented system, but Chain is a single transform fault segment. So, please, change the title.

Supplement

Figure S1: I really liked your updated Figure S1, showing with more detail the examples of the polarities analyzed. Based on your supplement file with a list of the events, you have a magnitude starting ~0.3. So, please, include also an example of a seismogram for that magnitude, or with magnitude 0.5, as well as any event with magnitude 1.5 and 2.5. From that, we will see more clearly the variation of polarity quality compared to seismic noise across the entire magnitude range.

Table S1: You must change the structure of your table to better clarify the oceanic transform faults with mechanisms available in the literature. For example, you say "7 transforms along the Mid-Atlantic Ridge" for Engeln et al., JGR. What are the 7 transforms? You did not describe. They are 8, actually: Doldrumns, Vema, Kane, Hayes, Charlie-Gibbs, St.Paul, Romanche, Chain. You can see the number of focal mechanisms for each one of them on paper. Also, I request to include the new 3rd column in the table, informing the type of data source used by each study. Studies like Elgen et al 1986; Bergman and Solomon 1988; Abercrombie and Ekstrom 2001, plus others, used teleseismic records, while studies like Ren et al 2023; Schlaphorst et al 2023; Grevemeyer et al 2025; and your study use local OBS data. So, this difference must be clarified. Please, restructure your table to be more robust in global comparison. You can follow this example, which I did:

Name Number Data References
Romanche 14 Teleseismic Abercrombie and Ekstrom 2001; Engeln 1981
Chain 98 OBS/Teleseismic Schlaphorst et al 2023; Abercrombie and Ekstrom 2001; Engeln 1981
St. Paul 45 Teleseismic de Melo et al 2025; Wolfe et al 1993; Engeln 1981
Charlie-Gibbs 8 Teleseismic Aderhold and Abercrombie 2016 ; Engeln 1981
Jan Mayen 4 Teleseismic Rodriguez-Perez and Ottemoller 2014
Doldrums 5 Teleseismic de Melo et al 2024; Engeln 1981
Vema 4 Teleseismic Engeln 1981
Hayes 4 Teleseismic Engeln 1981
1520 4 Teleseismic Engeln 1981
… … … …

Following this example, and incorporating these additional references and transform faults, please reclassify the other transform faults already included in your table. If you intend to present a robust global comparison of focal mechanisms, all the information available from the literature should also be compiled and presented in detail and robustly. This would demonstrate that your study is the single transform fault investigation to analyze an extensive number of focal mechanisms like that, thereby highlighting the novelty and significance of your research.

Aderhold, K., & Abercrombie, R. E. (2016). The 2015 Mw 7.1 earthquake on the Charlie-Gibbs transform fault: Repeating earthquakes and multimodal slip on a slow oceanic transform. Geophysical Research Letters, 43(12), 6119-6128.

Rodríguez-Pérez, Q., & Ottemöller, L. (2014). Source study of the Jan Mayen transform fault strike-slip earthquakes. Tectonophysics, 628, 71-84.

de Melo, G. W. S., Mitchell, N. C., & Sokolov, S. Y. (2024). The 2020 Mw 6.6 Vernadsky transform earthquake sequence: rupture and Coulomb stress changes surrounding an oceanic core complex. Marine Geophysical Research, 45(4), 28.

de Melo, G. W. S., Grevemeyer, I., Liu, S., Maia, M., & Rüpke, L. (2025). Seismicity and thermal structure of the St. Paul Transform System, equatorial Atlantic: insights from focal depth analysis. Solid Earth, 16(10), 1059-1072.

Wolfe, C. J., Bergman, E. A., & Solomon, S. C. (1993). Oceanic transform earthquakes with unusual mechanisms or locations: Relation to fault geometry and state of stress in the adjacent lithosphere. Journal of Geophysical Research: Solid Earth, 98(B9), 16187-16211.

Reviewer #3

(Remarks to the Author)
I appreciate the authors' continued efforts to revise and improve the manuscript. Several of my previous concerns have been thoughtfully addressed, and I acknowledge the progress made. However, a number of substantive issues raised in my prior review remain unresolved, and I outline these below along with some additional comments.

Comment 1: Outstanding Major Concerns
(1) Justification for re-estimation of local magnitudes and P-phase picks
The authors state in their response that local magnitudes were recalculated and that PickBlue was used for re-picking, yet neither the main text nor the supplementary materials provide any methodological detail or quantitative justification for these choices. What distinguishes the new magnitude estimates from those in Gong and Fan (2022)? More importantly, if the re-picking with PickBlue yields meaningfully different results from EQTransformer on this specific dataset, which would be necessary to justify the additional processing, this should be demonstrated explicitly. Simply asserting that PickBlue performs better on OBS data in general is not sufficient; the improvement needs to be shown for the dataset. Furthermore, if the phase picks differ substantially, one might reasonably ask why event locations from Gong and Fan (2022) were not also re-estimated. I encourage the authors to address these points clearly in the revised manuscript.
(2) Failure of the workflow for large-magnitude events
It remains unclear why the focal mechanism workflow failed to produce reliable estimates for large events, including the M6 mainshock, given that the catalog is restricted to M < 4.5 events. This is counterintuitive, as larger events typically produce stronger, more easily interpretable signals. The absence of any explanation for this behavior is a notable gap, particularly since demonstrating that the workflow performs as expected for well-constrained events is important for validating its overall reliability. I strongly encourage the authors to include a dedicated discussion of this issue.
(3) Probability thresholds for phase picking and polarity
The choice of a 0.3 probability threshold for P-phase picking, combined with a 0.95 threshold for polarity, raises concerns about consistency. While I understand the motivation to maximize catalog size, quality should take precedence over quantity, especially given the limited number of functional stations in this OBS network. After reviewing Text S1 and Fig. S15, I would recommend the authors consult recent literature on the performance of machine learning pickers, for example:
José Enrique García, Luis M. Fernández-Prieto, Antonio Villaseñor, Verónica Sanz, Jean-Baptiste Ammirati, Eduardo A. Díaz Suárez, Carmen García; Performance of Deep Learning Pickers in Routine Network Processing Applications. Seismological Research Letters 2022;; 93 (5): 2529–2542. doi: https://doi.org/10.1785/0220210323
Bornstein, T., Lange, D., Münchmeyer, J., Woollam, J., Rietbrock, A., Barcheck, G., et al. (2024). PickBlue: Seismic phase picking for ocean bottom seismometers with deep learning. Earth and Space Science, 11, e2023EA003332. https://doi.org/10.1029/2023EA003332

Since the authors claimed the reason for the PickBlue re-picking was the improved performance, I don‘t understand why a higher P-phase probability was not used. Based on Fig. S15, raising the P-phase probability threshold to ≥ 0.6 (while retaining the 0.95 polarity threshold) would yield consistency above 90% between automated and manual picks. Although this may reduce catalog size, it would considerably strengthen the reliability and defensibility of the results.
(4) The 8A network
The authors' claim that the 8A network would not meaningfully contribute to this study because it contains only 7 stations does not appear to be accurate. A review of the IRIS website (https://ds.iris.edu/gmap/#network=8A&starttime=2019-01-01T00:00:00&endtime=2022-12-31T23:59:59&planet=earth) reveals a considerably larger number of stations. While differences in network geometry are acknowledged, a comparison of focal mechanisms in the spatial overlap between the two networks could provide a valuable cross-validation opportunity and would strengthen the paper. I encourage the authors to revisit this point.
(5) References
The referencing could be improved in several places. Lines 43–47 of the Introduction draw almost exclusively from GOFAR-focused studies, despite opening with a broader perspective on oceanic transform faults. The authors are encouraged to incorporate references from other well-studied OTFs. Additionally, the citation of the [18] JGR paper at line 60 seems misplaced, as that study was not based on OBS data. Finally, the claim in lines 62–64 regarding a "debated" topic would benefit from additional details as well as specific supporting references.

Comment 2: I was encouraged to see the addition of this new subsection, "Relevance across transform fault systems", as cross-OTF comparison could be a genuinely interesting dimension of this work. However, in its current form, the section largely restates previously published observations (e.g., along-strike variations in faulting style along Blanco and Chain) without offering new insight or synthesis. I recognize that the catalog size may preclude the kind of kilometer-scale statistics achievable for GOFAR, but I believe there is room for more targeted quantitative comparisons and cross-scale discussion. I encourage the authors to develop this section further to deliver on its promise.

Overall, this manuscript presents an interesting and potentially valuable focal mechanism catalog for the westernmost GOFAR. However, due to the unresolved concerns outlined above, I am unable to recommend it for acceptance (with the current form) at Nature Communications. Given the scope and style of the contribution, the authors may also wish to consider whether a disciplinary journal might be a more natural venue for this work.

Version 2:

Reviewer comments:

Reviewer #3

(Remarks to the Author)
I am pleased to receive this revised manuscript and appreciate the authors' continued efforts throughout the review process. The work has improved considerably, and I believe it will make a significant contribution to the field and attract broad interest from the geophysical community.

I have only one remaining concern - While the authors briefly explained in their rebuttal letter why local magnitudes were recalculated relative to the previous catalog, I was unable to find any corresponding addition or clarification in either the main text or the supplementary materials. Given that the present study builds directly upon the earlier catalog, and given that the authors themselves acknowledged certain technical issues with the original magnitude estimates, I believe this point deserves explicit documentation in the manuscript.

I kindly ask the authors to add a dedicated section or note in the supplement that clearly addresses the following: (1) what the specific technical issues were with the magnitude estimation in the previous catalog; (2) what methodology was adopted in the present study to address these issues; and (3) a quantitative assessment of the extent to which the revised magnitudes differ from or improve upon the original estimates.

I want to emphasize that this does not affect the main analyses or conclusions of the paper. However, transparency about such technical corrections is important for reproducibility and for readers who may wish to build upon this catalog in future work. Addressing this point carefully will further strengthen an already solid contribution.

Open Access This Peer Review File is licensed under a Creative Commons Attribution 4.0 International License, which permits use, sharing, adaptation, distribution and reproduction in any medium or format, as long as you give appropriate

credit to the original author(s) and the source, provide a link to the Creative Commons license, and indicate if changes were made.
In cases where reviewers are anonymous, credit should be given to 'Anonymous Referee' and the source.
The images or other third party material in this Peer Review File are included in the article's Creative Commons license, unless indicated otherwise in a credit line to the material. If material is not included in the article's Creative Commons license and your intended use is not permitted by statutory regulation or exceeds the permitted use, you will need to obtain permission directly from the copyright holder.
To view a copy of this license, visit https://creativecommons.org/licenses/by/4.0/

We thank the editor and reviewers for their constructive and thorough evaluation. We have carefully addressed all concerns and substantially revised the manuscript. Before describing the specific changes, we articulate the significance of our study and why we believe this study merits publication in Nature Communications.

Oceanic transform faults show the most systematic and predictable earthquake behaviors on Earth, with quasi-periodic M5.5+ earthquakes repeating on the same fault patches every few years at the East Pacific Rise. This predictability has made them a focus for understanding the fundamental physics of the earthquake cycle. Yet a central puzzle has persisted: what controls the along-strike segmentation that dictates where large earthquakes occur and where they stop? Prior work has established that rupture zones and creeping barriers differ in seismic velocity, stress drop, and Vp/Vs, but these observations are indirect and have remained disparate, lacking a unifying mechanical explanation.

Our study addresses this problem by exploiting the unprecedented scale of a 2,323-event focal mechanism catalog, an order of magnitude larger than any previous catalog for an OTF. We show that faulting-type proportions vary systematically along strike despite a uniform stress field, and that this variation directly tracks the segmentation between locked rupture zones and creeping barriers. No previous study has resolved these within-transform patterns; prior catalogs, comprising a few to ~100 mechanisms per OTF, documented diverse faulting styles but were too small to robustly identify the systematic spatial structure that we report. This observational advance represents a shift from descriptive documentation of mechanism diversity to a diagnosis of fault zone mechanical state.

The patterns we observe carry a clear mechanical interpretation. Through Coulomb failure modeling, we show that barrier zones with depleted strike-slip populations are mechanically weak, with elevated pore-fluid pressures that reduce effective normal stress, promote aseismic slip, and inhibit the stress accumulation required for large earthquake rupture. This conclusion is independently corroborated by controlled-source electromagnetic imaging of deep fluid penetration beneath the barrier zone, by along-strike variations in seismic structure and stress drop, and by numerical models of hydrothermal fluid modulation of fault slip. Our finding resolves a longstanding question about why barrier zones persistently terminate rupture on OTFs: they are not merely geometric discontinuities but mechanically distinct domains governed by different fluid conditions. This framework unifies previously diverse geophysical observations under a single physical mechanism and provides, for the first time, a direct link between faulting-type statistics and the mechanical properties that control the partitioning of seismic and aseismic slip on OTFs.

Beyond the spatial patterns, we show that the fault network evolves through the seismic cycle. The M6 mainshock activated secondary structures that were relatively quiescent during the interseismic period, and the foreshock sequence produced detectable shifts in faulting-type

distributions within the barrier zone. That the fault network sits close to failure and can slip by modest stress perturbations offers a mechanical explanation for the order of magnitude higher foreshock rates observed on OTFs compared to continental strike-slip faults. This connection has implications for understanding earthquake nucleation and short-term predictability on these faults.

Our cross-OTF comparison with the Blanco and Chain transforms shows that along-strike clustering of faulting types is not unique to Gofar and is observed across fast, intermediate, and slow spreading rates. While the smaller catalog sizes at these other faults do not permit the same statistical analysis, the qualitative consistency supports the generality of mechanical controls on OTF segmentation and motivates future OBS deployments and related studies to test this hypothesis.

We believe this combination of observational scale, mechanistic insight, and broad relevance places the study at the forefront of current understanding of transform fault mechanics and warrants consideration in Nature Communications.

In this revision, we now analyze all 31,251 cataloged earthquakes reported by Gong and Fan (2022), implement a new deep-learning polarity picker with multi-parameter quality control, and validate the workflow through visual inspection (Text S1), a random polarity test (Text S2), and sensitivity analysis across quality control thresholds (Text S3). The manuscript no longer centers on the non-conservative nature of OTFs; instead, it is organized around the contributions described above. We compare Gofar with Blanco and Chain using a uniform classification scheme (Fig. 6, Table S1) and discuss the results in a dedicated subsection. Figure 1 now shows the full Gofar system, and the Introduction explains the five-zone segmentation and motivating questions before presenting our objectives. All references suggested by the reviewers have been incorporated. Detailed point-by-point responses are described below with reviewers' comments shown in black while our replies shown in red.

Fengzhou Tan on behalf of the authors

Reviewer #1

General questions:

- For the focal mechanism classification (Kaverina diagram and classification scheme by Alvarez Gomez): Given all possible double couple solutions, would each group contain 1/7 of all events? I am not sure but Figure 1 bottom inset suggests different types may occur with different likelihood for a random draw. This does not affect the focal mechanism variability that you observe but could affect interpretation of histograms and percentages (slighly). I am just wondering whether the classification makes it 'more difficult' for an event to be declared SS than let's say N or SS-R. For an evenly-spaced distribution of double couple focal mechanisms, would 1/7 be classified as SS?

Thanks for the comment. We address this question with a random polarity pick test (Text S2, Fig. S17), in which each polarity pick is replaced with a random up/down assignment while preserving the network geometry and event locations. In the random test, N and R faulting types each account for around 20%, while SS is less than 10%, confirming that the seven classification groups do not have equal prior probabilities. Importantly, the random test shows a nearly uniform distribution along strike with no spatial pattern, and Kagan angles with respect to the mainshock are consistently large (~80 degrees). All these synthetic results (Fig. S17) are distinctively different from the observed catalog (Figs. 2 and 3), confirming that the spatial patterns we report cannot be explained by the classification or the OBS network geometry alone. We have added supplementary Text S2 to describe this numerical test and referenced it in the Method section (lines 443-444).

- Are there any noticeable (or significant) changes in mechanisms with event depth? The manuscript includes several cross-sections (Figs. 1b, 4b,e) but the text does not cover this aspect. Why?

Most earthquakes are located in a narrow depth range from 4 to 6.5 km, within the ~6-km-thick oceanic crust at G3, which limits the ability to resolve depth-dependent variations in faulting types over most of the fault. However, the barrier zone (Zone 2) has two layers of seismicity, with the deep layer extending into the uppermost mantle. Focal mechanisms of the shallow and deep seismicity are distinct, showing that deeper seismicity has more consistent SS-R and SS-N mechanisms in the west and east parts, respectively, while the shallow clusters show a more varied distribution (as shown in Figs. 4 and S16). We now discuss the depth dependence of focal mechanisms in lines 210-220 and have added Fig. S16 accordingly.

- What, in detail, is the angular difference attributed to Hardebeck and Hauksson [2001]? Their paper does not (sufficiently) define it. The Kagan angle is also a measure of (deviatoric moment

tensor) difference and is used throughout the manuscript. What does the 'inter-event angular difference' add (or why is it preferred for some instances in the manuscript)?

We have added two subsections in Methods (lines 446-468) to clearly define both metrics and explain their distinct roles. The Kagan angle between each small earthquake and the mainshock quantifies how different each small earthquake is from the mainshock; high values indicate that the cluster mechanisms differ from the mainshock. The interevent Kagan angle, computed between all event pairs within a spatial bin, quantifies how diverse the focal mechanisms are within that bin; high values indicate that events are mutually dissimilar, suggesting activation of multiple fault structures with different orientations. These two metrics provide complementary information. For example, a cluster that has consistent normal-faulting mechanisms would have high Kagan angles relative to the strike-slip mainshock (indicating different faulting type) but low inter-event angles (indicating a coherent, persistent faulting type within the cluster).

Main Text:

Line L94 and L122. The spacing is not clear. In line L94 it says '2x2 km along strike' but line L91 mentions a ~ 5 km wide strike-normal spread. Were there several bins at a given along-strike distance to cover the width? Or did you restrict the analysis to events within +-1 km of the central fault trace? — Similarly for L122. What width have the 1-km spatial bins? — This becomes clearer when looking at the figures in the main text and supplement and perhaps that is already sufficient.

We apologize for the confusion. The G3 fault-zone is about 4-km wide across the fault at the strike-normal direction, and we include all events within this fault zone for all cross-section plots and along-strike analysis. This zone is now shown explicitly in Fig. 1 and described in the caption. The 1-km along-strike bins each span the full 4-km across-strike width, and are used for Kagan angle and focal mechanism diversity calculations. These are described in the Methods section (lines 451-459).

For stress inversion, all focal mechanisms are used and are not limited to the 4-km width, with a grid defined in latitude and longitude rather than along-strike distance. We have clarified these descriptions in the Methods section (lines 470-471).

L275-287. This sounds interesting but is not well-developed. What does the co-existence of valleys and compressional bends imply for the suggested 'magma overshoot'? The valley is a reflection of lack of magma overshoot with the bends being some (other) structural feature unrelated to magma supply? It seems there's magma 'overshoot' and 'undershoot' at the same time but different locations? — That part is not clear.

We agree that this section was underdeveloped. In the revised manuscript, we have removed the bathymetry/magma overshoot discussion from the Discussion to maintain a tighter focus on the

mechanical controls on segmentation. The key point is that the G3 transform domain is dominated by a valley consistent with transtension, while local compressional bends and step-overs reflect internal fault architecture rather than magma supply. The point is now briefly noted in the Results section where the bathymetric context is most relevant (lines 214-215).

Figure 3. Perhaps change to ‘Average Kagan angle relative to the 2008 M6 mainshock in each …’ for people who only look at figures. — ‘The white curve and dots represent the average stress drop,’ change ‘black’ to ‘white’. — Comment: The largest Kagan angles seem to be in the strongly couples M6 zones Z3 and Z1; interesting.

Thanks for the comment. We have revised the Fig. 3 caption as suggested. The color reference has been corrected. We note that the reviewer's observation about large Kagan angles in Zones 1 and 3 refers to the post-mainshock period; before the mainshock, these zones have the smallest Kagan angles. This temporal change is discussed in the "Temporal Evolution" section (lines 221-268).

Methods section:

L312. Why only events with a reported magnitude? I assume because others in the catalog are smaller and less likely to have sufficient signal. You may want to say something.

In the revised analysis, we now process all 31,251 events reported by Gong and Fan (2022), regardless of whether a magnitude was originally assigned. The multi-parameter quality control criteria (including minimum 8 polarity picks, azimuthal gap < 180 degrees, and the 95th-percentile cutoffs on fault plane uncertainty, polarity misfit, and mechanism probability) effectively filter out events with insufficient signal quality. This approach is described in the Methods section (lines 397-433).

L322. You may want to add that the data were recorded with 100 sps. — Using 4 samples seems arbitrary and potentially unstable [I guess your results show otherwise], what was the reason for using such a short window and did you conduct tests to establish that 4 samples is a good number; also to start at the third sample after the pick. Perhaps add a sentence explaining the choice would be helpful. Perhaps point to the supplement as Fig. S1 shows how short the pulses are.

We thank the reviewer for the suggestion. Motivated by this comment, we have replaced the gradient-based polarity method with a machine-learning approach (RPNet; Han et al., 2025), which produces a probability score for each polarity pick. This approach is more robust than the previous gradient method and allows quantitative quality control through the probability threshold. The details are described in the Methods section (lines 416-419) and validated in Text S1.

Supplement:

Figure S1. Did you use the same 1D velocity model for calculating the piercing points as you used for the P onset picking? This is not clear, but you do not mention any other velocity model in the manuscript. — These are relatively strong, and deep aftershocks?

The reviewer is correct that we use the same local 1D velocity model for focal mechanism inversion. We have clarified the procedure in lines 422-423, and revised Fig. S1 to show three examples. These are examples selected for different focal mechanisms, including strike-slip, normal, and reverse events, instead of magnitude or depth.

Figure S2. The 'maximum horizontal stress direction' is very difficult to see in the top panel. As this is shown in the middle panel, perhaps eliminate from top panel? — Perhaps you could somewhere say explicitly what a stress ratio of 0.65 means in terms of the relative eiganvalue sizes?

Thanks for the suggestion. We have modified the figures accordingly. We also add a sentence to explain that a stress ratio close to 0.5 means a typical strike-slip regime (lines 126-127).

Figure S3. correct to 'same to those in Fig. S2' and again 'S2' in next sentence. (same correction in caption of Fig. S4)

Corrected.

Figure S6. 'The p>0.05 …' (same correction in caption of Fig. S7)

Figs. S5-S9 have been updated based on the new catalog, and all captions have been revised for consistency.

Figure S10. 'mainshock' correct spelling.

This figure has been removed. The faulting type distribution in each zone before and after the mainshock is now presented in Fig. 5a.

Figure S13. Last sentence in caption is garbled, do you mean '… is labelled …'?

Corrected. The figure is updated as Fig. S12.

Reviewer #2

The process is employed using an automatic machine learning–based phase-picking method, that compares the P phases comparing records in multiple ocean-bottom seismometers. The authors find a total of 3122 events (informed in Methodology). I was wondering how much the confidence of this method is. Did the authors double check the polarities manually after the automatic picking? In Method (line 321), the authors says: "We require a valid P pick to have probability >0.3.". This is a probability very low and can take phases that could be just hydroacoustic noise of some sea environmental or anthropogenic sources (e.g. Ship, Rain, Winds, Ice, or animals; Hildebrand 2009; Pieniazek et al 2023). It would be more confident results with a minimum of 60% (0.6 probability).

We appreciate this comment. Motivated by the reviewers' concerns, we have made two important methodological changes. First, we replaced the gradient-based polarity picking method with a machine-learning model (RPNet; Han et al., 2025), which provides a calibrated probability score for each polarity pick. Second, we require two thresholds to be satisfied jointly: a P-phase probability of >0.3 and a polarity probability of >0.95. The effective quality filter is therefore considerably stricter than the P-phase probability threshold of 0.3 alone would suggest.

To evaluate this workflow, we visually inspected 100 randomly selected events comprising 598 traces with P-phase probability above 0.1. We compared the automated polarity picks against manual labels across a broad range of threshold combinations (Fig. S15). With our preferred thresholds (phase probability of >0.3 and polarity probability of >0.95), the consistency with manual picks is approximately 86%. Raising the phase-probability threshold to 0.6 increases consistency to 91% but removes approximately 32% of the focal mechanism solutions. Importantly, the along-strike segmentation of faulting types and the spatial patterns in Kagan angle remains qualitatively unchanged whenever polarity consistency exceeds approximately 80%. We therefore retain the 0.3/0.95 combination to balance quantity and quality, and we rely on statistical patterns across hundreds of events rather than treating individual solutions as definitive. These results are described in Text S1 and Fig. S15, with key points discussed in the Methods section (lines 413-419).

Regarding noise contamination: our stations are deployed in an abyssal environment at depths exceeding 3,500 m, where anthropogenic and environmental noise sources (ship traffic, rain, wind, ice, marine fauna) are substantially attenuated relative to shallow-water or coastal settings. More importantly, our focal mechanism catalog is developed from an existing earthquake catalog that provides predicted P-wave arrival times based on known hypocenter locations, so we are picking polarities within a narrow 2-second window centered on the expected onset rather than searching for arrivals in continuous noise (lines 412-413).

Also, there are approximately 90 oceanic transform faults with multibeam data available (Ren 2022) to map their geomorphology in detail, including information on focal mechanisms. However, detailed local investigations using local OBS data have been well-conducted more recently, with recent approaches only in Chain (Leptokaropoulos et al 2023), Oceanographer (Grevemeyer 2025), and Blanco (Ren et al. 2023). This means that, even if the focal mechanisms are correct, it is not logical to reach a global conclusion about these varied deformation behaviors (thrust and normal mechanisms) determined at Gofar, but rather a local fault behavior. It is worth noting that of the 84 focal mechanisms obtained at Blanco from the manually picked P wave in events with local magnitudes above 3.5, approximately 80% were strike-slip deformation, with other minor events located outside the transform valley presenting different mechanisms (e.g., transtensional). In Chain transform, Leptokaropoulos et al (2023) shows that of the 88 focal mechanisms assessed with local OBS records of events with local magnitude >1.9, 57 had strike-slip focal mechanisms, with other compression mechanisms being shown in the morphological area of transgression. Similarly, Grevemeyer 2025 obtained manually assessed focal mechanisms only for events above 1.7 local magnitude. This concludes that the authors' machine learning results for such weak events, besides not allowing for a true global comparison, still do not demonstrate complete reliability when compared with results from a few other oceanic transform faults with more detailed analyses using mechanisms obtained from manual selections.

We agree that global conclusions should not rest on a single fault. To place our results in a broader context, we now compare our Gofar G3 catalog with the two other OTFs that have the largest OBS-based focal mechanism catalogs: the Blanco Ridge segment (31 mechanisms; Ren et al., 2023; Kuna, 2020) and the Chain transform (88 mechanisms; Leptokaropoulos et al., 2023; Schlaphorst et al., 2023). We summarize all published OTF focal mechanism catalogs in Table S1, showing that no other seismic datasets with 2000+ solutions exist. For a consistent comparison, we classify mechanisms from all three faults using the same seven-type scheme (Alvarez-Gomez, 2019). Under this uniform classification, the ~80% strike-slip fraction reported at Blanco is largely redistributed among SS, SS-N, and SS-R categories, because many events with oblique components that would traditionally be called "strike-slip" are separated into oblique subtypes. The result is that pure SS mechanisms account for approximately 30% of events on each OTF, consistent with that of Gofar G3 (Figs. 1 and 6). Moreover, both Blanco and Chain show along-strike variations in faulting type that are qualitatively consistent with mechanical property variations controlling the spatial distribution of mechanism types.

We have revised the title, and we have restructured the Discussion with a dedicated subsection ("Relevance across transform fault systems," lines 342-381) that presents these comparisons and their limitations transparently.

Line 39 – How much of the confidence of the 85% creeping (15% seismically active)? References like Shi et al (2022) did not compare all their relocations with local good multibeam data to calculate the creeping percent in more precise confidence. Also, most of the earthquakes were relocated and compared with bathymetry of very low resolution, and the authors relocated

relatively from teleseismic records, different of your case done better with local seismic data and good multibeam data. In addition, de Melo et al (2025, SE) found that 45-80% of the oceanic transform fault can be seismically active, using regional record of the earthquakes. So, I recommend you to remove this comment about the creeping percent of oceanic transform faults.

We have removed that statement.

Line 47 – Where did you take the full spreading rate of 140mm/year? I recommend you cite DeMets et al (2010).

We have added that citation (line 66).

Line 48-49 – McGuire (2008) shows that five years cycle time is expected to earthquakes with moment magnitude > 5.5. However, the authors compare the information to earthquake with magnitude 6 (M6). If you want to check the cycle of M6 in westernmost of Gofar, the author must inform the real time cycle for the same magnitude, which it will be ~20 years (see Figure 12 in McGuire 2008).

We have changed it to M5.5+, and revised the subsequent text accordingly. However, the reviewer is misunderstanding McGuire (2008). The M ~6.0 repeaters on the G3 segment were found to have repeat times of ~5 years in that study (Table S2, Figs 9 and 12), not 20 years.

Line 57 – Line 61 – In line 57, the authors call the local seismicity with low magnitude of "microearthquakes", while in line 61 the authors use the "microseismicity" term. I recommend you choose one single term and use always it. I know that it means the same thing, but Nature Communication papers are read by people from different areas, and your mix of terms can confuse the reader not expert in seismology. Also, I recommend you inform in Introduction the magnitude range of the local seismicity, then the reader will know link the low magnitudes with the "micro" term.

We now use "microearthquake" consistently throughout the manuscript and note in the Introduction (line 87) that these are mostly M < 3 events.

Line 92-96 – That are methodological information, and they should be places in Method chapter, not in main text.

We have deleted the technical description here for conciseness. Details are included in the Method section.

Lines 122-124 – This phrase should be moved to Method.

We have moved it to Methods and provided additional details there.

Lines 124-126 – Where is your mentioned mainshock rupture zone? The author did not include the information in Figures. Also, the mainshock epicenter shown in Figure 1 is located out of the transform fault and does not corroborate with your interpretations.

We have revised Fig. 1 to clearly show the mainshock rupture zone. The mainshock focal mechanism is now positioned within the transform fault zone.

Lines 143-144 – "are governed by mechanical parameter such as pore fluid pressure and internal frictional coefficient" -> Reference???

This conclusion comes from our Coulomb failure simulation. We have revised the text to make this clear and direct the reader to the "Coulomb Failure Simulation" subsection in Methods (lines 508-532).

Lines 147-148 – As additional, you could inform that the OTFs also present low rigidities in their mechanism and corroborate with fault weakening (de Melo et al 2025, GRL).

We now discuss the low rigidity of OTFs (de Melo et al., 2025) in the Discussion section (line 303), where it is used to contextualize the apparent mechanical weakness of OTFs globally.

Lines 155-156 – "…can explain the observed variations in stress-drop…." -> Where was it observed? Gofar? Indicate what zone are you talking about.

We have rephrased the text to specify that these observations are at Gofar G3 (line 189).

Lines 180-182 – The authors speculate in a global range, but they provide clear proves only about Gofar. These interpretations are not enough to convince that the behavior is in global level of OTFs.

This statement has been removed.

Lines 232-308 – The authors analysis local seismicity obtained by local stations in Gofar oceanic transform fault and provide new discoveries about Gofar along the paper. However, they start the discussion doing comparisons with continental transform faults. This comparison is weak and not enough to support mechanical behaviors in OTFs. If the authors want to give new discoveries and conclusion in global range of OTFs, they should provide evidence (e.g. variations in focal mechanism distribution) obtained in multiple different OTFs, not information in continents. That would include to check if the same behavior is visible in both slow, intermediate, and fast slipping OTFs.

The Discussion has been substantially restructured. It now opens with the scientific findings at Gofar, followed by comparisons with other OTFs (Blanco and Chain), and only then draws parallels with continental faults. The cross-OTF comparison is presented in a dedicated subsection "Relevance across transform fault systems" in the Discussion section.

Reviewer #3

Comment 1: My primary concern is on the data and methods used in this study.
(1) I was surprised to find that the OBS dataset is described in only 1-2 sentences. The authors

state that data from 16 OBS stations were used. However, it is known that not all stations in this experiment recorded high-quality or complete data throughout the deployment. Some stations reportedly failed shortly after deployment, and missing components are relatively common. Given these known complexities, the manuscript needs a much more detailed description of the OBS dataset, including station availability through time and data quality control. This information is critical because the study emphasizes spatiotemporal variations in faulting and stress. Changes in network geometry, station coverage, or component availability could bias the results if not carefully accounted for. The authors should explicitly demonstrate that the reported patterns are not artifacts of evolving network geometry or data completeness.

We have expanded the "Data" subsection in Methods to describe station availability in detail. Thirteen of 16 stations operated for most of the deployment with all three components available; three stations (G1, G11, and G15) failed at different times (Fig. S14). Because the network comprised only 16 stations, we retained data from all operational stations to maximize azimuthal coverage. When analyzing time-dependent variations, we explicitly account for potential changes in data quality by comparing focal mechanism uncertainty distributions before and after the mainshock in each zone (Figs. S5–S9) and applying statistical tests (KS test, chi-squared test) to assess the robustness of temporal changes. We conclude that the decreased focal mechanism similarity with the mainshock, the decreased proportion of pure SS events, and the increased focal mechanism diversity are robust in Zones 1, 3, and 5, where fault plane uncertainty does not increase after the mainshock. Additionally, the random polarity test (Text S2, Fig. S17) demonstrates that the spatial patterns cannot be explained by the network geometry alone.

(2) The focal mechanism catalog is constructed based on the microearthquake catalog of Gong and Fan (2022), which contains more than 30,000 events. In contrast, the present study analyzes only approximately 6,000 events. The manuscript does not explain why this relatively small subset was selected or what criteria led to the exclusion of the remaining events. In addition, the magnitude range of the analyzed events, from −0.55 to 4.3, indicates that the largest earthquakes, including the M6 events known to occur on Gofar and several events larger than M4.5, were excluded. This is surprising, as larger earthquakes typically have clearer onsets and more reliable polarity information and can serve as valuable benchmark cases for validating focal mechanism (FM) solutions. The authors should clearly justify the exclusion of large events and discuss whether their omission could affect the robustness or representativeness of the results.

In the revised analysis, we now process all 31,251 events reported by Gong and Fan (2022), including their manual catalog for large events. Because a large portion of that catalog does not have reported magnitudes, we recalculate the local magnitude for all events, resulting in only four earthquakes having M≥4.5 (including the M6 mainshock whose ML was determined to be 5.2 in the magnitude estimation). The multi-parameter quality control criteria yield a final catalog of 2,323 events with magnitudes from -0.3 to 4.4. The focal mechanism quality does not appear to correlate with earthquake magnitude. The mainshock and other M4.5+ events did not pass the quality control thresholds of our automatic workflow. Two of their focal mechanisms

are available from the Global CMT catalog (Ekstrom et al., 2012) and are consistent with the strike-slip mechanisms that dominate the mainshock zone in our catalog, providing independent validation of our approach.

(3) The authors re-picked the P arrivals using PickBlue, which seems to have better performance on OBS data. While this is reasonable, the manuscript does not explain why re-picking was necessary given that Gong and Fan (2022) already picked arrivals using EQTransformer. A comparison between EQTransformer and PickBlue picks would be very helpful to justify this step and to demonstrate how the new picks improve the catalog. Related questions that should be clarified include the following. Does the re-picking imply that the previous catalog has significant timing uncertainties? Were these earthquake re-located using the new picks? Were magnitudes re-estimated accordingly? Furthermore, I find it puzzling that a PickBlue probability threshold of 0.3 was adopted to define "valid" P picks, although Bornstein et al. (2024) recommended substantially higher thresholds to effectively remove outliers. The authors should explain why 0.3 is considered optimal in this case and provide evidence that this choice does not introduce excessive noise or bias. Although these points may appear technical, they directly affect the foundation of the FM catalog. These issues do not necessarily need to be fully detailed in the main text, but they should be carefully addressed, at least in the Methods or Supplementary Information, to validate the reliability of the dataset.

We clarify that we use the earthquake locations from Gong and Fan (2022) directly; no relocation is performed. Re-picking with PickBlue is necessary because the original catalog used EQTransformer, which was trained primarily on land-station data and produces less precise onset times in the OBS environment. Accurate P-wave onsets are critical for reliable first-motion polarity determination.

Regarding the threshold: we require two criteria jointly, a P-phase probability of 0.3 or greater and a polarity probability of 0.95 or greater. The effective filter is considerably stricter than the P-phase threshold alone. We have evaluated the performance through visual inspection of 100 randomly selected events (Text S1, Fig. S15). The preferred thresholds yield 86% consistency with manual picks. Raising the phase-probability threshold to 0.6 increases consistency to 91% but removes approximately 32% of solutions. The along-strike spatial patterns remain qualitatively unchanged whenever polarity consistency exceeds 80%. We therefore retain the 0.3/0.95 combination to balance quantity and quality. Further details are provided in Text S1 and discussed in the Methods section (lines 413-419).

(4) The resulting FM catalog shows diverse faulting styles along the G3 segment, which is potentially an interesting finding. However, before making strong statements about spatial variations in faulting style, the quality of the FM solutions needs to be more rigorously constrained. The cited work by Parnell-Turner et al. (2017) only accepted FMs with RMS fault plane uncertainty ≤35°, weighted fraction of misfit polarities <20%, station distribution radio >0.4, mechanism probability ≥0.6, and azimuthal gap ≤90°. These criteria are much more stricter

than the single criterion (i.e., azimuthal gap <180°) used in this study. Applying stricter, multi-parameter quality criteria would likely reduce the number of accepted mechanisms but would significantly increase confidence in the remaining solutions. In this context, quality should be prioritized over quantity.

We agree that rigorous quality control is essential. We now apply multi-parameter criteria: fault plane uncertainty of 45 degrees or less, polarity misfit of 23% or less, mechanism probability of 0.38 or more, station distribution ratio of 0.4 or more, and azimuthal gap of 180 degrees or less. For events with multiple solutions, we further require a probability difference of 0.1 or more between the preferred and alternative solutions. These thresholds are based on the 95th-percentile values of the respective parameter distributions (see Methods section). The resulting catalog contains 2,323 events (76% of 3,055 solvable events).

We have also tested stricter thresholds. Using the 90th-percentile cutoffs yields 2,032 events (67%), and the 80th-percentile cutoffs yield 1,652 events (54%). Under both stricter threshold sets, the overall faulting-type distribution and the along-strike spatial patterns remain largely consistent with those from the preferred catalog (Text S3). We note that directly adopting the thresholds from Parnell-Turner et al. (2017) would yield only 168 events, which would preclude the kilometer-scale statistical analysis that is central to our study. Given the 16-station network, stricter thresholds would not substantially improve individual solution quality (because the primary limitation is the network geometry) but would eliminate the sample size needed for along-strike analysis. We therefore rely on overall statistical patterns across hundreds of events to support our interpretation. These quality control procedures are described in the Methods section (lines 419-444).

Finally, I note that a more recent OBS deployment in the G3 segment, network code 8A, is now available. Combining data from both the ZD and 8A networks could substantially strengthen the conclusions regarding mechanical properties along G3 and would likely improve station coverage and azimuthal constraints.

Regarding the 8A network: this more recent deployment at G3 comprises only 7 independent stations, which does not meet our minimum requirement of 8 qualified polarity picks per event. The more recent deployment has a very different station distribution on G3 and would not provide similar focal sphere coverage even in the “Zone 2” barrier region. Integration of the two datasets is therefore not straightforward and is beyond the scope of the present study.

Comment 2: Based on the title, I initially expected the manuscript to include results from multiple oceanic transform faults. However, the study focuses exclusively on the western segment of the Gofar transform fault, G3. I am also not fully convinced by the statement that G3 is “representative” of global OTFs, which is supported only by brief citations to Wolfson-Schwehr et al. (2019) and Shi et al. (2021). These studies do not, in my view, provide strong justification for such a broad claim. Given the limited number of OBS experiments conducted at

OTFs, G3 is one of the best-studied examples, which allows for more detailed analysis, but this does not necessarily make it representative. The authors should either provide a much more detailed justification for this claim or avoid it altogether. Because the manuscript is tightly focused on a localized region, I strongly suggest revising the title to replace “oceanic transform faults” with “the western Gofar transform fault,” and modifying related statements throughout the manuscript accordingly.

We have revised the title to "Focal mechanism variations reveal mechanical controls on segmentation at oceanic transform faults." While the detailed observations are centered on Gofar, we retain "oceanic transform faults" in the title for two reasons. First, our finding that mechanical weakness due to elevated pore-fluid pressure controls the segmentation between rupture zones and creeping barriers addresses a question that applies to all OTFs, which ubiquitously exhibit this segmentation pattern. Second, our cross-OTF comparison (new subsection, lines 342-381) with Blanco and Chain demonstrates that along-strike clustering of faulting types is not unique to Gofar but is observed across fast, intermediate, and slow spreading rates, supporting the generality of mechanical controls on segmentation. As suggested, we have, however, removed all claims that G3 is "representative" of global OTFs and have revised the manuscript to clearly distinguish Gofar-specific observations from broader inferences.

Comment 3: The manuscript often assumes that readers are already familiar with previous works conducted in the area, or the study area itself. For example, the Introduction refers to the western strand of Gofar as “G3” without explaining how the overall fault system is segmented. This makes the manuscript difficult to follow for readers who are not specialists in this region. Including a clear map showing the location of the Gofar transform fault, its segmentation, and the position of G3 would greatly improve accessibility. Additionally, the manuscript states that G3 contains two seismic segments that rupture approximately every five years in M6 earthquakes (lines 48-49), but neither the segmentation nor the locations of historical M6 events are clearly illustrated. Showing these features on a map would provide essential context. Finally, while the authors note that G3 can be divided into five zones (lines 56-60), the manuscript would benefit from a brief discussion of the outstanding questions motivating this subdivision. This would create a clearer narrative bridge between prior studies and the specific objectives of the present work.

We thank the reviewer for the suggestion. We have substantially revised the Introduction to provide the necessary context for non-specialist readers. Fig. 1 now shows (a) the full Gofar transform system with its three segments (G1--G3) and historical M5.5+ earthquakes at G3, (b) the regional tectonic setting, (c) the OBS network and focal mechanism catalog, and (d) the five-zone segmentation along G3. The Introduction explains the segmentation and the outstanding questions that motivate this study before presenting our objectives (lines 65-85).

Comment 4: Several relevant studies are unfortunately overlooked. In the Introduction, the authors state that observations of faulting variations and stress regimes at OTFs are limited, without citing prior work. In fact, a number of studies, spanning several decades, have targeted at these topics, at least in part. Recent examples include but not limited to:
Kuna, V. (2020). Investigation of Slip and Tectonics of the Blanco Transform Fault using High-resolution Ocean Bottom Seismic Data. : Oregon State University.
Leptokaropoulos, K., Rychert, C.A., Harmon, N. et al. Broad fault zones enable deep fluid transport and limit earthquake magnitudes. Nat Commun 14, 5748 (2023). https://doi.org/10.1038/s41467-023-41403-6
Ren, Y., Lange, D., & Grevemeyer, I. (2023). Seismotectonics of the Blanco transform fault system, northeast Pacific: Evidence for an immature plate boundary. Journal of Geophysical Research: Solid Earth, 128, e2022JB026045. https://doi.org/10.1029/2022JB026045
Grevemeyer, I., Lange, D., Klaucke, I., Ruepke, L. H., Beniest, A., Gómez de la Peña, L., et al. (2025). Strike-slip versus extensional tectonics at the Oceanographer transform fault, Mid-Atlantic Ridge at 35°N. Journal of Geophysical Research: Solid Earth, 130, e2024JB030880. https://doi.org/10.1029/2024JB030880

These and other studies should be acknowledged to more clearly define the existing knowledge base and, crucially, the remaining gaps that this study aims to fill. Similar issues arise in the Results and Discussion, where comparisons are limited largely to a land-based Blanco FM catalog. Please approach either Václav Kuna or Yu Ren as their FM catalogs are better constrained by a decent OBS network. Besides, OBS-based focal mechanism catalogs from other OTFs would provide more appropriate and informative comparisons.

We thank the reviewer for the suggestion. The original manuscript was initially submitted to Nature Geoscience, which is much more space limited than Nature Communications. We have added citations to Kuna (2020), Leptokaropoulos et al. (2023), Ren et al. (2023), Grevemeyer et al. (2025), and Chesley et al. (2025), among others. The Introduction (lines 50-64) now discusses prior OBS-based focal mechanism studies to establish the existing knowledge base. The Discussion includes a detailed comparison with the Blanco and Chain transforms using OBS-based catalogs (Fig. 6), and engages directly with the Chesley et al. (2025) electromagnetic evidence for deep fluid penetration at Gofar. Table S1 summarizes all published OTF focal mechanism catalogs.

In addition, several studies focused specifically on the G3 region itself are not discussed. For example, the manuscript discussed the role of pore fluids in barrier zones, but this interpretation has already been supported by multidisciplinary work, including a recent study by Chesley et al. (2025). Engaging more directly with these studies would significantly strengthen the Discussion.

We thank the reviewer for the suggestion and have revised the Discussion accordingly (lines 282-284).

Comment 5: Finally, I have concerns about the way the ultimate conclusions are framed. The Abstract, Introduction, and Discussion emphasize that the observed diversity of focal mechanisms demonstrates that OTFs are non-conservative plate boundaries. While this is true, it is no longer a particularly novel conclusion. Since Grevemeyer et al. (2021), numerous multidisciplinary studies have repeatedly supported this view, including several cited above - especially these seismological observations also revealed diverse faulting styles of OTFs. I would find it unfortunate if the manuscript ultimately centers on reaffirming this general concept. Instead, I encourage the authors to focus more explicitly on what new insights are gained from this study, particularly those that emerge from high-resolution OBS-based focal mechanisms, and to place these findings in a comparative context across different OTFs.

This is a very helpful comment, and we have thoroughly restructured the manuscript in response. The revised Abstract, Introduction, and Discussion are centered on three new contributions:

(a) We demonstrate that faulting-type proportions vary systematically along strike within a single transform despite a uniform stress field, and that these variations track the independently identified segmentation between rupture zones and creeping barriers.

(b) Through Coulomb failure modeling, we establish a direct link between faulting-type statistics and inferred pore-fluid pressure, providing a physical framework for interpreting the mechanical state of the fault from focal mechanism data.

(c) We document how the seismic cycle modulates the fault network, with the M6 mainshock activating secondary structures across the fault and the foreshock sequence producing detectable shifts in faulting-type distributions within the barrier zone.

The emphasis on the non-conservative nature of OTFs has been removed. The Discussion is now organized into two subsections: "Mechanical controls on fault segmentation and slip behavior," which develops the new insights, and "Relevance across transform fault systems," which places the findings in a broader OTF and continental fault context.

Reviewer #1 (Remarks to the Author):

——

L112-121 and in general. Changing the P-wave polarity estimation processing changed the number of focal mechanisms from about 3,100 to about 2,300 solutions. The authors argue this is mainly due to implementing stricter criteria for accepting a focal mechanism. I think this is fine. What is slightly more concerning is that the relative distribution of the mechanism types changed considerably relative to the initial manuscript (SS now 30%, was 21%, N and R are now together ~10%, but were ~20%). I assume this has to do with the criteria for using or not using a particular mechanism or polarity. In the rebuttal, it may have been useful to indicate how many events that are in both focal mechanism catalogs changed category. Ideally, that number is small, showing that at the core, the catalog is robust. The claim of mechanism variability and its link to changes in mechanical properties are central to the manuscript. Changing the automatic polarity estimation workflow has changed the focal mechanism dataset somewhat. I think the overall story is not affected, but it would be reassuring for the review process to confirm that the changes are insignificant.

We thank the reviewer for this helpful comment. The observational basis for our interpretation is the statistical pattern of the focal-mechanism catalog, in particular the along-strike variation in faulting type, rather than any individual focal-mechanism solution. The revised workflow provides more reliable polarity picks, as confirmed by visual inspection, and applies stricter quality-control criteria, which yields higher-quality focal-mechanism solutions. This improvement is reflected in reduced average fault-plane uncertainty, from about 43° in the original catalog to about 35° in the revised catalog.

To assess the stability of the catalog directly, we compare the two catalogs for the 1,504 shared events. The differences in their faulting-type classifications fall predominantly between neighboring categories (for example, between SS and SS-R, or between N-SS and SS-N) rather than between the opposing dip-slip categories of normal and reverse (0.8%), which differ physically and rarely interchange. Counting same and adjacent categories together, 60% of the common events are classified consistently. The apparent change in the category proportions between the two full catalogs therefore reflects the threshold-dependent nature of the faulting-type classification together with the improved polarity estimation. Most importantly, the along-strike variation pattern that forms the basis of our interpretation is preserved in both catalogs, which indicates that the main conclusion is robust. To make this explicit in the manuscript, we have clarified

that the interpretation rests on the statistical along-strike pattern rather than on individual solutions (lines 480–481).

I also have a general comment regarding the mechanism classification. In Figure 2, red and blue show reverse and normal events. What is the difference between a reverse and a normal event if both, P and T, have a plunge near 45 degrees? Those vertical dip-slip mechanisms are actually very similar: simply changing the dip of the shallow plane by a few degrees, which is likely within the uncertainties, and they could be the same. The Zoback, ML (1980, 1992) definitions for faulting style eliminate these types of mechanisms for further consideration (for SHmax). — Perhaps explain why you keep the vertical dip-slip events, and perhaps tell how many events (%-wise) are of that type. They do represent variability, okay, but whether one calls them ‘normal’ or ‘reverse’ is a bit arbitrary (given uncertainties)

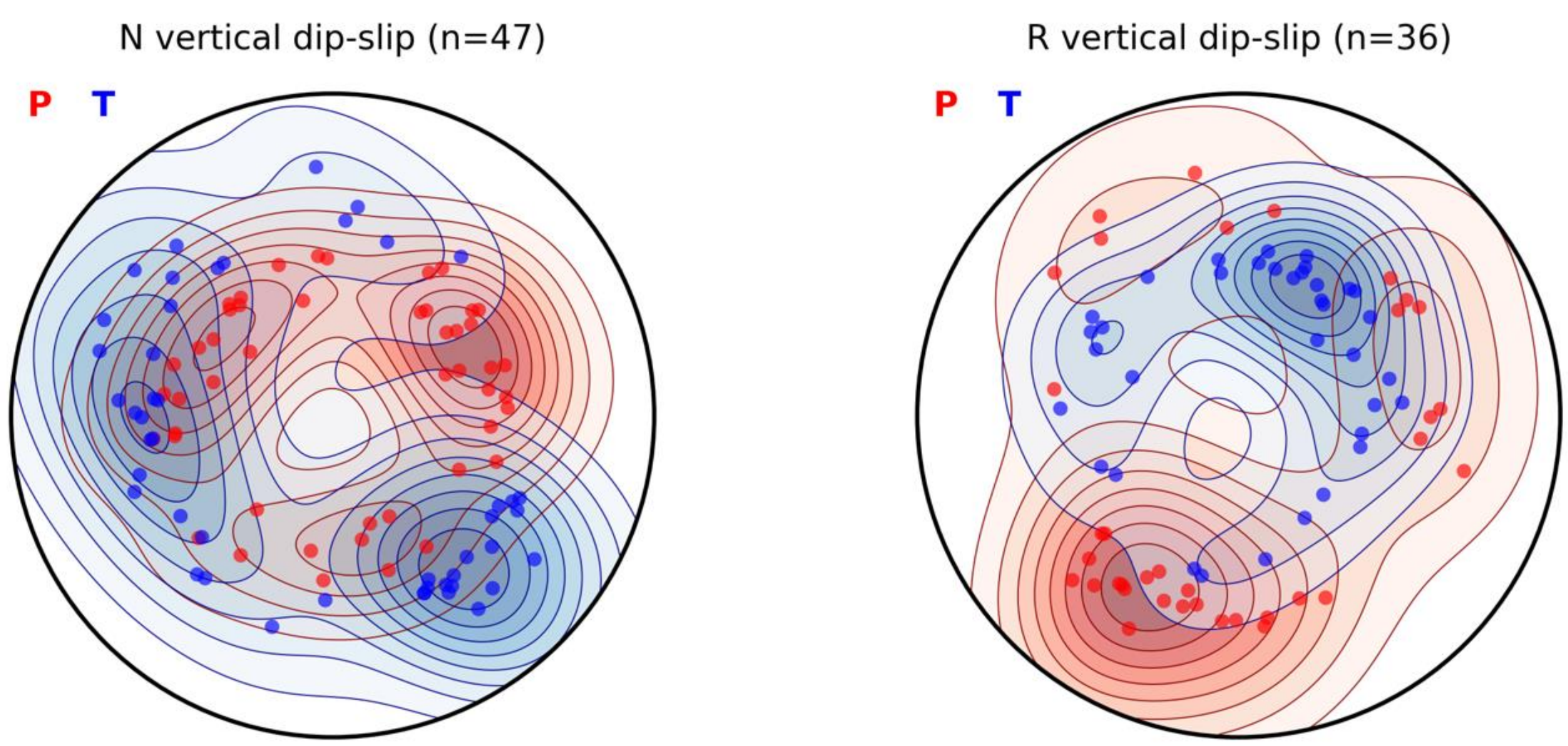

Fig. R1. Summary of P and T axis of vertical dip-slip events.

We thank the reviewer for raising this point. We agree that for near-vertical dip-slip mechanisms, the distinction between normal and reverse faulting can be sensitive to small changes in the dip of the nodal planes and may fall within the uncertainty of individual focal-mechanism solutions. To evaluate this issue, we identify vertical dip-slip events using a dip angle ≥70° and a rake angle between 70° and 110° or between −110° and −70°. In total, 83 events fall into this category, including 47 classified as normal and 36 classified as reverse, accounting for 3.6% of the catalog. Their P- and T-axis distributions (Fig. R1) show systematic patterns consistent with those of the full normal and reverse populations in Fig. 2: the T axes of the near-vertical normal events are concentrated in the same direction as the T axes of all normal events, whereas the

P axes of the near-vertical reverse events are concentrated in the same direction as the P axes of all reverse events. This indicates that the normal and reverse events remain distinguishable even among the near-vertical dip-slip mechanisms, and that their classification is not random. Because these events still carry useful information about the observed faulting types, we retain them in the analysis.

In the supplement, could you briefly explain why you needed to repick the P onsets? In the initial manuscript, this was clear as the polarity was estimated from a few samples after the onset. But how does RPNet use the onsets? Please explain.

We thank the reviewer for pointing this out. We have clarified in the Method section that, for RPNet, the input waveform window is centered on the P-wave onset time, which in our revised workflow is given by the PickBlue-PhaseNet pick (lines 446–448). Therefore, the P-wave onset time is required and important to define and align the input trace for polarity determination.

The purpose of re-picking the P-wave arrivals is to improve the accuracy and availability of P-wave onset picks used for first-motion polarity determination. We have added a brief explanation of the benefit of the PickBlue re-picking step to the Method section (Lines 444–446), expanded the related discussion in Text S1, and added a new Fig. S19 to provide an example. As shown in Fig. S19, four of the stations with valid P-wave onset picks and valid polarities in our workflow have no corresponding P-wave onsets in the original picks of Gong and Fan (2022). Without re-picking the P-wave onsets, this event would have only seven valid polarity picks and therefore would not satisfy the minimum requirement for resolving a focal mechanism. This example illustrates that re-picking the P-wave onsets can obtain valid polarity constraints that would otherwise be missed and affect the subsequent analysis.

——

A few small edits (line numbers refer to the new manuscript):

L35. Last line of abstract, perhaps ‘properties are governing segmentation.’?

The grammar has been corrected to “local mechanical properties govern segmentation”.

L61. Not sure about the ‘,’ between ‘systematic’ and ‘faulting.’

Deleted.

L67-68. Perhaps change slightly to: '… that each rupture, on average, in M5.5 earthquakes every five years [1, 2 …'

Modified as suggested (line 75).

L125. Thanks for fixing the formula for R. I had missed that (but probably all formulas involving the three principal stresses and some ratio of them could be considered a shape function).

Sorry for the confusion. We recently read the erratum to the stress inversion method paper (Skoumal et al., 2026), in which they state that the calculated stress ratio should be (sigma2-sigma3)/(sigma1-sigma3) instead. The definition difference is between R_ratio and (1 - R_ratio). Therefore, we need to correct this formula again in our paper accordingly. However, because our stress ratio is around 0.5, R_ratio and (1 - R_ratio) are almost the same value, and our interpretation based on that remains the same despite the change. Fig. S13 has been updated accordingly (as Fig. S14 in the revised supplementary material). The figure looks almost the same as before.

Robert J. Skoumal, Jeanne L. Hardebeck, Andrew J. Michael; *Erratum to* pySATSI: A Python Package for Computing Focal Mechanism Stress Inversions. *Seismological Research Letters* 2026; doi: https://doi.org/10.1785/0220260102

L129-132. Comment. We used the consistency of T-axes between strike-slip and normal faulting to separate pull-apart extension from intra-transform spreading for the Blanco (Braunmiller & Nabelek, 2008); the tensional component of the stress regime in the pull-aparts has the same direction as for the transform part (and is rotated 45 degrees for the central intra-spreading ridge). This is somewhat similar to your observation that the orientation of the principal horizontal stress axis is the same for the R and N events relative to SS (P axis for R, T axis for N events)

Thanks for your comment.

L318-326. This reads as if critically stressed crust is a phenomenon associated with OTFs, but not so much with continental faults. If the crust in continental crust is equally close to 'critical,' then why would one expect fewer foreshocks there than in the oceanic environment? Please clarify.

We have clarified the wording to avoid implying that critically stressed fault networks are unique to OTFs. Our intention was to emphasize that the high foreshock productivity of OTFs reflects the combination of a near-critical fault network and creep transients that

may be more readily generated in barrier zones modulated by fault-zone fluids. The text has been revised as follows: “In the OTF context, the pervasive near-critical state of the fault network, together with creep transients that may be more readily generated in the barrier zone due to fault-zone fluids [63], may explain an order of magnitude more foreshocks at OTFs than continental strike-slip faults [64]. These stress perturbations can cascade through the fault network [13], generating the characteristic foreshock swarms observed before large OTF earthquakes [13, 54].” (lines 327–333).

Methods.

L428-430. You may want to briefly explain ‘fault plane uncertainty, ‘mechanism probability,’ and ’station distribution ratio,’ otherwise all readers interested in this detail will have to go through references [32, 66].

We have added brief explanations for each metric according to their original definitions. Please see lines 458–465.

L474. ‘before the M6 mainshock …’ The text does not mention that the mainshock happened on 18 September 2008; please add the mainshock date somewhere.

We added the date in that sentence (line 513 of the revised manuscript).

Figure 3. In a), perhaps you could add some percentiles relative to the median curves to show tightness/variability?

We added shaded bands show the corresponding 25th-75th percentile ranges for each bin in Fig. 3a.

Supplement.

L25. Missing reference to EQTransformer

Fixed.

L33-35. The sentence reads as if you used PickBlue only for 100 events. But that is not true, correct? You used PickBlue to repick all P-phases from the Gong and Fan (2022) catalog for the polarity estimation with RPNet, correct?

Yes, we apply the PickBlue re-picking workflow to the entire dataset. For clarity, we have added “For a random-sampling visual inspection,” at the beginning of that

sentence. Please see line 44.

Figure S1. Thanks for including the examples. Most have high phase probability, and overall, the events are likely larger than the average event. — Please add to the caption that the traces are aligned on the PickBlue P pick (at 2.5 s). — For these cases, are all polarities consistent with the focal mechanism shown? If so, please say that in the caption. If not, you could indicate inconsistent polarities in the figure - like adding a symbol - and explain in the caption. — What I am wondering is whether the inconsistent picks are associated with the traces that I would not pick polarities manually (that is okay) or whether they include some/many clear picks (not so great). — I wonder, based on the overall high Phase probability, how well some of the smaller events are resolved, but perhaps Figure S15 is the best attempt at an answer to that.

We thank the reviewer for these helpful suggestions. We have revised the caption of Fig. S1 to state that the traces are aligned on the PickBlue P pick at 2.5 s. We also added Fig. S2, which provides three additional examples spanning a wider magnitude range, including a small event of M 0.4 and a large event of M 4.4. In Figs. S1 and S2, we added a bottom row showing the corresponding focal mechanisms with the picked polarities plotted on the beachballs. Most polarities are consistent with the focal-mechanism solutions, although some exceptions remain. These inconsistent polarities do not necessarily correspond to low-quality polarity picks; some are located close to nodal planes and may reflect uncertainty in ray paths as well as the uncertainty of the focal-mechanism solution itself. More generally, polarity-picking quality and focal-mechanism quality are not controlled by magnitude alone. Many small events have high signal-to-noise ratios, whereas some larger events occur within active sequences and can have lower signal-to-noise ratios. The quality-control criteria in our workflow are therefore important for evaluating the reliability of polarity picks and focal-mechanism solutions regardless of event magnitude.

Figure S2-4. Perhaps you could add dashed lines that separate zones 1-5 of G3?

Added as suggested.

Figure S11. The small p-values for zones 3 (in particular) and 4 are intriguing. What drives these values? By eye - admittedly not a great way to evaluate this - zones 2 and 3 differences look quite similar, yet the p-values are different. For zones 1 and 5, and perhaps 4, I see changes. For 2 and 3, I am not so convinced.

We thank the reviewer for this comment. The p-value is affected by both the degree of difference between the distributions and the number of event pairs included in the test.

Therefore, even when the differences between two distributions appear visually subtle, the large number of event pairs can still lead to statistically significant p-values. To make this clearer and help readers interpret the p-values, we have added the number of event pairs used in each comparison to the figure.

Figure S12. Caption, second line ‘patche’ - remove ‘e’.

Fixed.

Figure S15. Since you have this handy, you could add the same table listing the number of picks for each cell? You started with 598. I wonder how many picks are left in the lower right. This is not really central to anything, and the authors may ignore this comment.

This is an interesting suggestion. We have modified the figure to include the number of picks in each cell.

Figure S16. Perhaps add “N = X” in each subplot to indicate how many focal mechanisms fall in each group.

Added as suggested.

Reviewer #2 (Remarks to the Author):

Maintext

I recommend changing the "Relevance across oceanic transform systems" in the Discussion to "Comparison with other oceanic transform faults". There is a clear difference in the literature between using the term "transform system" and "transform fault". For example, Gofar is a transform multi-segmented system, but Chain is a single transform fault segment. So, please, change the title.

Modified as suggested.

Supplement

Figure S1: I really liked your updated Figure S1, showing with more detail the examples of the polarities analyzed. Based on your supplement file with a list of the events, you have a magnitude starting ~0.3. So, please, include also an example of a seismogram for that magnitude, or with magnitude 0.5, as well as any event with magnitude 1.5 and 2.5. From that, we will see more clearly the variation of polarity quality compared to seismic noise across the entire magnitude range.

We thank the reviewer for this helpful suggestion. We have added Fig. S2 to provide additional waveform examples over a broader magnitude range, including events of M 0.4, M 2.7, and M 4.4. This new figure is intended to show how the workflow performs across the magnitude range of the catalog. More generally, the quality of polarity picks and focal-mechanism solutions is not controlled by magnitude alone: many small events have high signal-to-noise ratios, whereas some larger events occur within active sequences and can have lower signal-to-noise ratios. The quality-control criteria in our workflow are therefore important for evaluating the reliability of polarity picks and focal-mechanism solutions regardless of event magnitude.

Table S1: You must change the structure of your table to better clarify the oceanic transform faults with mechanisms available in the literature. For example, you say "7 transforms along the Mid-Atlantic Ridge" for Engeln et al., JGR. What are the 7 transforms? You did not describe. They are 8, actually: Doldrumns, Vema, Kane, Hayes, Charlie-Gibbs, St.Paul, Romanche, Chain. You can see the number of focal mechanisms for each one of them on paper. Also, I request to include the new 3rd column in the table, informing the type of data source used by each study. Studies like Elgen et al 1986; Bergman and Solomon 1988; Abercrombie and Ekstrom 2001, plus

others, used teleseismic records, while studies like Ren et al 2023; Schlaphorst et al 2023; Grevemeyer et al 2025; and your study use local OBS data. So, this difference must be clarified. Please, restructure your table to be more robust in global comparison. You can follow this example, which I did:

Name Number Data References
Romanche 14 Teleseismic Abercrombie and Ekstrom 2001; Engeln 1981
Chain 98 OBS/Teleseismic Schlaphorst et al 2023; Abercrombie and Ekstrom 2001; Engeln 1981
St. Paul 45 Teleseismic de Melo et al 2025; Wolfe et al 1993; Engeln 1981
Charlie-Gibbs 8 Teleseismic Aderhold and Abercrombie 2016 ; Engeln 1981
Jan Mayen 4 Teleseismic Rodriguez-Perez and Ottemoller 2014
Doldrums 5 Teleseismic de Melo et al 2024; Engeln 1981
Vema 4 Teleseismic Engeln 1981
Hayes 4 Teleseismic Engeln 1981
1520 4 Teleseismic Engeln 1981
… … … …

Following this example, and incorporating these additional references and transform faults, please reclassify the other transform faults already included in your table. If you intend to present a robust global comparison of focal mechanisms, all the information available from the literature should also be compiled and presented in detail and robustly. This would demonstrate that your study is the single transform fault investigation to analyze an extensive number of focal mechanisms like that, thereby highlighting the novelty and significance of your research.

Aderhold, K., & Abercrombie, R. E. (2016). The 2015 Mw 7.1 earthquake on the Charlie-Gibbs transform fault: Repeating earthquakes and multimodal slip on a slow oceanic transform. Geophysical Research Letters, 43(12), 6119-6128.

Rodríguez-Pérez, Q., & Ottemöller, L. (2014). Source study of the Jan Mayen transform fault strike-slip earthquakes. Tectonophysics, 628, 71-84.

de Melo, G. W. S., Mitchell, N. C., & Sokolov, S. Y. (2024). The 2020 Mw 6.6 Vernadsky transform earthquake sequence: rupture and Coulomb stress changes surrounding an oceanic core complex. Marine Geophysical Research, 45(4), 28.

de Melo, G. W. S., Grevemeyer, I., Liu, S., Maia, M., & Rüpke, L. (2025). Seismicity and

thermal structure of the St. Paul Transform System, equatorial Atlantic: insights from focal depth analysis. Solid Earth, 16(10), 1059-1072.

Wolfe, C. J., Bergman, E. A., & Solomon, S. C. (1993). Oceanic transform earthquakes with unusual mechanisms or locations: Relation to fault geometry and state of stress in the adjacent lithosphere. Journal of Geophysical Research: Solid Earth, 98(B9), 16187-16211.

Thanks for the suggestion. We have revised the table following the example. We now explicitly list the names of each transform fault included in the referenced studies. We have also added the suggested references as well as additional relevant references. Please see the revised Table S1.

Reviewer #3 (Remarks to the Author):

Comment 1: Outstanding Major Concerns
(1) Justification for re-estimation of local magnitudes and P-phase picks
The authors state in their response that local magnitudes were recalculated and that PickBlue was used for re-picking, yet neither the main text nor the supplementary materials provide any methodological detail or quantitative justification for these choices. What distinguishes the new magnitude estimates from those in Gong and Fan (2022)? More importantly, if the re-picking with PickBlue yields meaningfully different results from EQTransformer on this specific dataset, which would be necessary to justify the additional processing, this should be demonstrated explicitly. Simply asserting that PickBlue performs better on OBS data in general is not sufficient; the improvement needs to be shown for the dataset. Furthermore, if the phase picks differ substantially, one might reasonably ask why event locations from Gong and Fan (2022) were not also re-estimated. I encourage the authors to address these points clearly in the revised manuscript.

We thank the reviewer for pointing out that the justification for the PickBlue re-picking was not sufficiently described in the previous version. We have added a brief explanation of the benefit of the PickBlue re-picking step to the Method section (lines 444–446), expanded the related discussion in Text S1, and added a new Fig. S19 to provide an example.

PickBlue was published after the study of Gong and Fan (2022) and was therefore unavailable for that work. The requirements for locating an event and for resolving a focal mechanism also differ in the number and type of measurements they need. The purpose of re-picking the P-wave arrivals is to obtain more P-wave onset picks for first-motion polarity determination. As shown in Fig. S19, four of the stations with valid P-wave onset picks and valid polarities in our workflow have no corresponding P-wave onsets in the original picks of Gong and Fan (2022). Without re-picking the P-wave onsets, this event would have only seven valid polarity picks, below the minimum eight required for resolving a focal mechanism. The re-picking therefore provides valid polarity constraints that would otherwise be missed and affect the subsequent analysis. Where P-wave picks are available in both catalogs, the differences are minor, with a median difference of -0.012 s (Fig. R2).

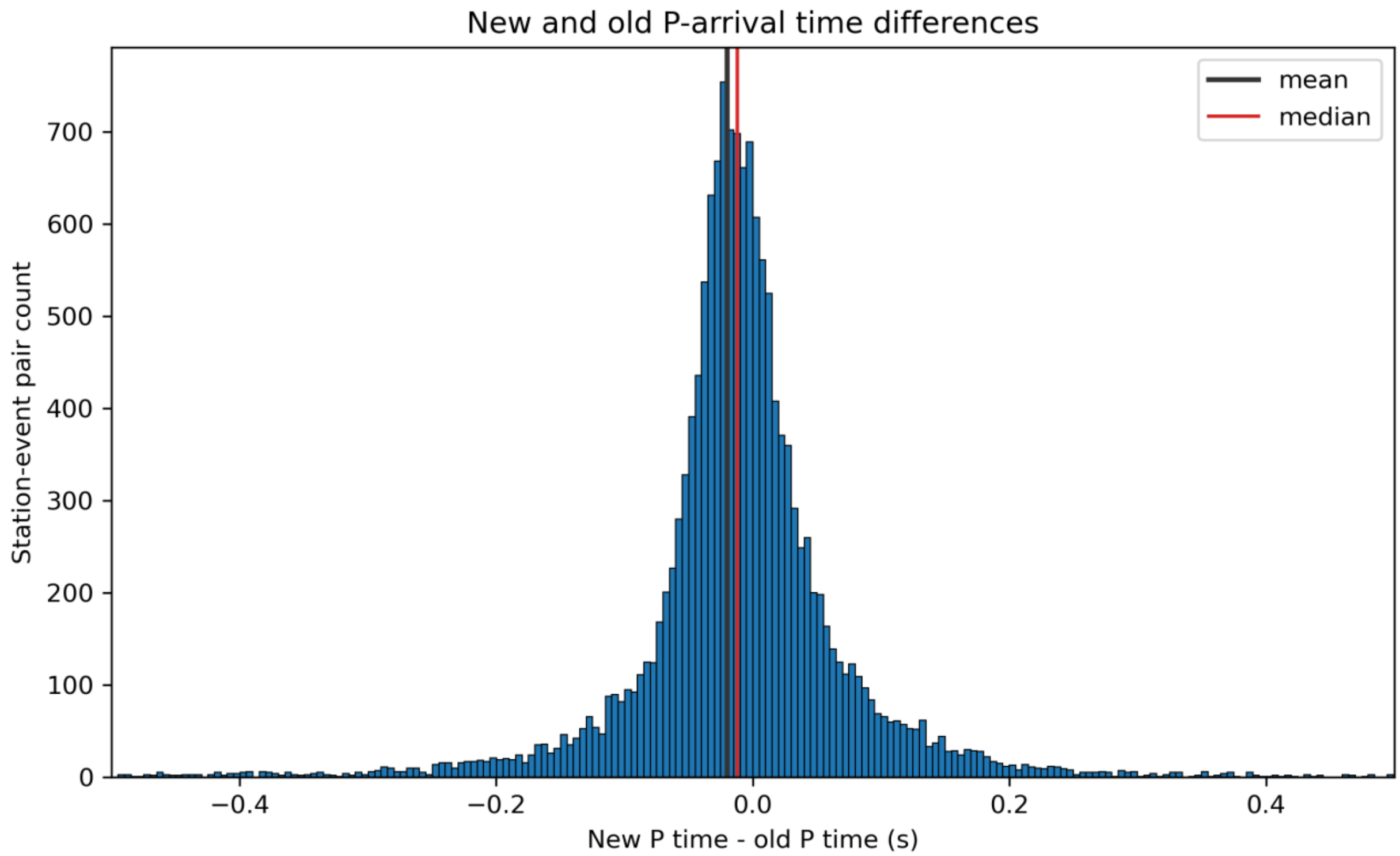

Fig. R2. Absolute picking time difference between this study and Gong and Fan (2022).

Re-estimating the event locations with the updated picks would not change the catalog appreciably. Gong and Fan (2022) located events with the COMPLOC method, which solves for source-specific station-term corrections, and their subsequent cross-correlation relocation further refined the locations. Events have an average of 4.4 S-wave picks, so the small variations in the P-wave picks are unlikely to change the final locations. We agree that quantifying the location uncertainty is a worthwhile direction, but a full relocation of all events would require a separate catalog-level investigation and is beyond the scope of the present study.

We recalculated the local magnitudes because we identified minor issues in the previous implementation related to waveform windowing and filtering that could introduce artifacts in the measured amplitudes. We re-estimated the local magnitudes using the same magnitude formulation as Gong and Fan (2022) with the corrected waveform-processing procedure. This recalculation is a technical correction to the magnitude values and does not affect the main analysis or conclusions of this study.

(2) Failure of the workflow for large-magnitude events

It remains unclear why the focal mechanism workflow failed to produce reliable estimates for large events, including the M6 mainshock, given that the catalog is restricted to M < 4.5 events. This is counterintuitive, as larger events typically produce

stronger, more easily interpretable signals. The absence of any explanation for this behavior is a notable gap, particularly since demonstrating that the workflow performs as expected for well-constrained events is important for validating its overall reliability. I strongly encourage the authors to include a dedicated discussion of this issue.

We thank the reviewer for pointing this out. The large events fail to yield reliable focal-mechanism solutions for different reasons at different stages of the workflow. For the M 6 mainshock, the deep-learning phase picker performed poorly, which is not unexpected for an event of this size, particularly due to the instrument movement caused by the shaking (Gong and Fan, 2022), and the event did not reach the minimum requirement of eight P-wave onset picks. We therefore use the GCMT solution of the M 6 mainshock as the reference in this study. Excluding the mainshock, three events have M>4.4. These events are all located near the western boundary of the study area connected to the ridge (the swarm zone), west of longitude -106.2, where poor azimuthal coverage leads to fault-plane uncertainties that exceed our selection criterion. These cases show that the reliability of focal-mechanism solutions is not controlled by magnitude alone. The quality-control criteria in our workflow, including the station geometry and fault-plane uncertainty, provide an effective way to evaluate the reliability of the focal-mechanism solutions regardless of event magnitude. We have added a sentence to the revised manuscript explaining why the three M>4.4 events do not pass our uncertainty criterion (lines 469–472), and we have added Fig. S2 to provide examples over a broader magnitude range.

(3) Probability thresholds for phase picking and polarity
The choice of a 0.3 probability threshold for P-phase picking, combined with a 0.95 threshold for polarity, raises concerns about consistency. While I understand the motivation to maximize catalog size, quality should take precedence over quantity, especially given the limited number of functional stations in this OBS network. After reviewing Text S1 and Fig. S15, I would recommend the authors consult recent literature on the performance of machine learning pickers, for example:
José Enrique García, Luis M. Fernández-Prieto, Antonio Villaseñor, Verónica Sanz, Jean-Baptiste Ammirati, Eduardo A. Díaz Suárez, Carmen García; Performance of Deep Learning Pickers in Routine Network Processing Applications. Seismological Research Letters 2022;; 93 (5): 2529–2542. doi: https://doi.org/10.1785/0220210323
Bornstein, T., Lange, D., Münchmeyer, J., Woollam, J., Rietbrock, A., Barcheck, G., et al. (2024). PickBlue: Seismic phase picking for ocean bottom seismometers with deep learning. Earth and Space Science, 11, e2023EA003332. https://doi.org/10.1029/2023EA003332

Since the authors claimed the reason for the PickBlue re-picking was the improved

performance, I don‘t understand why a higher P-phase probability was not used. Based on Fig. S15, raising the P-phase probability threshold to ≥ 0.6 (while retaining the 0.95 polarity threshold) would yield consistency above 90% between automated and manual picks. Although this may reduce catalog size, it would considerably strengthen the reliability and defensibility of the results.

We thank the reviewer for this thoughtful comment. Different deep-learning models have different architectures and are trained on different datasets, and their output probabilities are not necessarily calibrated in the same way, even though they all fall within the range of 0 to 1. Threshold values and performance metrics from one model are not directly transferable to another model. In this sense, the difference between the 0.3 PickBlue phase-picking threshold and the 0.95 polarity threshold does not indicate an inconsistency in the workflow; rather, each threshold should be evaluated in the context of the specific model output and quality-control step to which it is applied.

We are familiar with the papers suggested by the reviewer and agree that they provide useful guidance for evaluating machine-learning phase pickers. García et al. (2022) tested the original PhaseNet, EQTransformer, and Generalized phase detection models, and suggested that PhaseNet provides a good balance between sensitivity and a relatively low number of false detections, which led to their final choice of PhaseNet for continuous-data processing. The absolute threshold values and performance metrics from those models nonetheless cannot be transferred directly to PickBlue. Bornstein et al. (2024) trained and tested PickBlue, reported in their Fig. 8 that PickBlue-PhaseNet has only 5.5% outliers at a threshold of 0.3, with a missing rate of 7% for P onsets. In their final continuous-data processing, they adopted an even lower threshold of 0.1, indicating that lower probability thresholds can still be appropriate for PickBlue-PhaseNet P-wave onset picking. Our threshold of 0.3 is therefore already conservative for PickBlue-PhaseNet P-wave onset picking. Rasing the threshold to 0.6 would increase the missing rate to 18% according to their Fig. 8, more than doubling the missing rate relative to 0.3, while producing only a slight decrease in the outlier rate.

In our application, the along-strike segmentation of faulting types and the spatial patterns in Kagan angle remain qualitatively unchanged whenever the polarity consistency exceeds approximately 80%. Raising the phase-probability threshold to 0.6 would remove approximately 32% of the focal-mechanism solutions, which would reduce the statistical robustness of our analysis. Because of the limited station coverage of this OBS network, a higher phase-picking threshold would also reduce the number of polarity observations available for the retained events, and therefore would not necessarily improve the quality of the final focal-mechanism solutions. We also note

that, although the agreement between automatic and manual picks is an important measure of pick quality, manual picks are not an absolute ground truth for all traces.

For these reasons, we consider the current workflow, which uses a 0.3 threshold for PickBlue-PhaseNet phase picking, a 0.95 threshold for RPNet polarity determination, and subsequent focal-mechanism quality-control criteria, to be a reasonable choice for our analysis.

(4) The 8A network

The authors' claim that the 8A network would not meaningfully contribute to this study because it contains only 7 stations does not appear to be accurate. A review of the IRIS website (https://ds.iris.edu/gmap/#network=8A&starttime=2019-01-01T00:00:00&endtime=2022-12-31T23:59:59&planet=earth) reveals a considerably larger number of stations. While differences in network geometry are acknowledged, a comparison of focal mechanisms in the spatial overlap between the two networks could provide a valuable cross-validation opportunity and would strengthen the paper. I encourage the authors to revisit this point.

REDACTECD

Fig. R3 (copied from Fig 1 in Fan et al. 2026). Open triangles denote ocean-bottom seismometers: black for the 2008 experiment and red for the 2020 experiment.

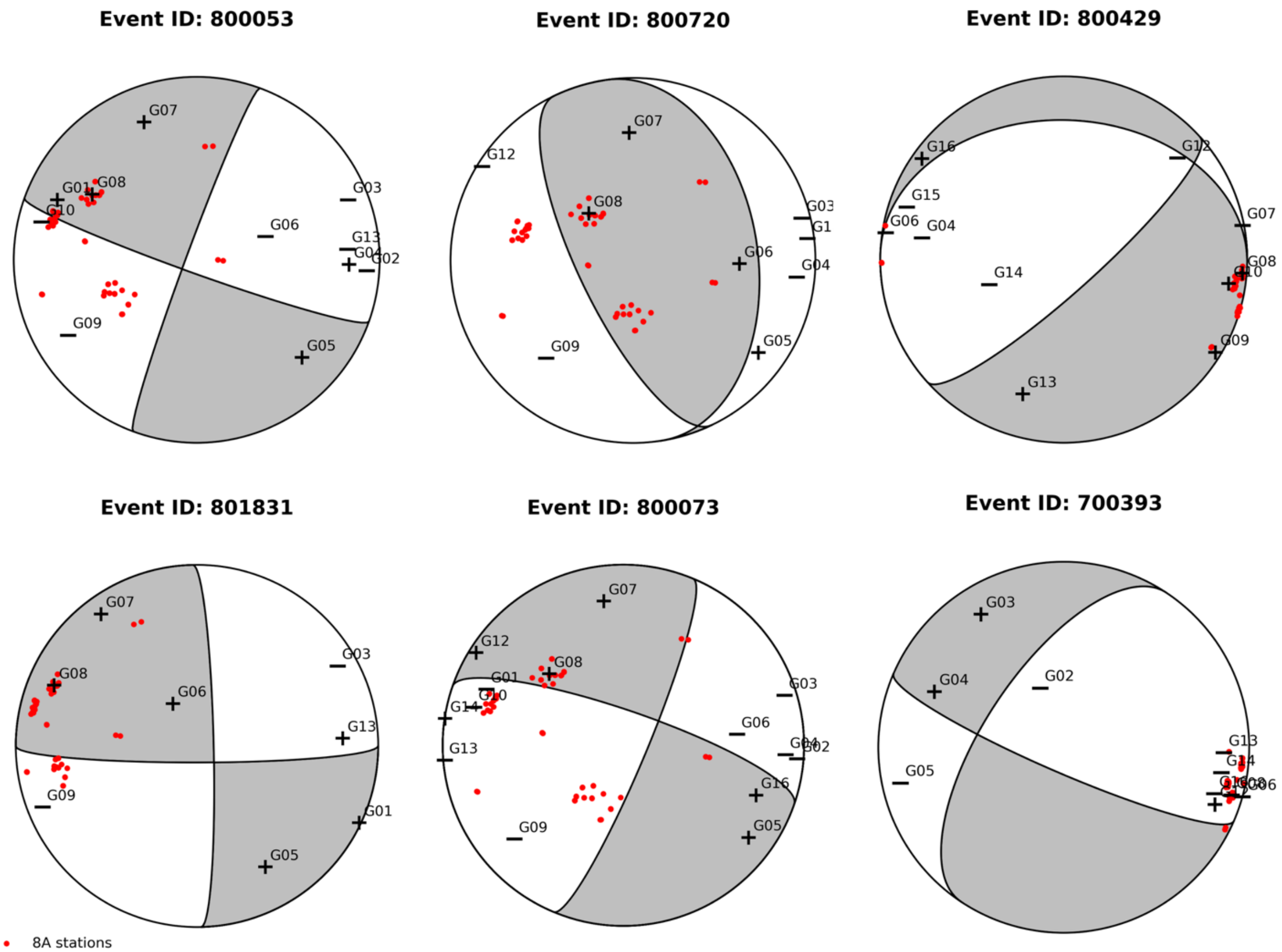

Fig. R4. Station distribution of the 8A array on example focal mechanism solutions. Red dots show the position of 8A stations.

We thank the reviewer for pointing this out. The reviewer is correct that the 8A network includes more stations than the number stated in our previous response. Our previous statement was intended to refer to the effective station groups rather than the total number of individual stations. As shown in Fig. R3, the 8A deployment comprises three mini-arrays with a spatial footprint of a few kilometers, which together provide only seven independent measurements. These stations are concentrated near the barrier zone and do not span the full extent of the G3 fault that is the focus of this study. We tested whether the 8A stations could resolve the focal mechanisms and found that, even when all available picks pass our quality-control criteria, they do not constrain the range of mechanisms obtained here (Fig. R4). For this reason, and consistent with the reviewer's point that quality matters more than quantity, we do not include the 8A stations in the focal-mechanism analysis. We note that a direct comparison in the spatial overlap would be attractive in principle, but the limited aperture and coverage of the 8A deployment do not support a meaningful cross-validation of the along-strike variation that our study resolves.

Fan, W., McGuire, J. J., Liu, Y., Behn, M. D., Warren, J. M., Collins, J. A., & Boettcher, M. S. (2026). Dynamics of rupture barriers on oceanic transform faults: Insights from the westernmost Gofar transform fault. Journal of Geophysical Research: Solid Earth, 131, e2025JB033439. https://doi.org/10.1029/ 2025JB033439)

(5) References
The referencing could be improved in several places. Lines 43–47 of the Introduction draw almost exclusively from GOFAR-focused studies, despite opening with a broader perspective on oceanic transform faults. The authors are encouraged to incorporate references from other well-studied OTFs. Additionally, the citation of the [18] JGR paper at line 60 seems misplaced, as that study was not based on OBS data. Finally, the claim in lines 62–64 regarding a "debated" topic would benefit from additional details as well as specific supporting references.

Lines 43-47: We have added references about detailed along-strike fault segmentation at Blanco, Discovery, Oceanographer, Chain, Romanche, and Charlie-Gibbs transform faults, in addition to Gofar. Please see lines 43-49.

Line 60: Here we refer to all previously mentioned studies including those with teleseismic data. We have changed “these studies” to “previous focal mechanism studies” to make this clearer. Please see line 59-60.

Lines 62-64: We have added clarification that observational limitations make the mechanisms sustaining along-strike segmentation difficult to determine and included additional relevant references. Please see lines 62-70.

Comment 2: I was encouraged to see the addition of this new subsection, "Relevance across transform fault systems", as cross-OTF comparison could be a genuinely interesting dimension of this work. However, in its current form, the section largely restates previously published observations (e.g., along-strike variations in faulting style along Blanco and Chain) without offering new insight or synthesis. I recognize that the catalog size may preclude the kind of kilometer-scale statistics achievable for GOFAR, but I believe there is room for more targeted quantitative comparisons and cross-scale discussion. I encourage the authors to develop this section further to deliver on its promise.

We thank the reviewer for this constructive suggestion. We agree that cross-OTF and cross-scale comparison is an important direction for developing this work further. A fully quantitative comparison across OTFs remains a broader community challenge, because

it requires focal-mechanism catalogs across multiple OTFs that are comparable in both quantity and quality and a comparison across catalogs of differing completeness and station coverage would conflate methodological differences with genuine differences in faulting behavior.

To strengthen the section while respecting this limitation, we have added a targeted comparison of focal mechanism diversity across the available OBS-based OTF focal mechanism catalogs with more than 10 events (Blanco, Chain, Oceanographer, and Gofar) at the scale of entire transform segments. The overall pattern is that OTFs with slower slip rate and larger age offset tend to have higher focal mechanism diversity. However, due to limited observations, we try not to overinterpret this and have framed it explicitly with caveats (lines 350–370). We have also noted that future progress will rely on additional data collection and systematic processing, which would allow both broad-scale and km-scale features to be resolved and compared, ultimately leading to a more general understanding of OTFs, faulting processes, and earthquake physics. We have added this discussion to the subsection (lines 395–398).

We thank reviewer #3 for the additional comments. Please see the response below.

Reviewer #3 (Remarks to the Author):

I am pleased to receive this revised manuscript and appreciate the authors' continued efforts throughout the review process. The work has improved considerably, and I believe it will make a significant contribution to the field and attract broad interest from the geophysical community.

I have only one remaining concern - While the authors briefly explained in their rebuttal letter why local magnitudes were recalculated relative to the previous catalog, I was unable to find any corresponding addition or clarification in either the main text or the supplementary materials. Given that the present study builds directly upon the earlier catalog, and given that the authors themselves acknowledged certain technical issues with the original magnitude estimates, I believe this point deserves explicit documentation in the manuscript.

I kindly ask the authors to add a dedicated section or note in the supplement that clearly addresses the following: (1) what the specific technical issues were with the magnitude estimation in the previous catalog; (2) what methodology was adopted in the present study to address these issues; and (3) a quantitative assessment of the extent to which the revised magnitudes differ from or improve upon the original estimates.

I want to emphasize that this does not affect the main analyses or conclusions of the paper. However, transparency about such technical corrections is important for reproducibility and for readers who may wish to build upon this catalog in future work. Addressing this point carefully will further strengthen an already solid contribution.

Response: We have added a dedicated section in the Supplementary Information (Text S4) that describes the technical issue affecting magnitude estimation in the original catalog, the revised procedure adopted in this study, and a quantitative comparison between the original and updated magnitudes. The updated calculation provides valid magnitudes for 30,092 events, compared with 6,395 events in the original catalog. For events with magnitudes available in both catalogs, the mean difference is 0.03. The estimated magnitude-of-completeness values are also comparable between the original and updated catalogs (0.6 and 0.4, respectively).

The manuscript titled *"Variations in mechanical properties control segmentation of oceanic transform faults"* shows results of focal mechanisms obtained in Gofar oceanic transform fault. The authors used a predicted P wave arrival times to find the P-phase and apply the re-pick using a 2s time around the predicted P-phase time, from seismic records obtained in 2008 by a set of ocean bottom seismometers deployed in Gofar. The process is employed using an automatic machine learning–based phase-picking method, that compares the P phases comparing records in multiple ocean-bottom seismometers. The authors find a total of 3122 events (informed in Methodology). I was wondering how much the confidence of this method is. Did the authors double check the polarities manually after the automatic picking? In Method (line 321), the authors says: "*We require a valid P pick to have probability >0.3.*". This is a probability very low and can take phases that could be just hydroacoustic noise of some sea environmental or anthropogenic sources (e.g. Ship, Rain, Winds, Ice, or animals; Hildebrand 2009; Pieniazek et al 2023). It would be more confident results with a minimum of 60% (0.6 probability). Also, there are approximately 90 oceanic transform faults with multibeam data available (Ren 2022) to map their geomorphology in detail, including information on focal mechanisms. However, detailed local investigations using local OBS data have been well-conducted more recently, with recent approaches only in Chain (Leptokaropoulos et al 2023), Oceanographer (Grevemeyer 2025), and Blanco (Ren et al. 2023). This means that, even if the focal mechanisms are correct, it is not logical to reach a global conclusion about these varied deformation behaviors (thrust and normal mechanisms) determined at Gofar, but rather a local fault behavior. It is worth noting that of the 84 focal mechanisms obtained at Blanco from the manually picked P wave in events with local magnitudes above 3.5, approximately 80% were strike-slid deformation, with other minor events located outside the transform valley presenting different mechanisms (e.g., transtensional). In Chain transform, Leptokaropoulos et al (2023) shows that of the 88 focal mechanisms assessed with local OBS records of events with local magnitude >1.9, 57 had strike-slip focal mechanisms, with other compression mechanisms being shown in the morphological area of transgression. Similarly, Grevemeyer 2025 obtained manually assessed focal mechanisms only for events above 1.7 local magnitude. This concludes that the authors' machine learning results for such weak events, besides not allowing for a true global comparison, still do not demonstrate complete reliability when compared with results from a few other oceanic transform faults with more detailed analyses using mechanisms obtained from manual selections.

The results of the authors collectively suggest that deformation behavior can vary between transform faults, being mostly strike-slip along the transform faults. Consequently, while promising the machine learning for too weak earthquake, may not yet provide a reliable basis for global-scale comparison without validation against such detailed local analyses. The author could transform the current manuscript in one more informative about the local mechanical behavior which occurs in Gofar transform, but not in global scale of oceanic transform faults. Therefore, the current manuscript version does not provide enough information to convince the current conclusions to publish in Nature Communication, and major reviews need to be done. I recommend rejecting and future versions be submitted to another geophysical journal. A few long comments are below:

Line 39 – How much of the confidence of the 85% creeping (15% seismically active)? References like Shi et al (2022) did not compare all their relocations with local good multibeam data to calculate the creeping percent in more precise confidence. Also, most of the earthquakes were relocated and compared with bathymetry of very low resolution, and the authors relocated relatively from teleseismic records, different of your case done better with local seismic data and good multibeam data. In addition, de Melo et al (2025, SE) found that 45-80% of the oceanic transform fault can be seismically active, using regional record of the earthquakes. So, I recommend you to remove this comment about the creeping percent of oceanic transform faults.

Line 47 – Where did you take the full spreading rate of 140mm/year? I recommend you cite DeMets et al (2010).

Line 48-49 – McGuire (2008) shows that five years cycle time is expected to earthquakes with moment magnitude > 5.5. However, the authors compare the information to earthquake with magnitude 6 (M6). If you want to check the cycle of M6 in westernmost of Gofar, the author must inform the real time cycle for the same magnitude, which it will be ~20 years (see Figure 12 in McGuire 2008).

Line 57 – Line 61 – In line 57, the authors call the local seismicity with low magnitude of "microearthquakes", while in line 61 the authors use the "microseismicity" term. I recommend you choose one single term and use always it. I know that it means the same thing, but Nature Communication papers are read by people from different areas, and your mix of terms can confuse the reader not expert in seismology. Also, I recommend you inform in Introduction the magnitude range of the local seismicity, then the reader will know link the low magnitudes with the "micro" term.

Line 92-06 – That are methodological information, and they should be places in Method chapter, not in main text.

Lines 122-124 – This phrase should be moved to Method.

Lines 124-126 – Where is your mentioned mainshock rupture zone? The author did not include the information in Figures. Also, the mainshock epicenter shown in Figure 1 is located out of the transform fault and does not corroborate with your interpretations.

Lines 143-144 – "are governed by mechanical parameter such as pore fluid pressure and internal frictional coefficient" -> Reference???

Lines 147-148 – As additional, you could inform that the OTFs also present low rigidities in their mechanism and corroborate with fault weakening (de Melo et al 2025, GRL).

Lines 155-156 – "...can explain the observed variations in stress-drop...." -> Where was it observed? Gofar? Indicate what zone are you talking about.

Lines 180-182 – The authors speculate in a global range, but they provide clear proves only about Gofar. These interpretations are not enough to convince that the behavior is in global level of OTFs.

Lines 232-308 – The authors analysis local seismicity obtained by local stations in Gofar oceanic transform fault and provide new discoveries about Gofar along the paper. However, they start the discussion doing comparisons with continental transform faults. This comparison is weak and not enough to support mechanical behaviors in OTFs. If the authors want to give new discoveries and conclusion in global range of OTFs, they should provide evidence (e.g. variations in focal mechanism distribution) obtained in multiple different OTFs, not information in continents. That would include to check if the same behavior is visible in both slow, intermediate, and fast slipping OTFs.

de Melo, G. W. S., Grevemeyer, I., Lange, D., Metz, D., & Kopp, H. (2025). Relationship between rupture length and magnitude of oceanic transform fault earthquakes. *Geophysical Research Letters*, *52*(1), e2024GL112891.

de Melo, G. W. S., Grevemeyer, I., Liu, S., Maia, M., & Rüpke, L. (2025). Seismicity and thermal structure of the St. Paul Transform System, equatorial Atlantic: insights from focal depth analysis. *Solid Earth*, *16*(10), 1059-1072.

DeMets, C., Gordon, R. G., & Argus, D. F. (2010). Geologically current plate motions. *Geophysical journal international*, *181*(1), 1-80.

Grevemeyer, I., Lange, D., Klaucke, I., Ruepke, L. H., Beniest, A., Gómez de la Peña, L., ... & Devey, C. W. (2025). Strike-slip versus extensional tectonics at the Oceanographer transform fault, Mid-Atlantic Ridge at 35 N. *Journal of Geophysical Research: Solid Earth*, *130*(7), e2024JB030880.

Hildebrand, J. A. (2009). Anthropogenic and natural sources of ambient noise in the ocean. *Marine Ecology Progress Series*, *395*, 5-20.

Leptokaropoulos, K., Rychert, C. A., Harmon, N., Schlaphorst, D., Grevemeyer, I., Kendall, J. M., & Singh, S. C. (2023). Broad fault zones enable deep fluid transport and limit earthquake magnitudes. *Nature Communications*, *14*(1), 5748.

McGuire, J. J. (2008). Seismic cycles and earthquake predictability on East Pacific Rise transform faults. *Bulletin of the Seismological Society of America*, *98*(3), 1067-1084.

Pieniazek, R. H., Beach, R. K., Dycha, G. M., Mickle, M. F., & Higgs, D. M. (2023). Navigating noisy waters: A review of field studies examining anthropogenic noise effects on wild fish. *The Journal of the Acoustical Society of America*, *154*(5), 2828-2842.

Ren, Y., Geersen, J., & Grevemeyer, I. (2022). Impact of spreading rate and age-offset on oceanic transform fault morphology. *Geophysical Research Letters*, *49*(2), e2021GL096170.

Ren, Y., Lange, D., & Grevemeyer, I. (2023). Seismotectonics of the Blanco transform fault system, northeast Pacific: Evidence for an immature plate boundary. *Journal of Geophysical Research: Solid Earth*, *128*(3), e2022JB026045.
